# Effectiveness of heterologous and homologous COVID-19 vaccination among immunocompromised individuals: a systematic literature review and meta-analysis

**DOI:** 10.1017/ash.2024.369

**Published:** 2024-09-26

**Authors:** Isabele Pardo, Aline Miho Maezato, Gustavo Yano Callado, Maria Celidonio Gutfreund, Mariana Kim Hsieh, Vivian Lin, Takaaki Kobayashi, Jorge L. Salinas, Aruna Subramanian, Michael B. Edmond, Daniel J. Diekema, Luiz Vicente Rizzo, Alexandre R. Marra

**Affiliations:** 1 Faculdade Israelita de Ciências da Saúde Albert Einstein, Hospital Israelita Albert Einstein, São Paulo, SP, Brazil; 2 Department of Internal Medicine, University of Iowa Carver College of Medicine, Iowa City, IA, USA; 3 Division of Infectious Diseases & Geographic Medicine, Stanford University, Stanford, CA, USA; 4 Department of Medicine, West Virginia University School of Medicine, Morgantown, WV, USA

## Abstract

**Objectives:**

We assessed the effectiveness of heterologous vaccination strategy in immunocompromised individuals regarding COVID-19 outcomes, comparing it to homologous approaches.

**Design:**

Systematic literature review/meta-analysis.

**Methods:**

We searched PubMed, CINAHL, EMBASE, Cochrane Central Register of Controlled Trials, Scopus, and Web of Science from January 1, 2020 to September 29, 2023. We included studies that evaluated the heterologous vaccination strategy on immunocompromised individuals through outcomes related to COVID-19 (levels of anti-SARS-CoV-2 spike protein IgG, neutralizing antibodies, symptomatic COVID-19 infection, hospitalization, and death) in comparison to homologous schemes. We also used random-effect models to produce pooled odds ratio estimates. Heterogeneity was investigated with I^2^ estimation.

**Results:**

Eighteen studies met the inclusion criteria for this systematic review. Fourteen of them provided quantitative data for inclusion in the meta-analysis on vaccine response, being four of them also included in the vaccine effectiveness meta-analysis. The vaccination strategies (heterologous vs homologous) showed no difference in the odds of developing anti-SARS-CoV-2 spike protein IgG (odds ratio 1.12 [95% Cl: 0.73–1.72]). Heterologous schemes also showed no difference in the production of neutralizing antibodies (odds ratio 1.48 [95% Cl: 0.72–3.05]) nor vaccine effectiveness in comparison to homologous schemes (odds ratio 1.52 [95% CI: 0.66–3.53]).

**Conclusions:**

Alternative heterologous COVID-19 vaccinations have shown equivalent antibody response rates and vaccine effectiveness to homologous schemes, potentially aiding global disparity of vaccine distribution.

## Graphical abstract

**Figure:**
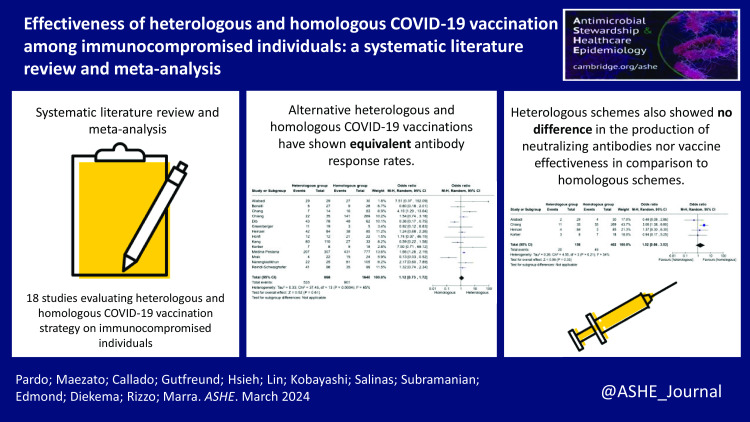

## Background

The COVID-19 pandemic emerged as a severe public health issue.^1^ SARS-CoV-2 infected 774 million people and caused 7 million deaths worldwide as of January 2024.^2^ The first vaccine against this disease was authorized by the US Food and Drug Administration (FDA) on December 11, 2020.^3^ Several studies have evaluated vaccine efficacy in healthy individuals or those with stable chronic medical conditions.^3^ However, immunocompromised individuals were excluded from trials during the early stages of the pandemic, leading to a lack of data on vaccine efficacy for this group.^4^ Recently, some investigations suggested that vaccine effectiveness and humoral immune response in immunocompromised individuals were lower than in immunocompetent people.^5,6^ Given higher COVID-19 complication and mortality rates among those immunocompromised,^7^ it is important to quantify vaccine effectiveness (VE) in this group and propose strategies to enhance immune response.

Currently, with new variants and evidence of reduced immunity induced by COVID-19 vaccines, booster doses are being administrated.^8^ However, obtaining boosters of the same type of COVID-19 vaccine sometimes poses a challenge due to inadequate access to mRNA vaccines in low- and middle-income countries, the rollout of Janssen or AstraZeneca vaccines followed by subsequent shortages of the primary vaccine types, or nationwide shifts to mRNA vaccines. Therefore, heterologous vaccination, where vaccines with different vectors or delivery systems from those used in the initial doses are administered as boosters, is employed. This approach has been adopted in many countries, enhancing vaccination flexibility and reducing vaccine inequity.^9^ Additionally, heterologous strategies may provide immunologic advantages to extend the breadth and longevity of protection.^10^ Therefore, studying the effectiveness of heterologous approaches is crucial for informing public health measures, particularly in countries where a diverse array of vaccines is not readily accessible.

We aimed to evaluate the effectiveness of heterologous vaccination on immunocompromised individuals through COVID-19 outcomes (levels of anti-SARS-CoV-2 spike protein IgG, neutralizing antibodies, symptomatic COVID-19, hospitalization, and death) in comparison to homologous approaches.

## Methods

### Systematic literature review and inclusion and exclusion criteria

**Figure 1.**
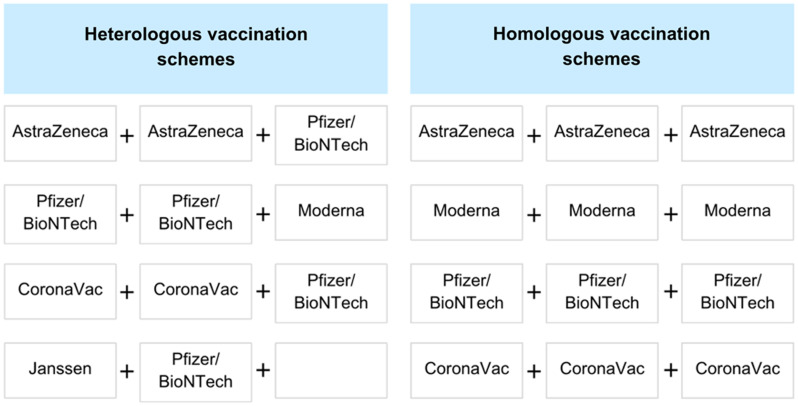
Examples of heterologous and homologous vaccination schemes. ***Note*:** One Janssen dose is equivalent to the primary series with two doses of other COVID-19 vaccines.

This review was conducted according to the Preferred Reporting Items for Systematic Reviews and Meta-Analysis (PRISMA) statement^11^ and the Meta-analysis of Observational Studies in Epidemiology (MOOSE) guidelines.^12^ This study was registered on Prospero (https://www.crd.york.ac.uk/PROSPERO/) on July 3, 2023 (registration number CRD42023440193). Institutional Review Board approval was not required. Immunocompromised individuals were defined as those treated with immunosuppressive medication (eg, corticosteroids, chemotherapy, or other immunosuppressive medications), chronic renal failure under hemodialysis, autoimmune and inflammatory rheumatic and musculoskeletal disease, solid organ transplant, hematopoietic stem cell transplant, HIV, or active cancer (current cancer, in treatment, or received diagnosis within last 12 months).^13,14^ Heterologous vaccination strategies were defined as schemes in which the booster dose has different vectors or delivery systems from the ones used in the primary series. Homologous strategies were defined as three doses of the same vaccine, with the same vectors and delivery systems (Figure 1). We only included those who got at least one booster. One Janssen dose is equivalent to the primary series with two doses of other COVID-19 vaccines. The review included manuscripts published from January 1, 2020 to September 29, 2023. There were no language restrictions. Inclusion criteria for studies in this systematic literature review were as follows: original research manuscripts; published in peer-reviewed, scientific journals; conducted in acute care settings that evaluated the effectiveness of heterologous versus homologous COVID-19 vaccines in immunocompromised individuals with randomized clinical trial design; and observational study design. Commentaries, studies with overlapping individuals, studies in pediatric populations, and studies in preprint were excluded. Studies in which there was no comparison between heterologous and homologous vaccination and evaluating less than 3 doses were excluded.

### Search strategy

We performed literature searches in PubMed, Cumulative Index to Nursing and Allied Health (CINAHL), Embase (Elsevier Platform), Cochrane Central Register of Controlled Trials, Scopus, and Web of Science. The entire search strategy is described in Supplementary Appendix 1. We reviewed the reference lists of retrieved articles to identify studies that were not identified from the preliminary literature searches. To filter the 16,252 articles obtained from the databases, titles and/or abstracts were assessed by two investigators (I.P and A.M.M.) to exclude articles using the inclusion criteria. All disparities were resolved through consensus.

### Data abstraction and quality assessment

Of six independent reviewers (I.P, A.R.M, G.Y.C, M.K.H, M.C.G, and V.L), two independently abstracted data for each article using a standardized abstraction form (Supplemental Form 1). Reviewers resolved disagreements by consensus. All reviewers recorded data on study design, publication year and calendar time, population selection, setting, analyzed vaccines, serological response definition, and side effects associated with vaccination. Our primary outcome was positive antibody response according to the cut-off presented by the analyzed study.

Secondary outcomes were to evaluate the vaccine effectiveness through the number of symptomatic SARS-CoV-2 infections after COVID-19 vaccines, as well as the number of hospitalizations, and deaths related to COVID-19. The risk of bias was assessed using the Downs and Black scale.^15^ Reviewers answered all original questions except for question #27, which was modified to a yes or no. The highest possible score achievable on this scale was 28. Two authors performed the scale independently, and discrepancies were solved by consensus.

### Patient consent statement

The present investigation is a systematic literature review and meta-analysis of published data, so no patient informed consent was required.

### Statistical analysis

For the meta-analysis, we compared positive antibody responses between heterologous versus homologous vaccinations. We weighted each study for the analysis using the approach outlined by DerSimonian and Laird.^16^ We performed stratified analyses of the associations between anti-SARS-CoV-2 spike protein IgG production in different types of immunocompromised states, in studies that evaluated neutralizing antibodies, in studies with symptomatic COVID-19 after receiving the COVID-19 vaccines, and in studies classified as good per the Downs and Black score. We did not include studies that did not report the absolute number of individuals that produced anti-SARS-CoV-2 spike protein IgG after the third vaccine dose in our stratified analysis. We assessed heterogeneity between studies using both the *I* ^2^ statistic and the Cochran Q statistic test. We analyzed with the Cochrane Review Manager (RevMan) Web edition 4.12.0. To examine publication bias, we visually inspected a funnel plot using RevMan (Supplemental Figure 1A and 1B) and also evaluated by applying the Egger test with Comprehensive Meta-Analysis version 4 software (Englewood, NJ).

## Results

### Characteristics of included studies in the systematic literature review

**Figure 2.**
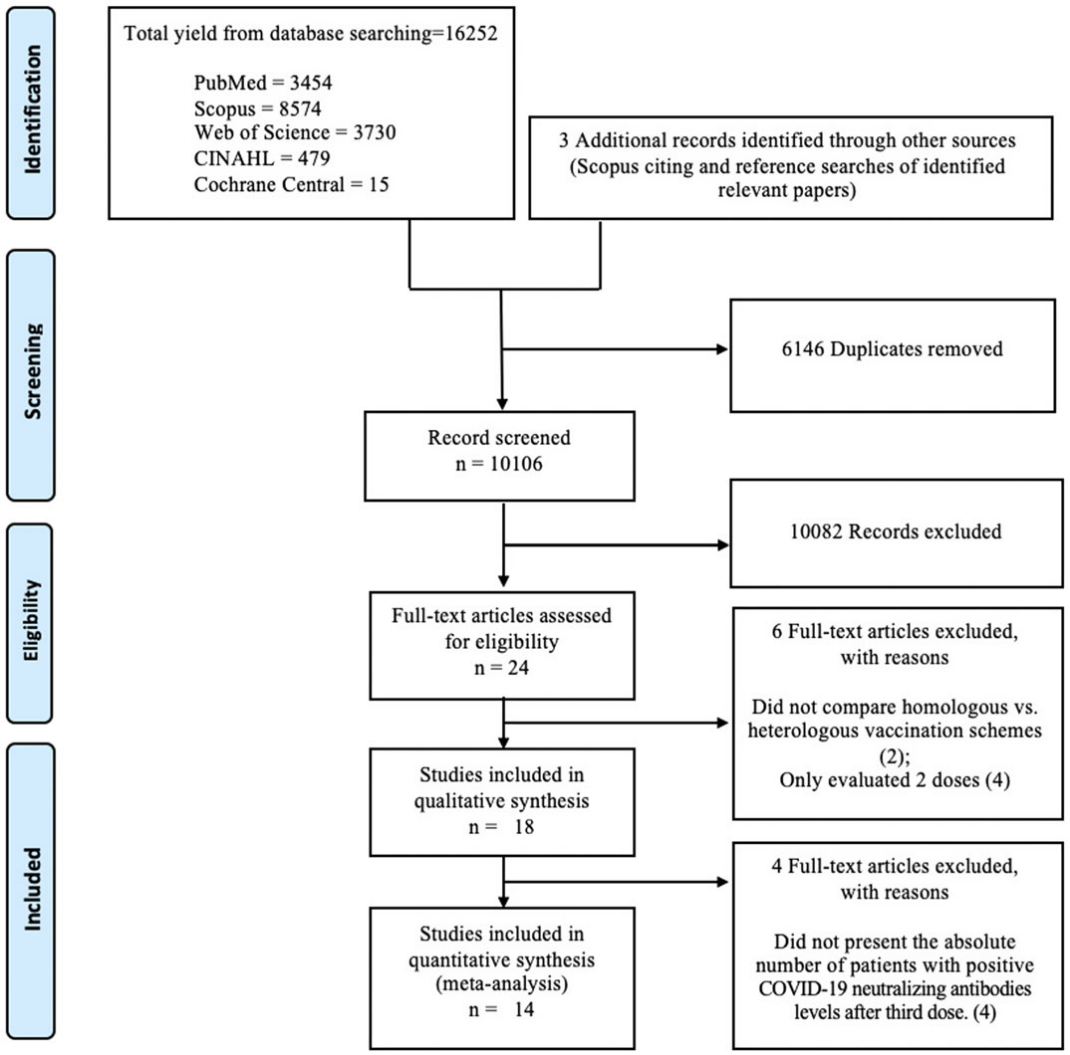
Literature search for articles on COVID-19 vaccine effectiveness among immunocompromised individuals.

Eighteen studies met the inclusion criteria^17–34^ and were included in the systematic literature review (Figure 2 and Table 1). Five were randomized clinical trials,^17,18,26,31,33^ four were retrospective cohort studies,^19,20,25,34^ and nine were prospective cohort studies.^21–24,27–30,32^ Most studies were conducted in Austria (four studies),^18,26,31,33^ and in the United Stated of America (four studies),^19,20,25,34^ followed by Chile (two studies),^21,24^ and Iran (one study),^17^ Turkey (one study),^22^ United Kingdom (one study),^23^ Hungary (one study),^23^ Korea (one study),^28^ Germany (one study),^29^ Brazil (one study),^30^ and Taiwan (one study).^32^ Studies were performed between March 2021 and January 2023,^17–34^ varying from 1 month to 10 months.

**Table 1.** Summary of characteristics of studies included in the systematic literature review

First author, year, location Study design Study period in # of months and [dates]	Participants (n) and immunosuppressive characteristics	COVID-19 vaccine schemes studied (n of individuals each group)	Anti-SARS-Cov-2 spike protein IgG neutralizing antibodies’ analysis	Total or % participants with Anti-SARS-Cov-2 spike protein IgG [heterologous versus homologous controls]		Mean (SD) or median [IQR] antibody titers		Total or % with COVID-19		Other COVID-19 Outcomes Included (number of cases)	Benefit of COVID-19 heterologous vaccines to enhance vaccine effectiveness in comparison to homologous vaccines	D&B Score (max = 28)
				After 2^nd^ dose	After 3^rd^ dose	Heterologous vaccines	Control group [Homologous vaccine]	Heterologous vaccines	Control group [Homologous vaccine]			
Aliabadi, 2022, Tehran – Iran RCT 6 months (July 2022–January 2023)	61 participants as auto-hematopoietic stem cell transplant recipients	2 doses of PastoCovac + 1 dose of CoronaVac (31) 3 doses of PastoCovac (30)	Yes	90%versus 90.2%	100% versus 93%	Mean Immune Status Ratio: 5.12	Mean Immune Status Ratio: 3.42	2	4	Hospitalization (1)	Yes. Heterologous boosting with an inactivated platform yielded a superior serologic response and non-significantly more reactogenicity than homologous RBD-TT conjugated boosting.	27
Bonelli, 2022, Vienna – Austria RCT 2, 5 months (May 26, 2021–August 5 2021)	55 participants with chronic inflammatory rheumatic (49) or neurologic diseases (6) under rituximab therapy	2 doses of Pfizer/BioNTech or Moderna + 1 AstraZeneca (27) 3 doses of Pfizer/BioNTech or Moderna (28)	No	0% versus 0%	22% versus 32%	19.4 [IQR: 8.2, 114.8] BAU/mL	12.4 [IQR: 3.8, 17.8] BAU/mL	NR	NR	NR	No. No significant advantage for either the homologous or heterologous vaccination strategy was found.	27
Chang, 2022, Baltimore – United States Retrospective Cohort Study 1 month	97 participants as various solid organ transplant recipients	2 doses of Pfizer/BioNTech + 1 dose of Moderna (14) 3 Pfizer (83)	Yes	0% versus 0%	50% versus 19%	NR	NR	NR	NR	NR	Yes. Mixing mRNA vaccine platforms could improve immunogenicity due to differences in antibody effector functions.	16
Chiang, 2022, Baltimore – United States Retrospective Cohort Study 10 months (March 2021–January 2022)	377 participants as various solid organ transplant recipients	2 doses of Pfizer/BioNTech + 1 dose of Janssen (21) 2 doses of Moderna + 1 dose of Janssen (19) 3 doses of Pfizer/BioNTech (220) 3 doses of Moderna (117)	No	0% versus 0%	63% versus 52%	NR	NR	1	35	NR	Yes. Heterologous vaccination with Janssen vaccine was associated with higher late seroconversion than homologous vaccination.	18
Dib, 2022, Santiago – Chile Prospective Cohort Study 5 months (October 2021–February 2022)	140 participants as various solid organ transplant recipients	2 doses of CoronaVac + 1 dose of Pfizer/BioNTech (78) 3 doses of Pfizer/BioNTech (62)	Yes	NR	55.1% versus 77.42%	58.7 RU/ml*	30.9 RU/ml*	NR	NR	NR	No. Homologous mRNA vaccine priming and boosting reaches a higher specific humoral immune response than inactivated SARS-CoV-2 vaccine priming followed by an mRNA vaccine booster.	23
Erol, 2023, Ankara – Turkey Prospective Cohort Study NR	95 participants as solid organ transplant recipients (62; 44 liver, 18 kidney) or HSCT (27; 5 allogeneic, 22 autologous)	2 doses of CoronaVac + 1 dose of Pfizer/BioNTech (70) 3 doses of CoronaVac (25)	Yes	NR	NR	26.76 (SOT) * 91.29 (HSCT) *	10.89 (SOT) * 34.82 (HSCT) *	NR	NR	NR	Yes. This study highlights the superiority of Pfizer/BioNTech responses as the third dose when compared with CoronaVac responses after two doses of CoronaVac.	16
Fendler, 2022, London – UK Prospective Cohort Study NR	199 participants with hematological (44) or solid (155) malignancies	2 doses of AstraZeneca + 1 dose of Pfizer/BioNTech (134) 3 doses of Pfizer/BioNTech (65)	Yes	63% versus 37%	NR	NR	NR	NR	NR	NR	Yes. Neutralizing antibodies titers were higher in patients who received a heterologous vaccination scheme.	21
Gaete-Argel, 2023, Santiago – Chile Prospective Cohort Study NR	45 participants as various solid organ transplant recipients	2 doses of CoronaVac + 1 dose of Pfizer/BioNTech (9) 3 doses of Pfizer/BioNTech (10)	Yes	28.6 versus 50%	NR	NR	NR	NR	NR	NR	No. We detected an important increase in cumulative seroconversion rates, especially after the second booster under a homologous scheme.	15
Greenberger, 2021, United States Retrospective Cohort Study 2 months [April 2021–June 2021]	24 participants with hematological malignancies-chronic lymphocytic leukemia (11); non-Hodgkin’s lymphoma (7); Waldenstrom’s macroglobulinemia (5); multiple myeloma (1).	2 doses of Pfizer/BioNTech + 1 dose of AstraZeneca (12) 2 doses of Pfizer/BioNTech + 1 dose of Moderna (3) 2 doses of Moderna + 1 dose of AstraZeneca (3) 2 doses of Moderna + 1 dose of Pfizer/BioNTech (1) 3 doses of Pfizer/BioNTech (2) 3 doses of Moderna (3)	No	26% versus 0%	57.9% versus 60%	NR	NR	NR	NR	NR	No. There was no evident pattern of antibody response among patients who received homologous versus heterologous vaccine	10
Heinzel, 2022, Vienna – Austria RCT 5 months (August 3 2021–December 31 2021)	169 participants kidney transplant recipients (KTR) with immunosuppressive medication following transplantation.	2 doses of Pfizer/BioNTech + 1 dose of Janssen (84) 3 doses of Pfizer/BioNTech (85)	No	0% versus 0%	50% versus 45%	NR	NR	4	3	Death (2) Hospitalization (3)	Yes. Heterologous 3rd dose using Janssen vaccine results in significantly higher antibody levels in KTR over a 3-month follow-up period compared to homologous vaccination	21
Honfi, 2022, Szeged – Hungary Prospective Cohort Study 6 months (October 2021–March 2022)	46 participants with Autoimmune and Inflammatory Rheumatic and Musculoskeletal Disease (aiRMDs)	2 doses of AstraZeneca + 1 dose of Pfizer/BioNTech (2) 2 doses of CoronaVac + 1 dose of Pfizer/BioNTech (4) 2 doses of Sputnik + 1 dose of Pfizer/BioNTech (6) 3 doses of Pfizer/BioNTech (22)	Yes	NR	100% versus 95.5%	1689 [631–3162] BAU/mL	1553 [276–3211] BAU/mL	0	0	NR	No. The third booster mRNA-based vaccine was similarly effective both in the homologous and heterologous groups compared to the infection-boosted patients	16
Kang, 2022, Seoul – Korea Prospective Cohort Study 6 months (October 2021–March 2022)	148 participants as various solid organ transplant recipients	2 doses of AstraZeneca + 1 dose of Pfizer/BioNTech (36) 2 doses of AstraZeneca + 1 dose of Moderna (29) 1 dose of AstraZeneca + 2 doses of Pfizer/BioNTech (45) 3 doses of Pfizer/BioNTech (33) 3 doses of Moderna (3) 2 doses of Moderna + 1 dose of Pfizer/BioNTech (1) 1 dose of Janssen + 1 dose of Moderna (1)	Yes	NR	72.7% versus 81.8%	NR	NR	NR	NR	NR	No. Third-dose mRNA vaccine-based heterologous vaccinations showed comparable humoral immunogenicity with homologous schemes.	18
Korber, 2023, Munich – Germany Prospective Cohort Study NR	26 participants as kidney transplant recipients.	1 dose of AstraZeneca + 2 doses of Pfizer/BioNTech/Moderna (8) 3 Pfizer (18)	Yes	62.5% versus 33.3%	87.5% versus 50%	NR	NR	3	7	NR	Yes. SARS-CoV-2-specific NAb titers were comparable between homologous and heterologous schemes, whereas NAb positivity rates were significantly higher in heterologous group upon third vaccination.	19
Medina-Pestana, 2022, São Paulo – Brazil Prospective Cohort Study NR	1084 participants as kidney transplant recipients.	2 doses of CoronaVac + 1 doses of Pfizer/BioNTech (307) 3 doses of CoronaVac (777)	Yes	36.2 % versus 34.5%	67.4% versus 55.5%	7,771 [1295–20 158] AU/mL	599 [195–1661] AU/mL	5	26	Death (8)	Yes. In kidney transplant recipients initiated with 2 doses of an inactivated vaccine, combining different vaccine platforms elicited a stronger humoral immune response, compared to administering a homologous booster dose.	21
Mrak, 2022, Vienna, Austria RCT 4 months (July 2021–October 2021)	51 participants under immunosuppressive therapy	2 doses of Pfizer/BioNTech/Moderna + 1 dose of AstraZeneca (25) 3 doses of Pfizer/BioNTech/Moderna (26)	No	0% versus 0%	18% versus 63%	NR	NR	NR	NR	NR	No. The seroconversion rate was significantly higher in the mRNA (homologous) than in the vector-vaccinated (heterologous) group.	26
Narongkiatikhun, 2023, Chiang Mai – Taiwan Prospective Cohort Study 7 months (June 1, 2021–December 31, 2021)	130 participants in maintenance hemodialysis.	1 dose of CoronaVac + 1 dose of AstraZeneca (25) 2 doses of CoronaVac (16) 2 doses of AstraZeneca (89)	Yes	0% versus 0% (AZ) versus 0% (SV)	88% versus 78.7% (AZ) versus 68.8% (SV)	NR	NR	NR	NR	NR	Yes. Prescribing different vaccine platforms seemed to be more efficacious in terms of inducing vaccine immunogenicity.	21
Reindl-Schwaighofer, 2021, Vienna, Austria RCT 3 months (June 2021–August 2021)	197 participants as kidney transplant recipients.	2 doses of Pfizer/BioNTech/Moderna + 1 dose of Janssen (98) 3 doses of Pfizer/BioNTech/Moderna (99)	No	0% versus 0%	42% versus 35%	NR	NR	NR	NR	NR	No. Homologous and heterologous vaccination strategies for a third SARS-CoV-2 vaccine dose in kidney transplant recipients are comparable.	25
Thompson, 2023, Baltimore – Maryland Retrospective Cohort Study NR	75 participants as various solid organ transplant recipients	2 doses of Pfizer/BioNTech/Moderna + 1 dose of Janssen (40) 3 doses of Pfizer/BioNTech/Moderna (35)	Yes	NR	NR	NR	NR	NR	NR	NR	No. Evidence of both quantitative and qualitative responses are induced by homologous mRNA versus heterologous Janssen boosting.	15

Abbreviations: AU/mL Arbitrary Units per milliliter, AZ, AstraZeneca; BAU/mL, Binding Antibody Units per milliliter; D&B, Downs and Black; HSCT, Hematopoietic Stem Cell Transplantation; IQR, Interquartile Range; KTR, Kidney transplant recipients; Nab, Neutralizing Antibodies; NR, Not Reported; RCT, Randomized Clinical Trial; SD, Standard Deviation; SOT, Solid Organ Transplant; SV, CoronaVac; UK, United Kingdom.

*GMC = geometric mean concentration.

In our qualitative analysis, eighteen studies including 3,019 individuals evaluated the effect of a heterologous vaccination strategy on immunocompromised individuals using outcomes related to COVID-19 (levels of anti-SARS-CoV-2 spike protein IgG, neutralizing antibodies, COVID-19, hospitalization, and death) in comparison to homologous strategies. Of the eighteen studies evaluated, sixteen evaluated Pfizer/BioNTech mRNA COVID-19 vaccine.^18–31,33,34^ Nine of these studies also evaluated Moderna mRNA COVID-19 vaccine,^18–20,25,28,29,31,33,34^ seven studies also assessed AstraZeneca COVID-19 vaccine,^18,23,25,27–29,31^ five studies analyzed Janssen COVID-19 vaccine,^20,26,28,33,34^ five studies assessed CoronaVac COVID-19 vaccine,^21,22,24,30,32^ and one study evaluated Sputinik COVID-19 vaccine.^27^ There were two additional studies: one study compared the PastoCovac (also called Soberana 02, manufactured in the Pasteur Institute of Iran in collaboration with the Finlay Vaccine Institute of Cuba)^17^ and CoronaVac COVID-19 vaccines.^32^ Twelve studies evaluated transplant recipients,^17,19–22,24,26,28–30,33,34^ being ten studies of solid organ transplants,^19–21,24,26,28–30,33,34^ one study of hematopoietic stem cell transplant,^17^ and one study evaluated both types of transplants (solid and hematopoietic stem cell transplant).^22^ Also, two studies evaluated individuals under immunosuppressive therapy,^18,31^ one study reviewed individuals with autoimmune and inflammatory rheumatic and musculoskeletal disease,^27^ two studies investigated individuals with malignancies,^23,25^ and one study analyzed patients undergoing maintenance hemodialysis.^32^

Studies showed significant variations in the reporting of serological test characteristics. There was limited consensus on the time of performance after the third dose, cutoff levels for antibody positivity, and the specific type of serological test conducted (Supplemental Table 1). Among the eighteen studies included in the systematic literature review, one study did not provide information on when the serological test was performed.^23^ Additionally, nine studies did not report any investigation into cellular immunity,^17,19,20,22,24,25,28,30,32^ while the remaining nine studies that conducted this analysis employed different approaches to assess cellular immunity.^18,21,23,26,27,29,31,33,34^ (Supplemental Table 1).

Regarding the quality assessment scores, ten studies were considered good (>18 of 28 possible points) per the Downs and Black quality tool,^17,18,21,23,26,29–33^ seven studies were considered fair (15–18 points),^19,20,22,24,27,28,34^ and one study was considered poor quality (<14 points).^25^

### Outcomes measures

**Figure 3.**
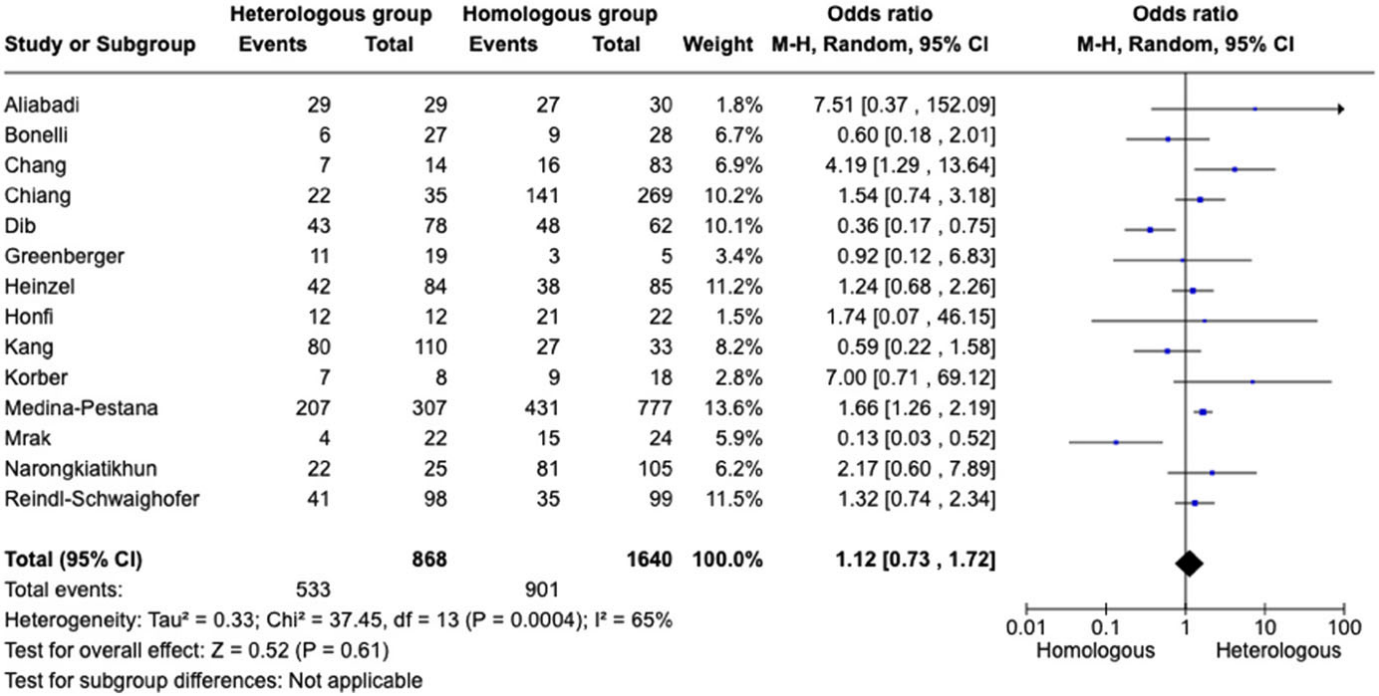
Forest plot of COVID-19 vaccine response (anti-SARS-CoV-2 spike protein IgG) after three doses of COVID-19 vaccine [n = 14 studies] with heterologous and homologous vaccination schemes. Odds ratios (OR) were determined with the Mantel–Haenszel random-effects method. Abbreviations: CI, confidence interval; M-H, Mantel–Haenszel.

Overall, fourteen studies,^17–21,25–33^ including 2,508 immunocompromised individuals evaluated the antibody response (anti-SARS-CoV-2 spike protein IgG) and were included in the meta-analysis. The positive antibody response rate in 2,508 immunocompromised individuals ranged from 18% to 100%. In total, 61.4% of individuals had positive antibody response in the heterologous vaccination group, while 54.9% had positive antibody response in the homologous vaccination group. The heterologous vaccination group had no difference in the odds of developing anti-SARS-CoV-2 spike protein IgG compared to the homologous vaccination (pooled odds ratio 1.12 [95% Cl: 0.73–1.72] (Figure 3). From the fourteen studies included in the meta-analysis, eight studies^17,19,21,27–30,32^ also analyzed anti-SARS-Cov-2 spike protein IgG neutralizing antibodies. In the stratified analysis, the pooled odds ratio for developing neutralizing antibodies among the heterologous group was 1.48 [95% Cl: 0.72–3.05] compared to homologous strategies (Supplemental Table 2). Regarding the heterologous vaccination response among different immunocompromising conditions, 100% of hematological transplant recipients^17^ and autoimmune and inflammatory rheumatic and musculoskeletal individuals,^27^ 88% of individuals undergoing maintenance hemodialysis,^32^ 62.6% of solid organ transplant recipients,^19–21,28–30,33^ 57.9% of individuals with malignant tumor,^25^ and 20.4% of individuals on immunosuppressive therapy^18,26^ had a positive antibody response (Supplemental Table 2).

**Figure 4.**
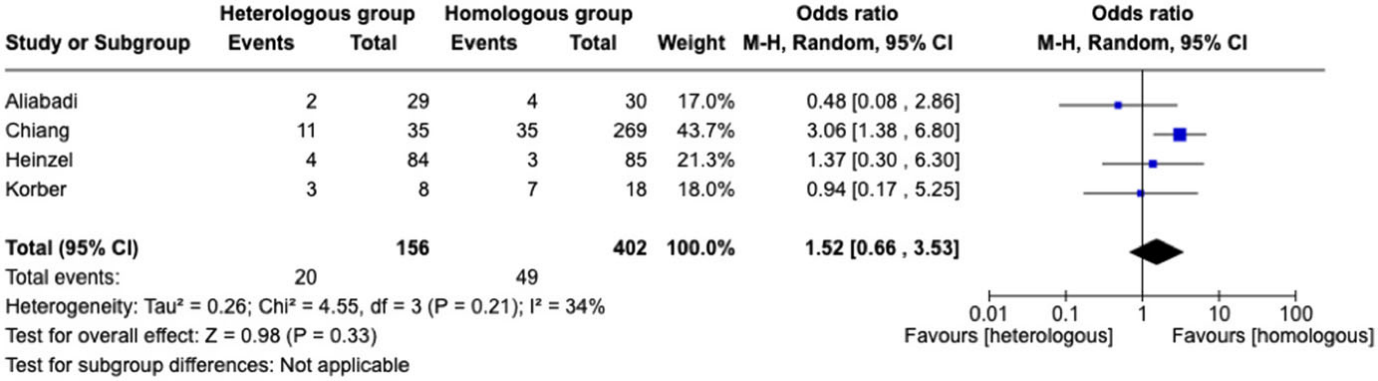
Forest plot of COVID-19 after three doses of COVID-19 vaccine [n = 4 studies] with heterologous and homologous vaccination schemes. Odds ratios (OR) were determined with the Mantel–Haenszel random-effects method. Abbreviations: CI, confidence interval; M-H, Mantel–Haenszel.

Four studies,^17,20,26,29^ with a total of 558 immunocompromised individuals, also evaluated symptomatic COVID-19 (Figure 4). In a group of 156 individuals in the heterologous vaccination, 12.8% developed COVID-19, while in the homologous vaccination group of 402 individuals, 12.2% developed COVID-19. The pooled odds ratio to acquire COVID-19 in the heterologous vaccination group was 1.52 [95% CI: 0.66–3.53] compared to the homologous strategy (Supplemental Table 2).

Among eighteen studies, three^17,26,30^ reported other COVID-19 outcomes, including hospitalizations and deaths related to COVID-19. In total, hospitalizations were seen in 0.30% (4/1,314) of patients, including 0.71% (3/422) in the heterologous strategy and 0.11% (1/892) in the homologous strategy. Deaths were observed in 0.76% (10/1,314) of patients, including 0.95% (4/422) in the heterologous strategy and 0.67% (6/892) in the homologous strategy among the three studies^17,26,30^

The results of meta-analyses represented substantial heterogeneity for studies evaluating anti-SARS-Cov-2 spike protein IgG in individuals who received the COVID-19 vaccine heterologous or homologous scheme (heterogeneity *P* = 0.61, I^2^ = 65%), and homogenous for studies evaluating VE on COVID-19 in individuals who received the COVID-19 vaccine heterologous or homologous scheme (heterogeneity *P* = 0.21, I^2^ = 34%), respectively.

### Publication bias

We conducted a publication bias analysis through funnel plot visualization of studies evaluating COVID-19 vaccine response with anti-SARS-CoV-2 spike protein IgG and studies evaluating COVID-19 (Supplemental Figure 1A and 1B). In both graphs, the studies were reasonably balanced around the pooled ORs with little evidence of publication bias. The Egger’s test also did not indicate publication bias among those included studies in the meta-analysis (*P* = 0.55).

## Discussion

This systematic literature review and meta-analysis demonstrate that heterologous COVID-19 strategies result in comparable antibody responses to homologous strategies among immunocompromised patients. Moreover, effectiveness was found to be similar between those with heterologous and homologous booster strategies. Despite moderate heterogeneities, these findings support the flexibility of using vaccines with different vectors and delivery systems, considering supply and logistical factors.

As new variants of COVID-19 continue to emerge, the COVID-19 vaccination program aims to prevent severe disease through the administration of new booster shots. For example, the JN.1 lineage became predominant in United States in January 2024,^35^ The World Health Organization (WHO) has classified this variant as a Variant of Interest (VOI) due to its rapidly increasing spread.^36^ The emergence of this VOI and other variants, which can spread easily even among individuals who have had a previous infection or vaccination, raises the risk of reinfection or breakthrough cases. This is particularly concerning for people with weakened immune systems, as the virus can persist for longer periods, increasing the likelihood of generating new variants that may be more challenging to manage.^37,38^ Additionally, the prevalence of immunosuppressed individuals has increased from 2.7% in 2013 to 6.3% in 2021, further highlighting the urgency of addressing the unique challenges faced by this population.^14^ There is a pressing need for studies assessing the effectiveness of vaccines against new variants specifically in the immunosuppressed population. In the meta-analysis, all studies measured the vaccine response using anti-SARS-CoV-2 spike protein IgG. However, a limited number of studies evaluated the neutralizing ability against the virus, directly measuring the capacity to inhibit viral replication. Further research is essential in the immunosuppressed population to distinguish between antibody production and actual protection against symptomatic COVID-19. Additionally, it is crucial to investigate the durability of this protection. Moreover, there is a need for specific strategies tailored to individuals with severe immunosuppression compared to those with milder degrees of immunosuppression. Having target recommendations for different levels of immunosuppression would enhance the precision and effectiveness of our guidance.

The demonstrated similarity in antibody response and effectiveness between heterologous and homologous vaccination strategies highlights the adaptability and potential efficacy of diverse vaccine regimens. Although the CDC allows adults to receive a different manufacturer booster from the type of the primary series, children aged less than 4 years are still recommended to receive homologous boosters.^39^ A number of low- and middle-income countries still do not have adequate accessibility of COVID-19 vaccines and have high unmet demand.^40,41^ Adopting heterologous booster strategies could be valuable for reinforcing the immune response in immunocompromised individuals amid emerging COVID-19 variants. Tailoring vaccination approaches for this vulnerable population is crucial, and our study provides empirical support for considering alternative schedules. These insights can guide the development of evidence-based recommendations, assisting policymakers in more effectively allocating resources and optimizing vaccine distribution. Our study advocates for ongoing vigilance in the face of evolving variants, emphasizing the need for continuous monitoring and adaptive public measures for sustained protection of immunocompromised individuals against severe outcomes of COVID-19.

This study has several limitations. First, most studies included were non-randomized (13 of 18), which introduces potential sources of bias in our findings. Non-randomized designs may be influenced by confounding variables, limiting the ability to establish causal relationships with confidence. This aspect underscores the need for caution in drawing definitive conclusions about the comparative effectiveness of heterologous and homologous vaccination. Furthermore, the diverse array of serological tests adopted across the studies, each with different cutoff levels for antibody positivity, poses a significant challenge. This heterogeneity could introduce variability in the interpretation of antibody response rates. Secondly, the study’s focus on the measurement of vaccine response primarily through anti-SARS-CoV-2 spike protein IgG, while informative, may not provide a comprehensive understanding of the overall immune response. The exclusive reliance on IgG levels, without a thorough examination of other antibody types or neutralizing capacity, limits the depth of our insights into the true protective efficacy of the vaccines. We agree with the FDA guidance cautioning against using antibody testing as a sole indicator of immunity, emphasizing the need for a more nuanced understanding of the relationship between serological markers and protection against symptomatic COVID-19. As such, this study calls for future research to delve into these complexities and broaden the scope of assessment for a more holistic evaluation of vaccine effectiveness in immunocompromised individuals. Thirdly, we lack substantial data on the vaccine’s effectiveness in preventing severe disease or mortality among immunocompromised populations. Among all the studies reviewed, only four reported symptomatic COVID-19, while three outlined other outcomes (hospitalization and death). There was no data on these four included studies about the unvaccinated individuals for vaccine effectiveness. Fourth, we considered the Janssen dose as equivalent to two doses of other vaccines. In four studies,^20,26,33,34^ the Janssen booster dose was evaluated after two doses of mRNA vaccines, which would total four doses in our classification. However, this situation accounts for approximately only 1% of all patients included. Hence, it did not have a big influence on our results focusing on three COVID-19 doses. Fifth, given the immunocompromised nature of the study population, addressing the potential impact of underlying comorbidities on vaccine response and effectiveness might be relevant. Certain comorbidity conditions could influence the outcomes and should be acknowledged as potential confounders. Lastly, our systematic literature review did not specifically address the comparison of different COVID-19 variants or the role of repeated infections, which could potentially contribute to variations in infection and mortality rates. This review tries to contribute to the broader discussion on equitable access to effective vaccination, particularly for vulnerable populations.

In conclusion, heterologous COVID-19 vaccines have demonstrated comparable rates of antibody response and effectiveness compared to homologous strategies in immunocompromised individuals. This approach could potentially help address global disparities in vaccine distribution. More studies are necessary to evaluate vaccine effectiveness for different vaccination strategies, VE against new variants, and the clinical significance of anti-SARS-CoV-2 spike protein IgG antibody levels in immunosuppressed populations, the most vulnerable to severe COVID-19 disease.

## Supporting information

Pardo et al. supplementary material Pardo et al. supplementary material

## Supplementary material

For supplementary material accompanying this paper visit http://doi.org/10.1017/ash.2024.369. click here to view supplementary material
