# Supplementary material for: Effectiveness of heterologous and homologous COVID-19 vaccination among immunocompromised individuals: a systematic literature review and meta-analysis
Source: Antimicrob Steward Healthc Epidemiol. 2024 Sep 26;4(1):e152. doi: 10.1017/ash.2024.369 (PMC11427957; doi:10.1017/ash.2024.369)
Supplement: Pardo et al. supplementary material [file S2732494X24003693sup001.docx]

**Supplementary Appendix**

**Supplemental Table 1.** Summary of characteristics of humoral and cellular immunity investigation on the included studies in the systematic literature review

| **First author, year, location** | **Serological kit performed** | **When the serological test was performed** | **Cutoff level for antibody positivity** | **Cellular immunity investigation, and method** |
| --- | --- | --- | --- | --- |
|  |  |  |  |  |
| Aliabadi, 2022,  Tehran – Iran | Indirect-ELISA ChemoBind SARS-CoV-2 Neutralizing Antibody Test Kit (ChemoBind) to measure vaccine-induced antibody responses against the SARS-CoV-2 spike protein (anti-S). | 4 weeks after third dose. | IgG Immune status ratio >1.1 | No. |
| Bonelli, 2022, Vienna - Austria | RBD: Elecsys Anti-SARS-CoV-2 S immunoassay was used for the quantitative determination of antibodies to the receptor-binding domain (RBD) of the viral spike (S) protein.  NT: Two-fold serial dilutions of heat-inactivated serum samples were incubated with 50–100 TCID50 SARS-CoV-2 for 1h at 37°C before the mixture was added to Vero E6 (ATCC ® CRL-1586) cell monolayers. | 4 weeks after third dose. | RBD >0.8 BAU/mL  NT titers ≥10 were considered positive. | Yes. T cell responses were determined by enzyme-linked immune absorbent spot (ELISpot) assay. |
| Chang, 2022, Baltimore – USA | Roche Elecsys Anti-SARS-CoV-2 S enzyme immunoassay. | 1 month after third dose. | Antibody level of ≥250 U/mL | No. |
| Chiang, 2022, Baltimore – USA | UROIMMUN EIA, testing for S1 domain of the SARS-CoV-2 spike protein (anti-S1, range 0.1, >8.94 arbitrary units. | 1 month after third dose. | Seropositive: ≥1.1 AU | No. |
| Dib, 2022, Santiago – Chile | SARS-CoV-2 Surrogate Virus Neutralisation Test (sVNT) Kit (Cat. L00847, Gen- Script, New Jersey, USA). Serum samples are diluted to a ratio of 1:10; the test assesses the presence/absence of NAb and allows the interpretation of the inhibition rate as = [1 −(OD450nm value of Sample/OD450nm value of Negative Control)] x 100%. | 8- 12 weeks following the third dose. | A percentage of neutralization ≥30 at a 1:10 sample dilution | Yes. Specific anti-SARS-CoV-2 T-cell response was assessed by a commercial human IFN-g /IL-2 double-color enzymatic ELISPOT assay (#hIFNgIL2, Cellular Technology Limited, OH, USA). |
| Erol, 2023, Ankara – Turkey | Plaque neutralization assays were conducted under BSL-3 conditions with Vero E6 cells cultured with DMEM High-Glucose. | 3-7 months after third dose. | GMT > 10 | No. |
| Fendler, 2022, London - UK | High-throughput live virus microneutralization assays were performed. Neutralizing antibody titers are reported as the fold-dilution of serum required to inhibit 50% of viral replication (IC50). | NR | IC50 titers >40 | T cell responses were measured using IFN-y ELISPOT. Responses are defined as IFN-y spot-forming units per 106 PBMC. |
| Gaete-Argel, 2023, Santiago – Chile | Quantification of anti-N antibodies was performed as described in by using the Electrochemiluminescent immunoassay (ECLIA) (Cobas, Roche). | 36 days after third vaccination. | anti-N antibodies ≥ 1.0 | No. |
| Greenberger, 2021, New York - USA | Roche Elecsys assay | 12-61 days after third vaccination. | Anti-Spike ab >0.4 | No. |
| Heinzel, 2022, Austria | Roche Elecsys anti–SARS-CoV-2 S enzyme immunoassay detecting antibodies against the receptor-binding domain of the SARS-CoV-2 spike protein. | One month post third dose;  Three months post third dose. | > 0,8 U/ml, > 15 U/ml, 100 U/ml, > 141 BAU/ml, and > 264 BAU/ml | Yes. T-cell stimulation flow cytometric (FC) assessment of SARS-CoV2-specific T-cells. Considered positive when the number of identified cells in the stimulated sample exceeded the number of such cells in the unstimulated sample by at least 2-fold. |
| Honfi, 2022, Szeged - Hungary | Siemens Advia Centaur XPT system using the Siemens Healthineers SARS-CoV-2 IgG assay (sCOVG) (Siemens Healthineers, Munich, Germany). | 4 months after third vaccination. | > 21.8 BAU/mL | Measurement of SARS-CoV-2 Specific T-Cell Memory was performed according to the instruction of the manufacturer using the SARS- CoV-2 Prot_S T Cell Analysis Kit (PBMC) (Miltenyi Biotec). Reactive cell numbers are shown in relation to 106 CD4+ or CD8+ T-cells, the cut-off value was 400 reactive cells of 106 parental population. |
| Kang, 2022, Seoul – Korea | Surrogate neutralizing antibody assay (sNAA) using the cPass SARS-CoV-2 Neutralization Antibody Detection kit (GenScript Inc., Piscataway, NJ, USA; protection cut-off < 30%) were performed according to the manufacturer’s instructions. | 14 to 150 days after third vaccination. | protection cut-off > 30% | No. |
| Korber, 2023, Munich – Germany | Surrogate neutralization was quantified using the commercial iFlash-2019-nCoV NAb kit. SARS-CoV-2-specific NAb in patients’ sera form a complex with SARS-CoV-2 RBD antigen-coated paramagnetic microparticles. Acridinium-ester-labeled ACE2 conjugates competitively bind the remaining RBD. | 13 to 36 days after third vaccination. | Values ≥ 10 AU/ ml | Cell counting was performed on an ImmunoSpot Ultimate UV Image analyzer (CTL Europe GmbH). |
| Medina-Pestana, 2022, São Paulo – Brazil | cPass SARS-CoV-2 Neutralization Antibody Detection Kit, a test based on antibody-mediated blockage of the interaction between the angiotensin- converting enzyme 2 receptor protein and the RBD in the 2 laboratory evaluations. | 4 weeks after third vaccination. | ≥30% signal inhibition | No. |
| Mrak, 2022, Vienna, Austria | Quantification of antibodies to the receptor-binding domain (RBD) of the viral spike (S) protein was performed using the Elecsys Anti-SARS-CoV-2 S immunoassay. Analysis was performed on a Cobas e801 device (Roche Diagnostics, Rotkreuz, Switzerland). | 4 weeks after third vaccination. | Antibody concentrations > 0.8 BAU/mL | SARS-CoV-2 spike-specific T-cell responses were determined by ELI- Spot assay before and after booster vaccination. |
| Narongkiatikhun, 2023, Chiang Mai -Taiwan | *Anti-RBD IgG Assay*: Abbott SARS-CoV-2 IgG II Quantification assay -chemiluminescent microparticle immunoassay (CMIA) used for the quantitative determination of IgG antibodies to the SARS-CoV-2 spike protein.  *sVNT:* SARS-CoV-2 NeutraLISA surrogate neutralization assay for inhibitory ability of the binding of RBD of viral SARS-CoV-2 S1 to the angiotensin-converting enzyme 2 receptor. | 28 days post second dose. | *Anti-RBD IgG Assay*: ≥7.1 BAU/mL  *sVNT:* ≥35% inhibition | No. |
| Reindl-Schwaighofer, 2021, Vienna, Austria | Elecsys^®^ Anti-SARS-CoV-2 enzyme immunoassay (Roche Diagnostics), which tests for the receptor-binding domain of the SARS-CoV-2 spike protein. | 4 weeks after third vaccination. | ≥0.8 U/mL | QuantiFERON SARS-CoV-2 assays (Qiagen), a SARS-CoV-2 spike protein-specific interferon-γ release assay (IGRA) was used to assess T-cell response. Signals greater than 0.1 IU/mL were considered to be positive. |
| Thompson, 2023, Baltimore – Maryland | Anti-N, anti-RBD, and anti-S IgG were measured using the multiplex chemiluminescent MSD V-PLEX COVID-19 Respiratory Panel 3 Kit according to the manufacturer’s protocol at a dilution of 1:5,000. | 2 weeks after third vaccination. | “Positivity cutoff was determined by the manufacturer based on pre-pandemic serum samples and PCR-confirmed cases during the pre–SARS-CoV-2 vaccine period of the pandemic.” | Polyfunctional T cell responses were analyzed using Pestle v2.0 and SPICE v6.0(86). |

Ab=Antibody; AU=Arbitrary units per milliliter; BAU=Binding antibody unit; COI=Critical value index; ELISA=Enzyme-linked immunosorbent assay; KTR=Kidney transplant recipient; N=Nucleocapsid protein; NAb=Neutralizing antibody; NR=Not reported; PBMCs=Peripheral blood mononuclear cells; RBD=Receptor-binding domain; S=Spike protein; U/mL= units per milliliter; UK= United Kingdom; USA= United States of America; VE=Vaccine Effectiveness

**Supplemental Table 2.** Subset analyses evaluating the association between COVID-19 vaccine response (anti-SARS-CoV-2 spike protein IgG) after three doses of COVID-19 vaccine (14 studies)

| **Subset** | **Number of studies included** | **Pooled Odds Ratio, M-H, Random, comparing Heterologous with Homologous Groups (95% CI)** | **I**^2^ **test for heterogeneity** |
| --- | --- | --- | --- |
| Anti-SARS-CoV-2 Spike protein IgG | 14 | 1.12 (0.73 - 1.72) | 65% |
| Neutralizing antibodies * | 8 | 1.48 (0.72 – 3.05) | 71% |
| Solid organ transplant recipients | 7 | 1.27 (0.74 – 2.18) | 73% |
| Studies with 19–23 of 28 points (D&B, Good) | 9 | 1.01 (0.58 - 1.76) | 74% |
| Randomized Controlled Trials | 4 | 1.39 (0.40 – 4.81) | 74% |
| Infection** | 4 | 1.52 (0.66 - 3.53) | 34% |

CI=Confidence Interval; D&B=Downs & Black score; M-H=Mantel-Haenszel; Random=Random-effects method.

Some stratified analysis were not able to be conducted due to lack of studies.

* Measured in neutralizing antibodies.

** Measured in the number of COVID-19 post-COVID-19 vaccination

**Supplemental Figure 1A.** Funnel plot demonstrating the association between of COVID-19 vaccine response (antibody production) after three doses of COVID-19 vaccine with heterologous and homologous vaccination in immunocompromised. Abbreviations: SE, standard error; RR, relative risk


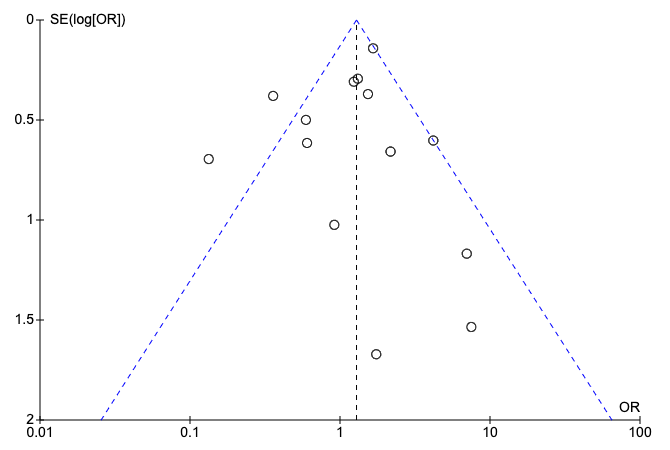


**Supplemental Figure 1B.** Funnel plot demonstrating the association between of COVID-19 after three doses of COVID-19 vaccine with heterologous and homologous vaccination in immunocompromised. Abbreviations: SE, standard error; RR, relative risk


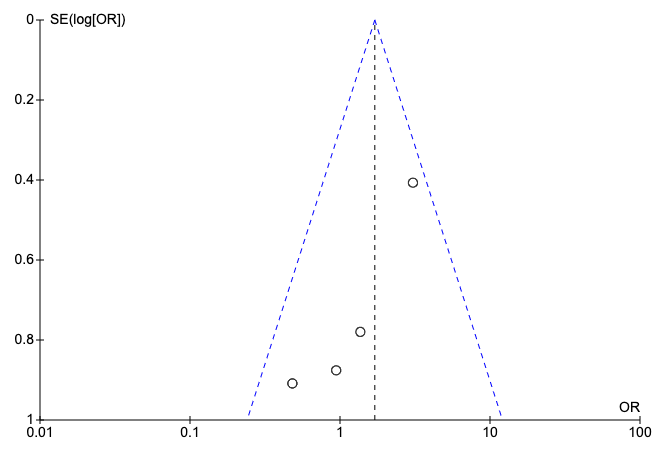


**Supplemental Form 1:** Data abstract form

**Effectiveness of heterologous and homologous COVID-19 vaccine among immunocompromised individuals: a systematic literature review and meta-analysis**

1. First author last name:_____________________________________________________
2. Publication year:__________________________________________________________
3. Study location (City, State, Country):__________________________________________
4. Reviewer’s initials:_________________________________________________________

**Part 1.**

Basic Inclusion/Exclusion Criteria:

1. Did the study evaluate **COVID-19 vaccine**? □ Yes □No (if no, exclude it)
2. Did the study evaluate **immunocompromised patients**? □ Yes □No (if no, exclude it)
3. Did the study evaluate **heterologous vaccination vs. homologous vaccination?** □ Yes □No (if no, exclude it)
4. If item #3 is yes, in which COVID-19 vaccine? □BNT162b2(Pfizer/BioNTech), or □ mRNA-1273 (Moderna), or □other (please circle –Ad26COVS1 (Janssen); ChAdOx1 nCoV-19 (AstraZeneca); BBIBP-CorV (Sinopharm vaccine); other COVID-19 vaccine
5. Did the study evaluate **vaccine response or vaccine effectiveness?**

□Yes □No (if no, exclude it)

1. Duration of study:___________(in weeks or months)
2. Do you believe this study should be excluded? □ Yes □No
3. If yes, why? _______________________________________________________________

**Part 2.**

Exposure and Outcomes Assessment (Check more than one if it is necessary)

1. Where did the study take place?

□ An academic medical center

□ A community hospital

□ A nursing homes

□ Other: ______________________________________

1. Which type is this study design?

□ Retrospective Cohort study

□ Prospective Cohort study

□ Case-control study

□ Randomized controlled trial

1. Was the study performed in more than one hospital? If yes please add the number of hospitals

□Yes (If yes, please add the # of hospitals)___ □ No

1. Which outcomes were considered in the study? □ COVID-19 □ Hospitalization □Death □Levels of neutralizing antibodies □ Other __________________
2. Which **COVID-19 tests** were used for diagnosing an initial COVID-19? (Check more than one if necessary)

□ RT-PCR □antigen-based test □other_________

1. What were the types of COVID-19 vaccines studied?
   1. □ mRNA vaccine □viral vector vaccine □inactivated virus vaccine
2. How did the study define a heterologous vaccination? _______________________________________________________________________________________________________________________________________________________
3. Did any of the individuals get **COVID-19 vaccine booster dose (3^rd^ dose or 4 ^rd^ dose)**? ____________________________________________________________________________________________________________________________________________________________
4. What were the heterologous vaccine schemes studied? (E.g. 2 CoronaVac + 1 Pfizer; 2 Pfizer + 1 Moderna)

_______________________________________________________________________________________________________________________________________________________

1. Which was the follow up time from last vaccine dose (in weeks or months)? ________________________
2. Did the study elucidate whether the vaccine response was specific for some variant? (eg: alpha, beta, gama, delta, etc) □ Yes (If Yes, which?_____________ )               □ No.
3. How the study calculated the vaccine effectiveness? □ 1 – odds ratio □ 1 – incidence ratio □ 1 – hazard ratio □ Other________________
4. Did the study evaluate adverse effects of the heterologous vaccination? □ Yes □No
5. How did the study define an **immunocompromised/immunosuppressed** person?

□ Medications: corticosteroids, chemotherapy or other immunosuppressive medications □ HIV

□ Solid organ transplant □ Hematopoietic stem cell transplant □ Thalassemia

□ Active cancer (current cancer or in treatment or received diagnosis within last 12 months)

□ Others: _______________________________________

1. Which kind of **statistical analysis** was performed? (Check all that apply)

□ Crude incidence rate ratio (IRR) and exact 95% confidence interval (CI)

□ Adjusted incidence rate ratio (IRR) and exact 95% confidence interval (CI)

□ Defined vaccine effectiveness (VE) as 1-IRR (rate ratio) analyzing fully vaccinated individuals

□ Defined vaccine effectiveness (VE) as 1-IR (relative risk)

□ Unadjusted relative risk (RR) and exact 95% confidence interval (CI)

□ Generalized linear regression model (Poisson distribution)

□ Cox proportional model

□ Adjusted logistic regression model

1. **Population characteristics** (Fill out **IF** only the information that are available on the papers)

|  | Heterologous Vaccine (3 doses) | Homologous Vaccine (3 doses) | Fourth Dose of a COVID-19 Vaccine |
| --- | --- | --- | --- |
| Vaccines Included |  |  |  |
| Total Number Participants |  |  |  |
| Age (Mean [SD] or Median [IQR]): |  |  |  |
| % of female |  |  |  |
| Total number of patients that had COVID-19 after 2^nd^ dose |  |  |  |
| Total number of patients that had COVID-19 after 3^rd^ dose |  |  |  |
| Total number of patients that had COVID-19 after 4^rd^ dose |  |  |  |
| Total individuals tested positive for SARS-CoV-2 |  |  |  |
| % of patients requiring hospitalization |  |  |  |
| % of patients requiring ICU |  |  |  |
| % of patients requiring mechanical ventilation |  |  |  |
| Duration of hospitalization (nº of days, mean ± SD or median) |  |  |  |
| Level of neutralizing antibodies |  |  |  |

**Part 3**. Unadjusted and adjusted associations

1. Raw numbers: Please fill raw data for the following tables if available. Only include measures of effect if they are listed in the manuscript

**Table A: Main Association of Interest**

|  | Outcome: COVID-19 | No outcome: non-COVID-19 |
| --- | --- | --- |
| Exposed: heterologous vaccine |  |  |
| Unexposed: homologous vaccine |  |  |

|  | Outcome: COVID-19 hospitalization | No outcome: non-COVID-19 hospitalization |
| --- | --- | --- |
| Exposed: heterologous vaccine |  |  |
| Unexposed: homologous vaccine |  |  |

|  | Outcome: COVID-19 death | No outcome: non-COVID-19 death |
| --- | --- | --- |
| Exposed: heterologous vaccine |  |  |
| Unexposed: homologous vaccine |  |  |

|  | Outcome: COVID-19 neutralizing antibodies levels | No outcome: non-COVID-19 neutralizing antibodies levels |
| --- | --- | --- |
| Exposed: heterologous vaccine |  |  |
| Unexposed: homologous vaccine |  |  |

**Incidence rate ratio (IRR) and vaccine effectiveness (VE) for each type of vaccination (heterologous/homologous)**

| **Heterologous Vaccination** | | **Homologous Vaccination** | |
| --- | --- | --- | --- |
| **Crude IRR (95% CI)** |  | **Crude IRR (95% CI)** |  |
| **Adjusted IRR (95% CI)** |  | **Adjusted IRR (95% CI)** |  |
| **VE (95% CI)** |  | **VE (95% CI)** |  |

*Adjusted covariates:____________________________________________________

Part 4. Other references

Please look through the references. Are there other references that we should evaluate for the meta-analysis? If yes, please provide first author, journal and year _______________________________________________________________________________________________________________________________________________________________________________________________________________________________________________________________

Part 5: Quality Assessment Tool:

Adapted Downs and Black Tool:

Is the hypothesis/aim/objective of the study clearly described?

| yes | 1 |
| --- | --- |
| no | 0 |

2. Are the main outcomes to be measured clearly described in the Introduction or Methods section?

If the main outcomes are first mentioned in the Results section, the question should be answered no.

| yes | 1 |
| --- | --- |
| no | 0 |
|  |  |

3. Are the characteristics of the participants include in the study clearly described?

In cohort and cross-sectional studies, inclusion and/or exclusion criteria should be given. In case-control studies, a case-definition and the sources for controls should be given.

| yes | 1 |
| --- | --- |
| no | 0 |

4. Are the interventions of interest clearly described?

Treatments and placebo (where relevant) that are to be compared should be clearly described.

| yes | 1 |
| --- | --- |
| no | 0 |
|  |  |

5. Are the distributions of principal confounders in each group of subjects to be compared clearly described? A list of principal confounders is provided.

| yes | 2 |
| --- | --- |
| partially | 1 |
| no | 0 |

6. Are the main findings of the study clearly described?

Simple outcome data (including denominators and numerators) should be reported for all major findings so that the reader can check the major analyses and conclusions. (This question does not cover statistical tests which are considered below).

| yes | 1 |
| --- | --- |
| no | 0 |

7. Does the study provide estimates of the random variability in the data for the main outcomes?

In non-normally distributed data the inter-quartile range of results should be reported. In normally distributed data the standard error, standard deviation or confidence intervals should be reported. If the distribution of the data is not described, it must be assumed that the estimates used were appropriate and the question should be answered yes.

| yes | 1 |
| --- | --- |
| no | 0 |

8. Have all important adverse events that may be a consequence of the intervention been reported?

This should be answered yes if the study demonstrates that there was a comprehensive attempt to measure adverse events. (A list of possible adverse events is provided).

| yes | 1 |
| --- | --- |
| no | 0 |

9. Have the characteristics of patients lost to follow-up been described?

This should be answered yes where there were no losses to follow-up or where losses to follow-up were so small that findings would be unaffected by their inclusion. This should be answered no where a study does not report the number of patients lost to follow-up.

| yes | 1 |
| --- | --- |
| no | 0 |

10. Have actual probability values been reported (e.g. 0.035 rather than <0.05) for the main outcomes except where the probability value is less than 0.001?

| yes | 1 |
| --- | --- |
| no | 0 |

External validity:

All the following criteria attempt to address the representativeness of the findings of the study and whether they may be generalized to the population from which the study subjects were derived.

11. Were the subjects asked to participate in the study representative of the entire population from which they were recruited?

The study must identify the source population for patients and describe how the patients were selected. Patients would be representative if they comprised the entire source population, an unselected sample of consecutive patients, or a random sample. Random sampling is only feasible where a list of all members of the relevant population exists. Where a study does not report the proportion of the source population from which the patients are derived, the question should be answered as unable to determine.

| yes | 1 |
| --- | --- |
| no | 0 |
| unable to determine | 0 |

12. Were those subjects who were prepared to participate representative of the entire population from which they were recruited?

The proportion of those asked who agreed should be stated. Validation that the sample was representative would include demonstrating that the distribution of the main confounding factors was the same in the study sample and the source population.

| yes | 1 |
| --- | --- |
| no | 0 |
| unable to determine | 0 |

13. Were the staff, places, and facilities where the patients were treated, representative of the treatment the majority of patients receive?

For the question to be answered yes the study should demonstrate that the intervention was representative of that in use in the source population. The question should be answered no if, for example, the intervention was undertaken in a specialist centre unrepresentative of the hospitals most of the source population would attend.

| yes | 1 |
| --- | --- |
| no | 0 |
| unable to determine | 0 |

Internal validity – bias

14. Was an attempt made to blind study subjects to the intervention they have received ?

For studies where the patients would have no way of knowing which intervention they received, this should be answered yes.

| yes | 1 |
| --- | --- |
| no | 0 |
| unable to determine | 0 |

15. Was an attempt made to blind those measuring the main outcomes of the intervention?

| yes | 1 |
| --- | --- |
| no | 0 |
| unable to determine | 0 |

16. If any of the results of the study were based on “data dredging”, was this made clear?

Any analyses that had not been planned at the outset of the study should be clearly indicated. If no retrospective unplanned subgroup analyses were reported, then answer yes.

| yes | 1 |
| --- | --- |
| no | 0 |
| unable to determine | 0 |

17. In trials and cohort studies, do the analyses adjust for different lengths of follow-up of patients, or in case-control studies, is the time period between the intervention and outcome the same for cases and controls ?

Where follow-up was the same for all study patients the answer should yes. If different lengths of follow-up were adjusted for by, for example, survival analysis the answer should be yes. Studies where differences in follow-up are ignored should be answered no.

| yes | 1 |
| --- | --- |
| no | 0 |
| unable to determine | 0 |

18. Were the statistical tests used to assess the main outcomes appropriate?

The statistical techniques used must be appropriate to the data. For example non- parametric methods should be used for small sample sizes. Where little statistical analysis has been undertaken but where there is no evidence of bias, the question should be answered yes. If the distribution of the data (normal or not) is not described it must be assumed that the estimates used were appropriate and the question should be answered yes.

| yes | 1 |
| --- | --- |
| no | 0 |
| unable to determine | 0 |

19. Was compliance with the intervention/s reliable? Where there was non compliance with the allocated treatment or where there was contamination of one group, the question should be answered no. For studies where the effect of any misclassification was likely to bias any association to the null, the question should be answered yes.

| yes | 1 |
| --- | --- |
| no | 0 |
| unable to determine | 0 |

20. Were the main outcome measures used accurate (valid and reliable)?

For studies where the outcome measures are clearly described, the question should be answered yes. For studies which refer to other work or that demonstrates the outcome measures are accurate, the ques- tion should be answered as yes.

| yes | 1 |
| --- | --- |
| no | 0 |
| unable to determine | 0 |

Internal validity - confounding (selection bias)

21. Were the patients in different intervention groups (trials and cohort studies) or were the cases and controls (case-control studies) recruited from the same population?

For example, patients for all comparison groups should be selected from the same hospital. The question should be answered unable to determine for cohort and case- control studies where there is no information concerning the source of patients included in the study.

| yes | 1 |
| --- | --- |
| no | 0 |
| unable to determine | 0 |

22. Were study subjects in different intervention groups (trials and cohort studies) or were the cases and controls (case-control studies) recruited over the same period of time?

For a study which does not specify the time period over which patients were recruited, the question should be answered as unable to determine.

| yes | 1 |
| --- | --- |
| no | 0 |
| unable to determine | 0 |

23. Were study subjects randomised to intervention groups? Studies which state that subjects were randomised should be answered yes except where method of randomisation would not ensure random allocation. For example alternate allocation would score no because it is predictable.

| yes | 1 |
| --- | --- |
| no | 0 |
| unable to determine | 0 |

24. Was the randomised intervention assignment concealed from both patients and health care staff until recruitment was complete and irrevocable?

| yes | 1 |
| --- | --- |
| no | 0 |
| unable to determine | 0 |

25. Was there adequate adjustment for confounding in the analyses from which the main findings were drawn?

This question should be answered no for trials if: the main conclusions of the study were based on analyses of treatment rather than intention to treat; the distribution of known confounders in the different treatment groups was not described; or the distribution of known confounders differed between the treatment groups but was not taken into account in the analyses. In non-randomized studies if the effect of the main confounders was not investigated or con- founding was demonstrated but no adjustment was made in the final analyses the question should be answered as no.

| yes | 1 |
| --- | --- |
| no | 0 |
| unable to determine | 0 |

26. Were losses of patients to follow-up taken into account?

If the numbers of patients lost to follow-up are not reported, the question should be answered as unable to determine. If the proportion lost to follow-up was too small to affect the main findings, the question should be answered yes.

| yes | 1 |
| --- | --- |
| no | 0 |
| unable to determine | 0 |

Power

27. Did the study perform calculations to determine sufficient power to detect a clinically important difference?

Sample sizes have been calculated to detect a difference of x% and y%.

| Yes | 1 |
| --- | --- |
| No | 0 |

**Total score: _____**

**Supplementary Appendix 1.** Search terms and strategies

| **Vaccines** | **COVID 19** | **Efficacy** | **Immunocompromised patients** |
| --- | --- | --- | --- |
| Medical subject headings (PubMed)  Covid-19 Vaccines  RNA, Messenger  Vaccines  Vaccination  Immunization | Medical subject headings (PubMed)  COVID 19  SARS CoV 2  Coronavirus infections | Medical subject headings (PubMed)  vaccine efficacy  treatment outcome  Antibodies, Neutralizing | Medical subject headings (PubMed)  Immunocompromised host  Organ transplantation  Hematopoietic stem cell transplantation  HIV  Acquired Immunodeficiency Syndrome  Thalassemia  Neoplasms  antineoplastic combined chemotherapy protocols chemotherapy, adjuvant  adrenal cortex hormones glucocorticoids hydroxycorticosteroids  17 ketosteroids immunologic factors immunosuppressive agents interferons  mast cell stabilizers agglutinins  tumor necrosis factor inhibitors infliximab  adalimumab  certolizumab pegol etanercept  janus kinase inhibitors immune checkpoint inhibitors abatacept  rituximab |
| Free text words/phrases  vaccine  Vaccinations  pfizer  bnt162b2  moderna immunizations variolation variolations  immunologic stimulation immunostimulation  mrna 1273  chadox1 s  azd 1222  astrazeneca oxford  janssen  johnson johnson  ad26covs1  jnj 78436735  gamaleya  sputnik v  sinovac  corona vac  novavax  COVID-19 vaccine  heterologous | Free text words/phrases  covid  corona virus  coronavirus  2019 ncov | Free text words/phrases  effectiveness  efficacy  effective | Free text words/phrases  17 ketosteroid (s)  abatacept  ABT 494  Acquired immune deficiency syndrome (s)  Acquired immuno deficiency syndrome[Mesh]  Acquired Immuno deficiency syndromes  Acquired Immunodeficiency Syndrome (s)  Actemra  adalimumab (s)  Adalimumab adbm  Adalimumab atto  Adjuvant chemotherap (y, ies)[Mesh]  adrenal cortex hormone (5)  agglutinin (s)  AIDS  Amjevita  Anticancer Drug Combination  Anticancer Drug Combinations  Antilymphocyte Serum  antineoplastic combined chemotherapy protocol (s)[Mesh]  Antineoplastic Combined Chemotherapy Regimens  Antineoplastic Drug Combination  Antineoplastic Drug Combinations  ASP015K  atlizumad  Azaserine  Azathioprine (s)  Baricitinib  Basiliximab  Belatacept  beta Type Microcytemia  beta Type Microcytemias  Biologic agents)  BMS188667  BMS 188667  BMS 224818  BMS224818  BMS188667  BMS224818  BMS 188667  BMS 224818  Busulfan  Cancer(s)  cart cell therap (V. les)  CD-20 inhibitor (s)  0D59 Antigen (S)  CDP 870  CDP870  Certolizumab  certolizumab pegol  Chemotherap (y, ies)  Cimzial  Cladribine  CNTO148  CNTO 148  Coformycin (s)  Combined Antineoplastic Agent  Combined Antineoplastic Agents  Complement C1 Inactivator Proteins  Complement C1 Inhibitor Protein  Complement C3 Nephritic Factor  Complement C3b Inactivator Proteins  Complement CAb-Binding Protein  Complement Factor H  Complement Factor I  Complement Inactivator Proteins  Cooley's Anemia  Corticoid  Corticoids  Corticosteroid  Corticosteroids  CP 690550  CP690550  CTLA 4 Inhibitor  CTLA 4 Inhibitors  CTLA4 lg Immunoconjugate  CTLA4-Fc  CTLA4-Ig  CTLA-4-Ig  Cyclophosphamide (s)  Cyclosporine (s)  Cyclosporin (s)  Cyltezo  Cytarabine  Cytotoxic T Lymphocyte Associated Antigen 4 Immunoglobulin  Cytotoxic T Lymphocyte  Associated Protein 4 Inhibitor  Cytotoxic T Lymphocyte  Associated Protein 4 Inhibitors  DE7 Antibody  Daclizumab  Dimethyl Fumarate  Ellipticine (s)  Enbrel  Erelzi  Erythroblastic Anemia (s)  Etanercept  Etanercept-szzs  Everolimus  Fingolimod Hydrochloride  Fluorouracil (s)  Glatiramer Acetate  Gliotoxin (s)  Glucocorticoid  glucocorticoids  Golimumab  GP2013  Hematopoietic stem cell transplantation (s)[Mesh]  Hemoglobin F disease  Hemoglobin H disease (s)  HIV[Mesh]  Human immunodeficiency virus  Human immunodeficiency viruses  Human T Cell Leukemia Virus Type III  Human T Cell Lymphotropic Virus Type III  Human T Lymphotropic Virus Type III  Human T-Cell Leukemia Virus Type III  Human T-Cell Lymphotropic Virus Type III  Human T-Lymphotropic Virus Type III  Humira hydroxycorticosteroid (s)  IDEC C2B8  IDECC2B8  IDEC C2B8 Antibody  Immune Checkpoint Blockade  Immune Checkpoint Blockers  Immune Checkpoint Inhibition (s)  Immune Checkpoint Inhibitor immune checkpoint inhibitors immunocompromise  Immunocompromised  Immunocompromised host[Mesh]  Immunodeficienc (y, ies)  Immunodeficient immunodepression (s) immunologic factor (s)  Immunomodulating agents)  Immunomodulator(s)  immunosuppressants) immunosuppressed immunosuppression  Immunosuppressive immunosuppressive agents  Inflectra infliximab (s)  interferon (s)  JAK inhibitor(s)  Janus Kinase Inhibitor janus kinase inhibitors  Kevzara  LAV-HTLV-III  LEA29Y  Leflunomide  Leukemia  LY3009104  Lymphadenopathy Associated Virus Lymphadenopathy-Associated Viruses  Lymphoma (s)  Mathera  Malignancies  Malignancy  mast cell stabilizer (s)  Mediterranean Anemia  Mediterranean Anemias  Melphalan  Mercaptopurine (s)  Methotrexate (s)  Monoclonal antibody cA2  Multiple myeloma  Muromonab-CD3  Neoplasm  Neoplasms[Mesh]  Nulojix  Olumiant  Orencia  Organ Grafting(s)  Organ transplantation[Mesh] organ transplantations  PD 1 Inhibitor  PD 1 Inhibitors  PD 1 PD L1 Blockade  PD L1 Inhibitor  PD L1 Inhibitors  Peficitinib  Programmed Cell Death Protein 1 Inhibitor  Programmed Cell Death Protein 1 Inhibitors  Programmed Death Ligand 1 Inhibitor (s)  R-1569  Radiation chimera(s)  Recombinant Human Dimeric TNF Receptor Type Il IgG Fusion Protein  REGN88  REGN-88  Remicade  Renflexis  RHPM-1  RHPM1  Rinvoq  Rituxan  rituximab (s)  RO-4877533  Roactemra  SAR153191  SAR-153191  Sarilumab  Simponi  Sirolimus  Steroid (s)  T Cell stimulation inhibitor (s)  Tacrolimus  Thalassemia[Mesh]  Thalassemias  Thalidomide (s)  Thioinosine  Thiotepa  TNF antagonist (s)  TNF blocker (s)  TNF inhibitor (s)  TNF Receptor Type Il IgG Fusion Protein  TNFR Fc Fusion Protein  TNR 001  TNT Receptor Fusion Protein  Tocilizumab  Tofacitinib  Triamcinolone Acetonide  Tumor necrosis factor blocker (s) tumor necrosis factor inhibitor (s)  Upadacitinib  Wortmannin (s)  Xeljanz |

**Pubmed**

(((((((covid 19[MeSH Terms]) OR (sars cov 2[MeSH Terms])) OR (coronavirus infections[MeSH Terms])) AND (((((covid 19 vaccines[MeSH Terms]) OR (rna, messenger[MeSH Terms])) OR (vaccines[MeSH Terms])) OR (immunization[MeSH Terms])) OR (vaccination[MeSH Terms]))) AND (((treatment outcome[MeSH Terms]) OR (vaccine efficacy[MeSH Terms])) OR (antibodies, neutralizing[MeSH Terms]))) AND (((((((((((((((((((((((((((immunocompromised host[MeSH Terms]) OR (organ transplantation[MeSH Terms])) OR (hematopoietic stem cell transplantation[MeSH Terms])) OR (hiv[MeSH Terms])) OR (acquired immunodeficiency syndrome[MeSH Terms])) OR (thalassemia[MeSH Terms])) OR (neoplasms[MeSH Terms])) OR (antineoplastic combined chemotherapy protocols[MeSH Terms])) OR (chemotherapy, adjuvant[MeSH Terms])) OR (adrenal cortex hormones[MeSH Terms])) OR (glucocorticoids[MeSH Terms])) OR (hydroxycorticosteroids[MeSH Terms])) OR (17 ketosteroids[MeSH Terms])) OR (immunologic factors[MeSH Terms])) OR (immunosuppressive agents[MeSH Terms])) OR (interferons[MeSH Terms])) OR (mast cell stabilizers[MeSH Terms])) OR (agglutinins[MeSH Terms])) OR (tumor necrosis factor inhibitors[MeSH Terms])) OR (infliximab[MeSH Terms])) OR (adalimumab[MeSH Terms])) OR (certolizumab pegol[MeSH Terms])) OR (etanercept[MeSH Terms])) OR (janus kinase inhibitors[MeSH Terms])) OR (immune checkpoint inhibitors[MeSH Terms])) OR (abatacept[MeSH Terms])) OR (rituximab[MeSH Terms]))) OR ((((((((((covid 19[Title/Abstract]) OR (sars cov 2[Title/Abstract])) OR (coronavirus infections[Title/Abstract])) OR (covid[Title/Abstract])) OR (corona virus[Title/Abstract])) OR (coronavirus[Title/Abstract])) OR (2019 ncov[Title/Abstract])) AND ((((((vaccine efficacy[Title/Abstract]) OR (effectiveness[Title/Abstract])) OR (efficacy[Title/Abstract])) OR (effective[Title/Abstract])) OR (treatment outcome[Title/Abstract])) OR (neutralizing antibodies[Title/Abstract]))) AND ((("covid 19 vaccines"[Title/Abstract] OR "messenger rna" [Title/Abstract] OR "vaccines"[Title/Abstract] OR "vaccination" [Title/Abstract] OR "immunization"[Title/Abstract] OR "vaccine" [Title/Abstract] OR "vaccinations"[Title/Abstract] OR "pfizer"[Title/Abstract] OR "bnt162b2"[Title/Abstract] OR "moderna" [Title/Abstract] OR "immunizations"[Title/Abstract] OR "variolation" [Title/Abstract] OR "variolations"[Title/Abstract] OR "immunologic stimulation"[Title/Abstract] OR "immunostimulation"[Title/Abstract] OR "mrna 1273"[Title/Abstract] OR "chadox1 s"[Title/Abstract] OR "azd1222" [Title/Abstract] OR "astrazeneca oxford"[Title/Abstract] OR "janssen" [Title/Abstract] OR "johnson johnson"[Title/Abstract] OR "ad26covs1" [Title/Abstract] OR "jnj 78436735"[Title/Abstract] OR "gamaleya" [Title/Abstract] OR "sputnik v"[Title/Abstract] OR "sinovac"[Title/Abstract] OR "corona vac"[Title/Abstract] OR "novavax"[Title/Abstract] OR "covaxin" [Title/Abstract] OR "sinopharm"[Title/Abstract] OR "vaccinated" [Title/Abstract]) OR (Vaccine*[Title/Abstract])) OR (Vaccination* [Title/Abstract]))) AND (((((((((((((((((((((((((((((((((((((((((((((((((((((((((((((((((((((((((((((((((((((((((((((((( (((((((((((((((((((((((((((((((((((((((((((((((((((((((((((((((((((((((((((((((((((((((((((((((( (((((((((((((((((((((((((((((((((((((((((((((((((((((((((((((((((((((((((((((((17 ketosteroids[Title/Abstract]) OR (17 ketosteroid[Title/Abstract])) OR (abatacept[Title/Abstract])) OR (abt 494[Title/Abstract])) OR (acquired immune deficiency syndrome[Title/Abstract])) OR (acquired immune deficiency syndromes[Title/Abstract])) OR (acquired immuno deficiency syndrome[Title/Abstract])) OR (acquired immuno deficiency syndromes[Title/Abstract])) OR (acquired immunodeficiency syndrome[Title/Abstract])) OR (acquired immunodeficiency syndromes[Title/Abstract])) OR (actemra[Title/Abstract])) OR (adalimumab[Title/Abstract])) OR (adalimumabs[Title/Abstract])) OR (adalimumab adbm[Title/Abstract])) OR (adalimumab atto[Title/Abstract])) OR (adjuvant chemotherapy[Title/Abstract])) OR (adjuvant chemotherapies[Title/Abstract])) OR (adrenal cortex hormones[Title/Abstract])) OR (adrenal cortex hormone[Title/Abstract])) OR (agglutinins[Title/Abstract])) OR (agglutinin[Title/Abstract])) OR (aids[Title/Abstract])) OR (Amjevita[Title/Abstract])) OR (anticancer drug combination[Title/Abstract])) OR (anticancer drug combinations[Title/Abstract])) OR (antilymphocyte serum[Title/Abstract])) OR (antineoplastic combined chemotherapy protocols[Title/Abstract])) OR (antineoplastic combined chemotherapy protocol[Title/Abstract])) OR (antineoplastic drug combinations[Title/Abstract])) OR (asp015k[Title/Abstract])) OR (atlizumab[Title/Abstract])) OR (azaserine[Title/Abstract])) OR (azathioprine[Title/Abstract])) OR (azathioprines[Title/Abstract])) OR (baricitinib[Title/Abstract])) OR (basiliximab[Title/Abstract])) OR (belatacept[Title/Abstract])) OR (biologic agent[Title/Abstract])) OR (biologic agents[Title/Abstract])) OR (bms 188667[Title/Abstract])) OR (bms188667[Title/Abstract])) OR (bms 224818[Title/Abstract])) OR (bms224818[Title/Abstract])) OR (bms188667[Title/Abstract])) OR (bms 188667[Title/Abstract])) OR (bms224818[Title/Abstract])) OR (bms 224818[Title/Abstract])) OR (busulfan[Title/Abstract])) OR (cancer[Title/Abstract])) OR (cancers[Title/Abstract])) OR (car t cell therapy[Title/Abstract])) OR (car t cell therapies[Title/Abstract])) OR (cd59 antigens[Title/Abstract])) OR (cd59 antigen[Title/Abstract])) OR (cdp 870[Title/Abstract])) OR (cdp870[Title/Abstract])) OR (certolizumab[Title/Abstract])) OR (certolizumab pegol[Title/Abstract])) OR (chemotherapy[Title/Abstract])) OR (chemotherapies[Title/Abstract])) OR (cimzia[Title/Abstract])) OR (cladribine[Title/Abstract])) OR (cnto 148[Title/Abstract])) OR (cnto148[Title/Abstract])) OR (coformycin[Title/Abstract])) OR (coformycins[Title/Abstract])) OR (combined antineoplastic agents[Title/Abstract])) OR (complement c1 inactivator proteins[Title/Abstract])) OR (complement c1 inhibitor protein[Title/Abstract])) OR (complement c3 nephritic factor[Title/Abstract])) OR (complement c4b binding protein[Title/Abstract])) OR (complement factor h[Title/Abstract])) OR (complement factor i[Title/Abstract])) OR (cooley s anemia[Title/Abstract])) OR (corticoid[Title/Abstract])) OR (corticoids[Title/Abstract])) OR (corticosteroid[Title/Abstract])) OR (corticosteroids[Title/Abstract])) OR (cp 690550[Title/Abstract])) OR (cp690550[Title/Abstract])) OR (ctla 4 inhibitor[Title/Abstract])) OR (ctla 4 inhibitors[Title/Abstract])) OR (ctla4 ig immunoconjugate[Title/Abstract])) OR (ctla4 fc[Title/Abstract])) OR (ctla4 ig[Title/Abstract])) OR (ctla 4 ig[Title/Abstract])) OR (cyclophosphamide[Title/Abstract])) OR (cyclophosphamides[Title/Abstract])) OR (cyclosporine[Title/Abstract])) OR (cyclosporines[Title/Abstract])) OR (cyclosporins[Title/Abstract])) OR (cyclosporin[Title/Abstract])) OR (cyltezo[Title/Abstract])) OR (cytarabine[Title/Abstract])) OR (cytotoxic t lymphocyte associated antigen 4 immunoglobulin[Title/Abstract])) OR (cytotoxic t lymphocyte associated protein 4 inhibitor[Title/Abstract])) OR (cytotoxic t lymphocyte associated protein 4 inhibitors[Title/Abstract])) OR (daclizumab[Title/Abstract])) OR (dimethyl fumarate[Title/Abstract])) OR (ellipticines[Title/Abstract])) OR (ellipticine[Title/Abstract])) OR (enbrel[Title/Abstract])) OR (erelzi[Title/Abstract])) OR (erythroblastic anemia[Title/Abstract])) OR (erythroblastic anemias[Title/Abstract])) OR (etanercept[Title/Abstract])) OR (etanercepts[Title/Abstract])) OR (etanercept szzs[Title/Abstract])) OR (everolimus[Title/Abstract])) OR (fingolimod hydrochloride[Title/Abstract])) OR (fluorouracil[Title/Abstract])) OR (fluorouracils[Title/Abstract])) OR (glatiramer acetate[Title/Abstract])) OR (gliotoxin[Title/Abstract])) OR (gliotoxins[Title/Abstract])) OR (glucocorticoid[Title/Abstract])) OR (glucocorticoids[Title/Abstract])) OR (golimumab[Title/Abstract])) OR (gp2013[Title/Abstract])) OR (gp 2013[Title/Abstract])) OR (hematopoietic stem cell transplantation[Title/Abstract])) OR (hematopoietic stem cell transplantations[Title/Abstract])) OR (hemoglobin h disease[Title/Abstract])) OR (hemoglobin h diseases[Title/Abstract])) OR (hiv[Title/Abstract])) OR (human immunodeficiency virus[Title/Abstract])) OR (human immunodeficiency viruses[Title/Abstract])) OR (human t cell leukemia virus type iii[Title/Abstract])) OR (human t cell lymphotropic virus type iii[Title/Abstract])) OR (human t lymphotropic virus type iii[Title/Abstract])) OR (human t cell leukemia virus type iii[Title/Abstract])) OR (human t cell lymphotropic virus type iii[Title/Abstract])) OR (human t lymphotropic virus type iii[Title/Abstract])) OR (humira[Title/Abstract])) OR (hydroxycorticosteroids[Title/Abstract])) OR (hydroxycorticosteroid[Title/Abstract])) OR (idec c2b8[Title/Abstract])) OR (idecc2b8[Title/Abstract])) OR (idec c2b8 antibody[Title/Abstract])) OR (immune checkpoint blockade[Title/Abstract])) OR (immune checkpoint blockers[Title/Abstract])) OR (immune checkpoint inhibition[Title/Abstract])) OR (immune checkpoint inhibitions[Title/Abstract])) OR (immune checkpoint inhibitor[Title/Abstract])) OR (immune checkpoint inhibitors[Title/Abstract])) OR (immunocompromise[Title/Abstract])) OR (immunocompromised[Title/Abstract])) OR (immunocompromised host[Title/Abstract])) OR (immunodeficiency[Title/Abstract])) OR (immunodeficiencies[Title/Abstract])) OR (immunodeficient[Title/Abstract])) OR (immunodepression[Title/Abstract])) OR (immunodepressions[Title/Abstract])) OR (immunologic factors[Title/Abstract])) OR (immunologic factor[Title/Abstract])) OR (immunomodulating agent[Title/Abstract])) OR (immunomodulating agents[Title/Abstract])) OR (immunomodulator[Title/Abstract])) OR (immunomodulators[Title/Abstract])) OR (immunosuppressant[Title/Abstract])) OR (immunosuppressants[Title/Abstract])) OR (immunosuppressed[Title/Abstract])) OR (immunosuppression[Title/Abstract])) OR (immunosuppressive[Title/Abstract])) OR (immunosuppressive agents[Title/Abstract])) OR (inflectra[Title/Abstract])) OR (infliximab[Title/Abstract])) OR (infliximabs[Title/Abstract])) OR (interferons[Title/Abstract])) OR (interferon[Title/Abstract])) OR (jak inhibitor[Title/Abstract])) OR (jak inhibitors[Title/Abstract])) OR (janus kinase inhibitor[Title/Abstract])) OR (janus kinase inhibitors[Title/Abstract])) OR (kevzara[Title/Abstract])) OR (lav htlv iii[Title/Abstract])) OR (lavhtlviii[Title/Abstract])) OR (lea29y[Title/Abstract])) OR (leflunomide[Title/Abstract])) OR (leukemia[Title/Abstract])) OR (leukemias[Title/Abstract])) OR (ly3009104[Title/Abstract])) OR (ly 3009104[Title/Abstract])) OR (lymphadenopathy associated virus[Title/Abstract])) OR (lymphoma[Title/Abstract])) OR (lymphomas[Title/Abstract])) OR (mabthera[Title/Abstract])) OR (malignancies[Title/Abstract])) OR (malignancy[Title/Abstract])) OR (mast cell stabilizers[Title/Abstract])) OR (mast cell stabilizer[Title/Abstract])) OR (mediterranean anemia[Title/Abstract])) OR (mediterranean anemias[Title/Abstract])) OR (melphalan[Title/Abstract])) OR (mercaptopurine[Title/Abstract])) OR (mercaptopurines[Title/Abstract])) OR (methotrexate[Title/Abstract])) OR (methotrexates[Title/Abstract])) OR (monoclonal antibody ca2[Title/Abstract])) OR (multiple myeloma[Title/Abstract])) OR (muromonab cd3[Title/Abstract])) OR (muromonabcd3[Title/Abstract])) OR (neoplasm[Title/Abstract])) OR (neoplasms[Title/Abstract])) OR (nulojix[Title/Abstract])) OR (olumiant[Title/Abstract])) OR (orencia[Title/Abstract])) OR (organ grafting[Title/Abstract])) OR (organ graftings[Title/Abstract])) OR (organ transplantation[Title/Abstract])) OR (organ transplantations[Title/Abstract])) OR (pd 1 inhibitor[Title/Abstract])) OR (pd 1 inhibitors[Title/Abstract])) OR (pd 1 pd l1 blockade[Title/Abstract])) OR (pd l1 inhibitor[Title/Abstract])) OR (pd l1 inhibitors[Title/Abstract])) OR (peficitinib[Title/Abstract])) OR (programmed cell death protein 1 inhibitor[Title/Abstract])) OR (programmed cell death protein 1 inhibitors[Title/Abstract])) OR (programmed death ligand 1 inhibitors[Title/Abstract])) OR (programmed death ligand 1 inhibitor[Title/Abstract])) OR (r 1569[Title/Abstract])) OR (radiation chimera[Title/Abstract])) OR (radiation chimeras[Title/Abstract])) OR (recombinant human dimeric tnf receptor type ii igg fusion protein[Title/Abstract])) OR (regn88[Title/Abstract])) OR (regn 88[Title/Abstract])) OR (remicade[Title/Abstract])) OR (renflexis[Title/Abstract])) OR (rhpm 1[Title/Abstract])) OR (rhpm1[Title/Abstract])) OR (rinvoq[Title/Abstract])) OR (rituxan[Title/Abstract])) OR (rituximab[Title/Abstract])) OR (rituximabs[Title/Abstract])) OR (ro 4917838[Title/Abstract])) OR (roactemra[Title/Abstract])) OR (sar153191[Title/Abstract])) OR (sar 153191[Title/Abstract])) OR (sarilumab[Title/Abstract])) OR (simponi[Title/Abstract])) OR (sirolimus[Title/Abstract])) OR (steroid[Title/Abstract])) OR (steroids[Title/Abstract])) OR (tacrolimus[Title/Abstract])) OR (thalassemia[Title/Abstract])) OR (thalassemias[Title/Abstract])) OR (thalidomide[Title/Abstract])) OR (thalidomides[Title/Abstract])) OR (thioinosine[Title/Abstract])) OR (thiotepa[Title/Abstract])) OR (tnf antagonists[Title/Abstract])) OR (tnf antagonist[Title/Abstract])) OR (tnf blocker[Title/Abstract])) OR (tnf blockers[Title/Abstract])) OR (tnf inhibitor[Title/Abstract])) OR (tnf inhibitors[Title/Abstract])) OR (tnf receptor type ii igg fusion protein[Title/Abstract])) OR (tnfr fc fusion protein[Title/Abstract])) OR (tnr 001[Title/Abstract])) OR (tocilizumab[Title/Abstract])) OR (tofacitinib[Title/Abstract])) OR (triamcinolone acetonide[Title/Abstract])) OR (tumor necrosis factor blocker[Title/Abstract])) OR (tumor necrosis factor blockers[Title/Abstract])) OR (tumor necrosis factor inhibitors[Title/Abstract])) OR (tumor necrosis factor inhibitor[Title/Abstract])) OR (upadacitinib[Title/Abstract])) OR (wortmannin[Title/Abstract])) OR (wortmannins[Title/Abstract])) OR (xeljanz[Title/Abstract])))) OR (("covid 19"[Text Word] OR "sars cov 2"[Text Word] OR "coronavirus infections"[Text Word] OR "covid"[Text Word] OR "corona virus"[Text Word] OR "coronavirus"[Text Word] OR "2019 ncov" [Text Word]) AND ("vaccine efficacy"[Text Word] OR "effectiveness"[Text Word] OR "efficacy"[Text Word] OR "effective"[Text Word] OR "treatment outcome"[Text Word] OR "neutralizing antibodies"[Text Word]) AND ("covid 19 vaccines"[Text Word] OR "messenger rna"[Text Word] OR "vaccines"[Text Word] OR "vaccination"[Text Word] OR "immunization" [Text Word] OR "vaccine"[Text Word] OR "vaccinations"[Text Word] OR "pfizer"[Text Word] OR "bnt162b2"[Text Word] OR "moderna"[Text Word] OR "immunizations"[Text Word] OR "variolation"[Text Word] OR "variolations"[Text Word] OR "immunologic stimulation"[Text Word] OR "immunostimulation"[Text Word] OR "mrna 1273"[Text Word] OR "chadox1 s"[Text Word] OR "azd1222"[Text Word] OR "astrazeneca oxford" [Text Word] OR "janssen"[Text Word] OR "johnson johnson"[Text Word] OR "ad26covs1"[Text Word] OR "jnj 78436735"[Text Word] OR "gamaleya" [Text Word] OR "sputnik v"[Text Word] OR "sinovac"[Text Word] OR "corona vac"[Text Word] OR "novavax"[Text Word] OR "covaxin"[Text Word] OR "sinopharm"[Text Word] OR "vaccinated"[Text Word] OR "vaccine*"[Text Word] OR "vaccination*"[Text Word]) AND ("17 ketosteroids"[Text Word] OR "17 ketosteroid"[Text Word] OR "abatacept" [Text Word] OR "abt 494"[Text Word] OR "acquired immune deficiency syndrome"[Text Word] OR "acquired immune deficiency syndromes"[Text Word] OR "acquired immuno deficiency syndrome"[Text Word] OR "acquired immuno deficiency syndromes"[Text Word] OR "acquired immunodeficiency syndrome"[Text Word] OR "acquired immunodeficiency syndromes"[Text Word] OR "actemra"[Text Word] OR "adalimumab"[Text Word] OR "adalimumabs"[Text Word] OR "adalimumab adbm"[Text Word] OR "adalimumab atto"[Text Word] OR "adjuvant chemotherapy"[Text Word] OR "adjuvant chemotherapies"[Text Word] OR "adrenal cortex hormones"[Text Word] OR "adrenal cortex hormone"[Text Word] OR "agglutinins"[Text Word] OR "agglutinin"[Text Word] OR "aids"[Text Word] OR "Amjevita"[Text Word] OR "anticancer drug combination"[Text Word] OR "anticancer drug combinations"[Text Word] OR "antilymphocyte serum"[Text Word] OR "antineoplastic combined chemotherapy protocols" [Text Word] OR "antineoplastic combined chemotherapy protocol"[Text Word] OR "antineoplastic drug combinations"[Text Word] OR "asp015k" [Text Word] OR "atlizumab"[Text Word] OR "azaserine"[Text Word] OR "azathioprine"[Text Word] OR "azathioprines"[Text Word] OR "baricitinib" [Text Word] OR "basiliximab"[Text Word] OR "belatacept"[Text Word] OR "biologic agent"[Text Word] OR "biologic agents"[Text Word] OR "bms 188667"[Text Word] OR "bms188667"[Text Word] OR "bms 224818"[Text Word] OR "bms224818"[Text Word] OR "bms188667"[Text Word] OR "bms 188667"[Text Word] OR "bms224818"[Text Word] OR "bms 224818"[Text Word] OR "busulfan"[Text Word] OR "cancer"[Text Word] OR "cancers" [Text Word] OR "car t cell therapy"[Text Word] OR "car t cell therapies" [Text Word] OR "cd59 antigens"[Text Word] OR "cd59 antigen"[Text Word] OR "cdp 870"[Text Word] OR "cdp870"[Text Word] OR "certolizumab"[Text Word] OR "certolizumab pegol"[Text Word] OR "chemotherapy"[Text Word] OR "chemotherapies"[Text Word] OR "cimzia"[Text Word] OR "cladribine"[Text Word] OR "cnto 148"[Text Word] OR "cnto148"[Text Word] OR "coformycin"[Text Word] OR "coformycins"[Text Word] OR "combined antineoplastic agents"[Text Word] OR "complement c1 inactivator proteins"[Text Word] OR "complement c1 inhibitor protein" [Text Word] OR "complement c3 nephritic factor"[Text Word] OR "complement c4b binding protein"[Text Word] OR "complement factor h" [Text Word] OR "complement factor i"[Text Word] OR "cooley s anemia" [Text Word] OR "corticoid"[Text Word] OR "corticoids"[Text Word] OR "corticosteroid"[Text Word] OR "corticosteroids"[Text Word] OR "cp 690550"[Text Word] OR "cp690550"[Text Word] OR "ctla 4 inhibitor"[Text Word] OR "ctla 4 inhibitors"[Text Word] OR "ctla4 ig immunoconjugate" [Text Word] OR "ctla4 fc"[Text Word] OR "ctla4 ig"[Text Word] OR "ctla 4 ig"[Text Word] OR "cyclophosphamide"[Text Word] OR "cyclophosphamides"[Text Word] OR "cyclosporine"[Text Word] OR "cyclosporines"[Text Word] OR "cyclosporins"[Text Word] OR "cyclosporin" [Text Word] OR "cyltezo"[Text Word] OR "cytarabine"[Text Word] OR "cytotoxic t lymphocyte associated antigen 4 immunoglobulin"[Text Word] OR "cytotoxic t lymphocyte associated protein 4 inhibitor"[Text Word] OR "cytotoxic t lymphocyte associated protein 4 inhibitors"[Text Word] OR "daclizumab"[Text Word] OR "dimethyl fumarate"[Text Word] OR "ellipticines"[Text Word] OR "ellipticine"[Text Word] OR "enbrel"[Text Word] OR "erelzi"[Text Word] OR "erythroblastic anemia"[Text Word] OR "erythroblastic anemias"[Text Word] OR "etanercept"[Text Word] OR "etanercepts"[Text Word] OR "etanercept szzs"[Text Word] OR "everolimus"[Text Word] OR "fingolimod hydrochloride"[Text Word] OR "fluorouracil"[Text Word] OR "fluorouracils"[Text Word] OR "glatiramer acetate"[Text Word] OR "gliotoxin"[Text Word] OR "gliotoxins"[Text Word] OR "glucocorticoid"[Text Word] OR "glucocorticoids"[Text Word] OR "golimumab"[Text Word] OR "gp2013"[Text Word] OR "gp 2013"[Text Word] OR "hematopoietic stem cell transplantation"[Text Word] OR "hematopoietic stem cell transplantations"[Text Word] OR "hemoglobin h disease"[Text Word] OR "hemoglobin h diseases"[Text Word] OR "hiv"[Text Word] OR "human immunodeficiency virus"[Text Word] OR "human immunodeficiency viruses"[Text Word] OR "human t cell leukemia virus type iii"[Text Word] OR "human t cell lymphotropic virus type iii"[Text Word] OR "human t lymphotropic virus type iii"[Text Word] OR "human t cell leukemia virus type iii"[Text Word] OR "human t cell lymphotropic virus type iii"[Text Word] OR "human t lymphotropic virus type iii"[Text Word] OR "humira"[Text Word] OR "hydroxycorticosteroids"[Text Word] OR "hydroxycorticosteroid"[Text Word] OR "idec c2b8"[Text Word] OR "idecc2b8"[Text Word] OR "idec c2b8 antibody"[Text Word] OR "immune checkpoint blockade"[Text Word] OR "immune checkpoint blockers"[Text Word] OR "immune checkpoint inhibition"[Text Word] OR "immune checkpoint inhibitions"[Text Word] OR "immune checkpoint inhibitor"[Text Word] OR "immune checkpoint inhibitors"[Text Word] OR "immunocompromise"[Text Word] OR "immunocompromised"[Text Word] OR "immunocompromised host"[Text Word] OR "immunodeficiency"[Text Word] OR "immunodeficiencies"[Text Word] OR "immunodeficient"[Text Word] OR "immunodepression"[Text Word] OR "immunodepressions"[Text Word] OR "immunologic factors"[Text Word] OR "immunologic factor" [Text Word] OR "immunomodulating agent"[Text Word] OR "immunomodulating agents"[Text Word] OR "immunomodulator"[Text Word] OR "immunomodulators"[Text Word] OR "immunosuppressant" [Text Word] OR "immunosuppressants"[Text Word] OR "immunosuppressed"[Text Word] OR "immunosuppression"[Text Word] OR "immunosuppressive"[Text Word] OR "immunosuppressive agents"[Text Word] OR "inflectra"[Text Word] OR "infliximab"[Text Word] OR "infliximabs"[Text Word] OR "interferons"[Text Word] OR "interferon"[Text Word] OR "jak inhibitor"[Text Word] OR "jak inhibitors"[Text Word] OR "janus kinase inhibitor"[Text Word] OR "janus kinase inhibitors"[Text Word] OR "kevzara"[Text Word] OR "lav htlv iii"[Text Word] OR "lavhtlviii" [Text Word] OR "lea29y"[Text Word] OR "leflunomide"[Text Word] OR "leukemia"[Text Word] OR "leukemias"[Text Word] OR "ly3009104"[Text Word] OR "ly 3009104"[Text Word] OR "lymphadenopathy associated virus"[Text Word] OR "lymphoma"[Text Word] OR "lymphomas"[Text Word] OR "mabthera"[Text Word] OR "malignancies"[Text Word] OR "malignancy"[Text Word] OR "mast cell stabilizers"[Text Word] OR "mast cell stabilizer"[Text Word] OR "mediterranean anemia"[Text Word] OR "mediterranean anemias"[Text Word] OR "melphalan"[Text Word] OR "mercaptopurine"[Text Word] OR "mercaptopurines"[Text Word] OR "methotrexate"[Text Word] OR "methotrexates"[Text Word] OR "monoclonal antibody ca2"[Text Word] OR "multiple myeloma"[Text Word] OR "muromonab cd3"[Text Word] OR "muromonabcd3"[Text Word] OR "neoplasm"[Text Word] OR "neoplasms"[Text Word] OR "nulojix"[Text Word] OR "olumiant"[Text Word] OR "orencia"[Text Word] OR "organ grafting"[Text Word] OR "organ graftings"[Text Word] OR "organ transplantation"[Text Word] OR "organ transplantations"[Text Word] OR "pd 1 inhibitor"[Text Word] OR "pd 1 inhibitors"[Text Word] OR "pd 1 pd l1 blockade"[Text Word] OR "pd l1 inhibitor"[Text Word] OR "pd l1 inhibitors"[Text Word] OR "peficitinib"[Text Word] OR "programmed cell death protein 1 inhibitor"[Text Word] OR "programmed cell death protein 1 inhibitors"[Text Word] OR "programmed death ligand 1 inhibitors"[Text Word] OR "programmed death ligand 1 inhibitor"[Text Word] OR "r 1569" [Text Word] OR "radiation chimera"[Text Word] OR "radiation chimeras" [Text Word] OR "recombinant human dimeric tnf receptor type ii igg fusion protein"[Text Word] OR "regn88"[Text Word] OR "regn 88"[Text Word] OR "remicade"[Text Word] OR "renflexis"[Text Word] OR "rhpm 1"[Text Word] OR "rhpm1"[Text Word] OR "rinvoq"[Text Word] OR "rituxan"[Text Word] OR "rituximab"[Text Word] OR "rituximabs"[Text Word] OR "ro 4917838" [Text Word] OR "roactemra"[Text Word] OR "sar153191"[Text Word] OR "sar 153191"[Text Word] OR "sarilumab"[Text Word] OR "simponi"[Text Word] OR "sirolimus"[Text Word] OR "steroid"[Text Word] OR "steroids" [Text Word] OR "tacrolimus"[Text Word] OR "thalassemia"[Text Word] OR "thalassemias"[Text Word] OR "thalidomide"[Text Word] OR "thalidomides"[Text Word] OR "thioinosine"[Text Word] OR "thiotepa" [Text Word] OR "tnf antagonists"[Text Word] OR "tnf antagonist"[Text Word] OR "tnf blocker"[Text Word] OR "tnf blockers"[Text Word] OR "tnf inhibitor"[Text Word] OR "tnf inhibitors"[Text Word] OR "tnf receptor type ii igg fusion protein"[Text Word] OR "tnfr fc fusion protein"[Text Word] OR "tnr 001"[Text Word] OR "tocilizumab"[Text Word] OR "tofacitinib"[Text Word] OR "triamcinolone acetonide"[Text Word] OR "tumor necrosis factor blocker"[Text Word] OR "tumor necrosis factor blockers"[Text Word] OR "tumor necrosis factor inhibitors"[Text Word] OR "tumor necrosis factor inhibitor"[Text Word] OR "upadacitinib"[Text Word] OR "wortmannin"[Text Word] OR "wortmannins"[Text Word] OR "xeljanz"[Text Word])) Filters: from 2019 - 2023 Sort by: Publication Date – 3.454

**Web of Science**

**A** TS=("17 ketosteroid" or "17 ketosteroids" or abatacept or "ABT 494" or ab2494 or "Acquired immune deficiency syndrome" or "Acquired immune deficiency syndromes" or "Acquired immuno deficiency syndrome" or "Acquired immuno deficiency syndromes" or "Acquired Immunodeficiency Syndrome" or "Acquired Immunodeficiency Syndromes" or artemia or adalimumab or "Adalimumab adbm" or "Adalimumab atto" or "Adjuvant chemotherapy" or "Adjuvant chemotherapies" or "adrenal cortex hormone" or "adrenal cortex hormones" or agglutinin or agglutinins or AIDS or amgevita or "Anticancer Drug Combination" or "Anticancer Drug Combinations" or "Antilymphocyte Serum" or "antineoplastic combined chemotherapy protocol" or "antineoplastic combined chemotherapy protocols" or "Antineoplastic Combined Chemotherapy Regimens" or "Antineoplastic Drug Combination" or "Antineoplastic Drug Combinations" or asp2151 or aclizumab or Azaserine or Azathioprine or azathioprine or Baricitinib or Basiliximab or Belatacept or "beta Type Microcytemia" or "beta Type Microcytemias" or "Biologic agent" or "Biologic agents" or "BMS 188667" or bms180661 or "BMS 224818" or BMS224818 or bms180661 or "BMS 188667" or BMS224818 or "BMS 224818" or Busulfan or Cancer or Cancers or "car t cell therapy" or "car t cell therapies" or "CD 20 inhibitor" or "CD 20 inhibitors" or "CD59 Antigen" or "CD59 Antigens" or "CDP 870" or CDP870 or Certolizumab or "certolizumab pegol" or Chemotherapy or Chemotherapies or cinzia or Cladribine or "CNTO 148" or cnto328 or Coformycin or coformycin or "Combined Antineoplastic Agent" or "Combined Antineoplastic Agents" or "Complement C1 Inactivator Proteins" or "Complement C1 Inactivator Protein" or "Complement C3 Nephritic Factor" or "Complement C3b Inactivator Proteins" or "Complement C4b-Binding Protein" or "Complement Factor H" or "Complement Factor I" or "Complement Inactivator Proteins" or "Cooley's Anemia" or Corticoid or Corticoids or Corticosteroid or Corticosteroids or "CP 690550" or CP690550 or "CTLA 4 Inhibitor" or "CTLA 4 Inhibitors" or "CTLA4 Ig Immunoconjugate" or "CTLA4 Fc" or "CTLA4 Ig" or "CTLA 4 Ig" or Cyclophosphamide or cyclophosphamide or Cyclosporine or cyclosporine or Cyclosporin or Cyclosporins or cortezo or Cytarabine or "Cytotoxic T Lymphocyte Associated Antigen 4 Immunoglobulin" or "Cytotoxic T Lymphocyte Associated Protein 4 Inhibitor" or "Cytotoxic T Lymphocyte Associated Protein 4 Inhibitors" or "D2E7 Antibody" or Daclizumab or "Dimethyl Fumarate" or Ellipticine or Ellipticines or Enbrel or erelli or "Erythroblastic Anemia" or "Erythroblastic Anemias" or etanercept or "Etanercept szzs" or Everolimus or "Fingolimod Hydrochloride" or Fluorouracil or fluorouracile or "Glatiramer Acetate" or Gliotoxin or gliotoxin or Glucocorticoid or Glucocorticoids or Golimumab or gp2017 or "GP 2013" or "Hematopoietic stem cell transplantation" or "Hematopoietic stem cell transplantations" or "Hemoglobin F disease" or "Hemoglobin F diseases" or HIV or "Human immunodeficiency virus" or "Human immunodeficiency viruses" or "Human T Cell Leukemia Virus Type III" or "Human T Cell Lymphotropic Virus Type III" or "Human T Lymphotropic Virus Type III" or "Human T-Cell Leukemia Virus Type III" or "Human T-Cell Lymphotropic Virus Type III" or "Human T-Lymphotropic Virus Type III" or Humira or hydroxycorticosteroid or hydroxycorticosteroids or "IDEC C2B8" or IDECC2B8 or "IDEC C2B8 Antibody" or "Immune Checkpoint Blockade" or "Immune Checkpoint Blockers" or "Immune Checkpoint Inhibition" or "Immune Checkpoint Inhibitions" or "Immune Checkpoint Inhibitor" or "Immune Checkpoint Inhibitors" or immunocompromise or Immunocompromised or "Immunocompromised host" or Immunodeficiency or Immunodeficiencies or Immunodeficient or immunodepression or immunodepression or "immunologic factor" or "immunologic factors" or "Immunomodulating agent" or "Immunomodulating agents" or Immunomodulator or Immunomodulators or immunosuppressant or immunosuppressants or immunosuppressed or immunosuppression or Immunosuppressive or "immunosuppressive agents" or inflected or infliximab or infliximab or interferon or interferons or "JAK inhibitor" or "JAK inhibitors" or "Janus Kinase Inhibitor" or "Janus Kinase Inhibitors" or ketzara or "LAV HTLV III" or LAVHTLVIII or leakey or Leflunomide or Leukemia or ly3009014 or "Lymphadenopathy Associated Virus" or "Lymphadenopathy Associated Viruses" or Lymphoma or Lymphomas or Mabthera or Malignancies or Malignancy or "mast cell stabilizer" or "mast cell stabilizers" or "Mediterranean Anemia" or "Mediterranean Anemias" or Melphalan or Mercaptopurine or mercaptopurine or Methotrexate or methotrexatel or "Monoclonal antibody cA2" or "Multiple myeloma" or "Muromonab CD3" or MuromonabCD3 or Neoplasm or Neoplasms or Nulojix or olumniant or orentia or "Organ Grafting" or "Organ Graftings" or "Organ transplantation" or "Organ transplantations" or "PD 1 Inhibitor" or "PD 1 Inhibitors" or "PD 1 PD L1 Blockade" or "PD L1 Inhibitor" or "PD L1 Inhibitors" or Peficitinib or "Programmed Cell Death Protein 1 Inhibitor" or "Programmed Cell Death Protein 1 Inhibitors" or "Programmed Death Ligand 1 Inhibitor" or "Programmed Death Ligand 1 Inhibitors" or "R 1569" or "Radiation chimera" or "Radiation chimeras" or "Recombinant Human Dimeric TNF Receptor Type II IgG Fusion Protein" or regnum or "REGN 88" or Remicade or reflexis or "RHPM 1" or rhp51 or rinvoqt or Rituxan or rituximab or rituximabe or "RO 4877533" or Roactemra or sar153192 or "SAR 153191" or Sarilumab or simpsoni or Sirolimus or Steroid or Steroids or "T Cell stimulation inhibitor" or "T Cell stimulation inhibitors" or Tacrolimus or Thalassemia or Thalassemias or Thalidomide or thalidomide or Thioinosine or Thiotepa or "TNF antagonist" or "TNF antagonists" or "TNF blocker" or "TNF blockers" or "TNF inhibitor" or "TNF inhibitors" or "TNF Receptor Type II IgG Fusion Protein" or "TNFR Fc Fusion Protein" or "TNR 001" or "TNT Receptor Fusion Protein" or Tocilizumab or Tofacitinib or "Triamcinolone Acetonide" or "Tumor necrosis factor blocker" or "Tumor necrosis factor blockers" or "tumor necrosis factor inhibitor" or "tumor necrosis factor inhibitors" or Upadacitinib or Wortmannin or wortmannin or Xeljanz) | 6,040,762 resultados

**B** TS=("Covid-19 Vaccines" or "Messenger RNA" or Vaccines or Vaccination or Immunization or Vaccine or vaccinations or pfizer or bnt162b2 or moderna or immunizations or variolation or variolations or "immunologic stimulation" or immunostimulation or "mrna 1273" or "chadox1 s" or azd1222 or "astrazeneca oxford" or janssen or "johnson johnson" or ad26covs1 or "jnj 78436735" or gamaleya or "sputnik v" or sinovac or "corona vac" or novavax or covaxin or sinopharm or vaccinated) – 780.818

**C** TS=("COVID 19" or "SARS CoV 2" or "Coronavirus infections" or covid or "corona virus" or coronavirus or "2019 ncov") – 501.703

**D** TS=("vaccine efficacy" or "treatment outcome" or "Neutralizing Antibodies" or effectiveness or efficacy or effective) – 5.482.406

A AND B AND C AND D = 3.730

**SCOPUS**

( ( ( TITLE-ABS-KEY ( "covid 19" )  OR  TITLE-ABS-KEY ( "sars cov 2" )  OR  TITLE-ABS-KEY ( "coronavirus infections" ) OR  TITLE-ABS-KEY ( covid ) OR  TITLE-ABS-KEY ( "corona virus" )  OR  TITLE-ABS-KEY ( coronavirus )  OR  TITLE-ABS-KEY ( "2019 ncov" ) ) )  AND  (( TITLE-ABS-KEY ( "treatment outcome" )  OR  TITLE-ABS-KEY (“neutralizing antibodies”)  OR TITLE-ABS-KEY (“vaccine efficacy")  OR TITLE-ABS-KEY ( effectiveness )  OR  TITLE-ABS-KEY ( efficacy )  OR  TITLE-ABS-KEY ( effective ) ) ) AND  ( ( TITLE-ABS-KEY ( vaccine* )  OR TITLE-ABS-KEY ( " covid 19 vaccines " )  OR  TITLE-ABS-KEY ( "messenger RNA" )  OR  TITLE-ABS-KEY ( vaccines )  OR  TITLE-ABS-KEY ( immunization ) OR TITLE-ABS-KEY ( vaccination* )  OR  TITLE-ABS-KEY ( vaccinated )  OR  TITLE-ABS-KEY ( pfizer )  OR  TITLE-ABS-KEY ( bnt162b2 )  OR  TITLE-ABS-KEY ( moderna )  OR  TITLE-ABS-KEY ( immunization* )  OR  TITLE-ABS-KEY ( variolation* )  OR  TITLE-ABS-KEY ( "immunologic stimulation" )  OR  TITLE-ABS-KEY ( immunostimulation )  OR  TITLE-ABS-KEY ( immunotherap* )  OR  TITLE-ABS-KEY ( "mrna 1273" )  OR  TITLE-ABS-KEY ( "chadox1 s" )  OR  TITLE-ABS-KEY ( azd1222 )  OR  TITLE-ABS-KEY ( "astrazeneca oxford" )  OR  TITLE-ABS-KEY ( janssen )  OR  TITLE-ABS-KEY ( "johnson johnson" )  OR  TITLE-ABS-KEY ( ad26covs1 )  OR  TITLE-ABS-KEY ( "jnj 78436735" )  OR  TITLE-ABS-KEY ( gamaleya )  OR  TITLE-ABS-KEY ( "sputnik v" )  OR  TITLE-ABS-KEY ( sinovac )  OR  TITLE-ABS-KEY ( "corona vac" )  OR  TITLE-ABS-KEY ( novavax )  OR  TITLE-ABS-KEY ( covaxin )  OR  TITLE-ABS-KEY ( sinopharm ) ) )  AND  ( ( TITLE-ABS-KEY ( covid )  OR  TITLE-ABS-KEY ( "covid 19" )  OR  TITLE-ABS-KEY ( "corona virus" )  OR  TITLE-ABS-KEY ( coronavirus )  OR  TITLE-ABS-KEY ( "2019 ncov" )  OR  TITLE-ABS-KEY ( "sars cov 2" )  OR  TITLE-ABS-KEY ( "2019 ncov" ) ) ) ) AND ( ( TITLE-ABS-KEY ( 17 ketosteroid ) OR TITLE-ABS-KEY ( 17 ketosteroids ) OR TITLE-ABS-KEY ( abatacept ) OR TITLE-ABS-KEY ( "ABT 494" ) OR TITLE-ABS-KEY ( abt494 ) OR TITLE-ABS-KEY ( "Acquired immune deficiency syndrome" ) OR TITLE-ABS-KEY ( "Acquired immune deficiency syndromes" ) OR TITLE-ABS-KEY ( "Acquired immuno deficiency syndrome" ) OR TITLE-ABS-KEY ( "Acquired immuno deficiency syndromes" ) OR TITLE-ABS-KEY ( "Acquired Immunodeficiency Syndrome" ) OR TITLE-ABS-KEY ( "Acquired Immunodeficiency Syndromes" ) OR TITLE-ABS-KEY ( actemra ) OR TITLE-ABS-KEY ( adalimumab ) OR TITLE-ABS-KEY ( adalimumabs ) OR TITLE-ABS-KEY ( "Adalimumab adbm" ) OR TITLE-ABS-KEY ( "Adalimumab atto" ) OR TITLE-ABS-KEY ( "Adjuvant chemotherapy" ) OR TITLE-ABS-KEY ( "Adjuvant chemotherapies" ) OR TITLE-ABS-KEY ( "adrenal cortex hormone" ) OR TITLE-ABS-KEY ( "adrenal cortex hormones" ) OR TITLE-ABS-KEY ( agglutinin ) OR TITLE-ABS-KEY ( agglutinins ) OR TITLE-ABS-KEY ( aids ) OR TITLE-ABS-KEY ( amjevita ) OR TITLE-ABS-KEY ( "Anticancer Drug Combination" ) OR TITLE-ABS-KEY ( "Anticancer Drug Combinations" ) OR TITLE-ABS-KEY ( "Antilymphocyte Serum" ) OR TITLE-ABS-KEY ( "antineoplastic combined chemotherapy protocol" ) OR TITLE-ABS-KEY ( "antineoplastic combined chemotherapy protocols" ) OR TITLE-ABS-KEY ( "Antineoplastic Combined Chemotherapy Regimens" ) OR TITLE-ABS-KEY ( "Antineoplastic Drug Combination" ) OR TITLE-ABS-KEY ( "Antineoplastic Drug Combinations" ) OR TITLE-ABS-KEY ( asp015k ) OR TITLE-ABS-KEY ( atlizumab ) OR TITLE-ABS-KEY ( azaserine ) OR TITLE-ABS-KEY ( azathioprine ) OR TITLE-ABS-KEY ( azathioprines ) OR TITLE-ABS-KEY ( baricitinib ) OR TITLE-ABS-KEY ( basiliximab ) OR TITLE-ABS-KEY ( belatacept ) OR TITLE-ABS-KEY ( "beta Type Microcytemia" ) OR TITLE-ABS-KEY ( "beta Type Microcytemias" ) OR TITLE-ABS-KEY ( "Biologic agent" ) OR TITLE-ABS-KEY ( "Biologic agents" ) OR TITLE-ABS-KEY ( "BMS 188667" ) OR TITLE-ABS-KEY ( bms188667 ) OR TITLE-ABS-KEY ( "BMS 224818" ) OR TITLE-ABS-KEY ( bms224818 ) OR TITLE-ABS-KEY ( bms188667 ) OR TITLE-ABS-KEY ( "BMS 188667" ) OR TITLE-ABS-KEY ( bms224818 ) OR TITLE-ABS-KEY ( "BMS 224818" ) OR TITLE-ABS-KEY ( busulfan ) OR TITLE-ABS-KEY ( cancer ) OR TITLE-ABS-KEY ( cancers ) OR TITLE-ABS-KEY ( "car t cell therapy" ) OR TITLE-ABS-KEY ( "car t cell therapies" ) OR TITLE-ABS-KEY ( "CD 20 inhibitor" ) OR TITLE-ABS-KEY ( "CD 20 inhibitors" ) OR TITLE-ABS-KEY ( "CD59 Antigen" ) OR TITLE-ABS-KEY ( "CD59 Antigens" ) OR TITLE-ABS-KEY ( "CDP 870" ) OR TITLE-ABS-KEY ( cdp870 ) OR TITLE-ABS-KEY ( certolizumab ) OR TITLE-ABS-KEY ( "certolizumab pegol" ) OR TITLE-ABS-KEY ( chemotherapy ) OR TITLE-ABS-KEY ( chemotherapies ) OR TITLE-ABS-KEY ( cimzia ) OR TITLE-ABS-KEY ( cladribine ) OR TITLE-ABS-KEY ( "CNTO 148" ) OR TITLE-ABS-KEY ( cnto148 ) OR TITLE-ABS-KEY ( coformycin ) OR TITLE-ABS-KEY ( coformycins ) OR TITLE-ABS-KEY ( "Combined Antineoplastic Agent" ) OR TITLE-ABS-KEY ( "Combined Antineoplastic Agents" ) OR TITLE-ABS-KEY ( "Complement C1 Inactivator Proteins" ) OR TITLE-ABS-KEY ( "Complement C1 Inhibitor Protein" ) OR TITLE-ABS-KEY ( "Complement C3 Nephritic Factor" ) OR TITLE-ABS-KEY ( "Complement C3b Inactivator Proteins" ) OR TITLE-ABS-KEY ( "Complement C4b Binding Protein" ) OR TITLE-ABS-KEY ( "Complement Factor H" ) OR TITLE-ABS-KEY ( "Complement Factor I" ) OR TITLE-ABS-KEY ( "Complement Inactivator Proteins" ) OR TITLE-ABS-KEY ( "Cooley's Anemia" ) OR TITLE-ABS-KEY ( corticoid ) OR TITLE-ABS-KEY ( corticoids ) OR TITLE-ABS-KEY ( corticosteroid ) OR TITLE-ABS-KEY ( corticosteroids ) OR TITLE-ABS-KEY ( "CP 690550" ) OR TITLE-ABS-KEY ( cp690550 ) OR TITLE-ABS-KEY ( "CTLA 4 Inhibitor" ) OR TITLE-ABS-KEY ( "CTLA 4 Inhibitors" ) OR TITLE-ABS-KEY ( "CTLA4 Ig Immunoconjugate" ) OR TITLE-ABS-KEY ( "CTLA4 Fc" ) OR TITLE-ABS-KEY ( "CTLA4 Ig" ) OR TITLE-ABS-KEY ( "CTLA 4 Ig" ) OR TITLE-ABS-KEY ( cyclophosphamide ) OR TITLE-ABS-KEY ( cyclophosphamides ) OR TITLE-ABS-KEY ( cyclosporine ) OR TITLE-ABS-KEY ( cyclosporines ) OR TITLE-ABS-KEY ( cyclosporin ) OR TITLE-ABS-KEY ( cyclosporins ) OR TITLE-ABS-KEY ( cyltezo ) OR TITLE-ABS-KEY ( cytarabine ) OR TITLE-ABS-KEY ( "Cytotoxic T Lymphocyte Associated Antigen 4 Immunoglobulin" ) OR TITLE-ABS-KEY ( "Cytotoxic T Lymphocyte Associated Protein 4 Inhibitor" ) OR TITLE-ABS-KEY ( "Cytotoxic T Lymphocyte Associated Protein 4 Inhibitors" ) OR TITLE-ABS-KEY ( "D2E7 Antibody" ) OR TITLE-ABS-KEY ( daclizumab ) OR TITLE-ABS-KEY ( "Dimethyl Fumarate" ) OR TITLE-ABS-KEY ( ellipticine ) OR TITLE-ABS-KEY ( ellipticines ) OR TITLE-ABS-KEY ( enbrel ) OR TITLE-ABS-KEY ( erelzi ) OR TITLE-ABS-KEY ( "Erythroblastic Anemia" ) OR TITLE-ABS-KEY ( "Erythroblastic Anemias" ) OR TITLE-ABS-KEY ( etanercept ) OR TITLE-ABS-KEY ( "Etanercept szzs" ) OR TITLE-ABS-KEY ( everolimus ) OR TITLE-ABS-KEY ( "Fingolimod Hydrochloride" ) OR TITLE-ABS-KEY ( fluorouracil ) OR TITLE-ABS-KEY ( fluorouracils ) OR TITLE-ABS-KEY ( "Glatiramer Acetate" ) OR TITLE-ABS-KEY ( gliotoxin ) OR TITLE-ABS-KEY ( gliotoxins ) OR TITLE-ABS-KEY ( glucocorticoid ) OR TITLE-ABS-KEY ( glucocorticoids ) OR TITLE-ABS-KEY ( golimumab ) OR TITLE-ABS-KEY ( gp2013 ) OR TITLE-ABS-KEY ( "GP 2013" ) OR TITLE-ABS-KEY ( "Hematopoietic stem cell transplantation" ) OR TITLE-ABS-KEY ( "Hematopoietic stem cell transplantations" ) OR TITLE-ABS-KEY ( "Hemoglobin F disease" ) OR TITLE-ABS-KEY ( "Hemoglobin H disease" ) OR TITLE-ABS-KEY ( "Hemoglobin H diseases" ) OR TITLE-ABS-KEY ( hiv ) OR TITLE-ABS-KEY ( "Human immunodeficiency virus" ) OR TITLE-ABS-KEY ( "Human immunodeficiency viruses" ) OR TITLE-ABS-KEY ( "Human T Cell Leukemia Virus Type III" ) OR TITLE-ABS-KEY ( "Human T Cell Lymphotropic Virus Type III" ) OR TITLE-ABS-KEY ( "Human T Lymphotropic Virus Type III" ) OR TITLE-ABS-KEY ( "Human T-Cell Leukemia Virus Type III" ) OR TITLE-ABS-KEY ( "Human T-Cell Lymphotropic Virus Type III" ) OR TITLE-ABS-KEY ( "Human T-Lymphotropic Virus Type III" ) OR TITLE-ABS-KEY ( humira ) OR TITLE-ABS-KEY ( hydroxycorticosteroid ) OR TITLE-ABS-KEY ( hydroxycorticosteroids ) OR TITLE-ABS-KEY ( "IDEC C2B8" ) OR TITLE-ABS-KEY ( idecc2b8 ) OR TITLE-ABS-KEY ( "IDEC C2B8 Antibody" ) OR TITLE-ABS-KEY ( "Immune Checkpoint Blockade" ) OR TITLE-ABS-KEY ( "Immune Checkpoint Blockers" ) OR TITLE-ABS-KEY ( "Immune Checkpoint Inhibition" ) OR TITLE-ABS-KEY ( "Immune Checkpoint Inhibitions" ) OR TITLE-ABS-KEY ( "Immune Checkpoint Inhibitor" ) OR TITLE-ABS-KEY ( "immune checkpoint inhibitors" ) OR TITLE-ABS-KEY ( immunocompromise ) OR TITLE-ABS-KEY ( immunocompromised ) OR TITLE-ABS-KEY ( "Immunocompromised host" ) OR TITLE-ABS-KEY ( immunodeficiency ) OR TITLE-ABS-KEY ( immunodeficiencies ) OR TITLE-ABS-KEY ( immunodeficient ) OR TITLE-ABS-KEY ( immunodepression ) OR TITLE-ABS-KEY ( immunodepressions ) OR TITLE-ABS-KEY ( "immunologic factor" ) OR TITLE-ABS-KEY ( "immunologic factors" ) OR TITLE-ABS-KEY ( "Immunomodulating agent" ) OR TITLE-ABS-KEY ( "Immunomodulating agents" ) OR TITLE-ABS-KEY ( immunomodulator ) OR TITLE-ABS-KEY ( immunomodulators ) OR TITLE-ABS-KEY ( immunosuppressant ) OR TITLE-ABS-KEY ( immunosuppressants ) OR TITLE-ABS-KEY ( immunosuppressed ) OR TITLE-ABS-KEY ( immunosuppression ) OR TITLE-ABS-KEY ( immunosuppressive ) OR TITLE-ABS-KEY ( "immunosuppressive agents" ) OR TITLE-ABS-KEY ( inflectra ) OR TITLE-ABS-KEY ( infliximab ) OR TITLE-ABS-KEY ( infliximabs ) OR TITLE-ABS-KEY ( interferon ) OR TITLE-ABS-KEY ( interferons ) OR TITLE-ABS-KEY ( "JAK inhibitor" ) OR TITLE-ABS-KEY ( "JAK inhibitors" ) OR TITLE-ABS-KEY ( "Janus Kinase Inhibitor" ) OR TITLE-ABS-KEY ( "Janus Kinase Inhibitors" ) OR TITLE-ABS-KEY ( kevzara ) OR TITLE-ABS-KEY ( "LAV HTLV III" ) OR TITLE-ABS-KEY ( lavhtlviii ) OR TITLE-ABS-KEY ( lea29y ) OR TITLE-ABS-KEY ( leflunomide ) OR TITLE-ABS-KEY ( leukemia ) OR TITLE-ABS-KEY ( ly3009104 ) OR TITLE-ABS-KEY ( "Lymphadenopathy Associated Virus" ) OR TITLE-ABS-KEY ( "Lymphadenopathy-Associated Viruses" ) OR TITLE-ABS-KEY ( lymphoma ) OR TITLE-ABS-KEY ( lymphomas ) OR TITLE-ABS-KEY ( mabthera ) OR TITLE-ABS-KEY ( malignancies ) OR TITLE-ABS-KEY ( malignancy ) OR TITLE-ABS-KEY ( "mast cell stabilizer" ) OR TITLE-ABS-KEY ( "mast cell stabilizers" ) OR TITLE-ABS-KEY ( "Mediterranean Anemia" ) OR TITLE-ABS-KEY ( "Mediterranean Anemias" ) OR TITLE-ABS-KEY ( melphalan ) OR TITLE-ABS-KEY ( mercaptopurine ) OR TITLE-ABS-KEY ( mercaptopurines ) OR TITLE-ABS-KEY ( methotrexate ) OR TITLE-ABS-KEY ( methotrexates ) OR TITLE-ABS-KEY ( "Monoclonal antibody cA2" ) OR TITLE-ABS-KEY ( "Multiple myeloma" ) OR TITLE-ABS-KEY ( "Muromonab CD3" ) OR TITLE-ABS-KEY ( muromonabcd3 ) OR TITLE-ABS-KEY ( neoplasm ) OR TITLE-ABS-KEY ( neoplasms ) OR TITLE-ABS-KEY ( nulojix ) OR TITLE-ABS-KEY ( olumiant ) OR TITLE-ABS-KEY ( orencia ) OR TITLE-ABS-KEY ( "Organ Grafting" ) OR TITLE-ABS-KEY ( "Organ Graftings" ) OR TITLE-ABS-KEY ( "Organ transplantation" ) OR TITLE-ABS-KEY ( "Organ transplantations" ) OR TITLE-ABS-KEY ( "PD 1 Inhibitor" ) OR TITLE-ABS-KEY ( "PD 1 Inhibitors" ) OR TITLE-ABS-KEY ( "PD 1 PD L1 Blockade" ) OR TITLE-ABS-KEY ( "PD L1 Inhibitor" ) OR TITLE-ABS-KEY ( "PD L1 Inhibitors" ) OR TITLE-ABS-KEY ( peficitinib ) OR TITLE-ABS-KEY ( "Programmed Cell Death Protein 1 Inhibitor" ) OR TITLE-ABS-KEY ( "Programmed Cell Death Protein 1 Inhibitors" ) OR TITLE-ABS-KEY ( "Programmed Death Ligand 1 Inhibitor" ) OR TITLE-ABS-KEY ( "Programmed Death Ligand 1 Inhibitors" ) OR TITLE-ABS-KEY ( "R 1569" ) OR TITLE-ABS-KEY ( "Radiation chimera" ) OR TITLE-ABS-KEY ( "Radiation chimeras" ) OR TITLE-ABS-KEY ( "Recombinant Human Dimeric TNF Receptor Type II IgG Fusion Protein" ) OR TITLE-ABS-KEY ( regn88 ) OR TITLE-ABS-KEY ( "REGN 88" ) OR TITLE-ABS-KEY ( remicade ) OR TITLE-ABS-KEY ( renflexis ) OR TITLE-ABS-KEY ( "RHPM 1" ) OR TITLE-ABS-KEY ( rhpm1 ) OR TITLE-ABS-KEY ( rinvoq ) OR TITLE-ABS-KEY ( rituxan ) OR TITLE-ABS-KEY ( rituximab ) OR TITLE-ABS-KEY ( rituximabs ) OR TITLE-ABS-KEY ( "RO 4877533" ) OR TITLE-ABS-KEY ( roactemra ) OR TITLE-ABS-KEY ( sar153191 ) OR TITLE-ABS-KEY ( "SAR 153191" ) OR TITLE-ABS-KEY ( sarilumab ) OR TITLE-ABS-KEY ( simponi ) OR TITLE-ABS-KEY ( sirolimus ) OR TITLE-ABS-KEY ( steroid ) OR TITLE-ABS-KEY ( steroids ) OR TITLE-ABS-KEY ( "T Cell stimulation inhibitor" ) OR TITLE-ABS-KEY ( "T Cell stimulation inhibitors" ) OR TITLE-ABS-KEY ( tacrolimus ) OR TITLE-ABS-KEY ( thalassemia ) OR TITLE-ABS-KEY ( thalassemias ) OR TITLE-ABS-KEY ( thalidomide ) OR TITLE-ABS-KEY ( thalidomides ) OR TITLE-ABS-KEY ( thioinosine ) OR TITLE-ABS-KEY ( thiotepa ) OR TITLE-ABS-KEY ( "TNF antagonist" ) OR TITLE-ABS-KEY ( "TNF antagonists" ) OR TITLE-ABS-KEY ( "TNF blocker" ) OR TITLE-ABS-KEY ( "TNF blockers" ) OR TITLE-ABS-KEY ( "TNF inhibitor" ) OR TITLE-ABS-KEY ( "TNF inhibitors" ) OR TITLE-ABS-KEY ( "TNF Receptor Type II IgG Fusion Protein" ) OR TITLE-ABS-KEY ( "TNFR Fc Fusion Protein" ) OR TITLE-ABS-KEY ( "TNR 001" ) OR TITLE-ABS-KEY ( "TNT Receptor Fusion Protein" ) OR TITLE-ABS-KEY ( tocilizumab ) OR TITLE-ABS-KEY ( tofacitinib ) OR TITLE-ABS-KEY ( "Triamcinolone Acetonide" ) OR TITLE-ABS-KEY ( "Tumor necrosis factor blocker" ) OR TITLE-ABS-KEY ( "Tumor necrosis factor blockers" ) OR TITLE-ABS-KEY ( "tumor necrosis factor inhibitor" ) OR TITLE-ABS-KEY ( "tumor necrosis factor inhibitors" ) OR TITLE-ABS-KEY ( upadacitinib ) OR TITLE-ABS-KEY ( wortmannin ) OR TITLE-ABS-KEY ( wortmannins ) OR TITLE-ABS-KEY ( xeljanz ) ) ) – 8.574 (2019-2023)

**Cochrane**

#44

MeSH descriptor: [COVID-19 Vaccines] explode all trees

MeSH

465

#45

MeSH descriptor: [RNA, Messenger] explode all trees

MeSH

1632

#46

MeSH descriptor: [Vaccines] in all MeSH products

MeSH

16038

#47

MeSH descriptor: [Immunity] in all MeSH products

MeSH

5098

#48

#44 OR #45 OR #46 OR #47

Limits

20813

#49

MeSH descriptor: [COVID-19] explode all trees

MeSH

4737

#50

MeSH descriptor: [SARS-CoV-2] explode all trees

MeSH

2376

#51

MeSH descriptor: [Coronavirus Infections] explode all trees

MeSH

5328

#52

#49 OR #50 OR #51

Limits

5540

#53

MeSH descriptor: [Vaccine Efficacy] explode all trees

MeSH

47

#54

MeSH descriptor: [Immunocompromised Host] explode all trees

MeSH

319

#55

MeSH descriptor: [Organ Transplantation] explode all trees

MeSH

7536

#56

MeSH descriptor: [Hematopoietic Stem Cell Transplantation] explode all trees

MeSH

2141

#57

MeSH descriptor: [HIV] explode all trees

MeSH

3760

#58

MeSH descriptor: [Acquired Immunodeficiency Syndrome] explode all trees

MeSH

2276

#59

MeSH descriptor: [Thalassemia] explode all trees

MeSH

508

#60

MeSH descriptor: [Neoplasms] explode all trees

MeSH

112562

#61

MeSH descriptor: [Antineoplastic Combined Chemotherapy Protocols] explode all trees

MeSH

18559

#62

MeSH descriptor: [Chemotherapy, Adjuvant] explode all trees

MeSH

5303

#63

MeSH descriptor: [Adrenal Cortex Hormones] explode all trees

MeSH

17009

#64

MeSH descriptor: [Glucocorticoids] explode all trees

MeSH

5345

#65

MeSH descriptor: [Hydroxycorticosteroids] explode all trees

MeSH

8029

#66

MeSH descriptor: [17-Ketosteroids] explode all trees

MeSH

1271

#67

MeSH descriptor: [Immunologic Factors] explode all trees

MeSH

10174

#68

MeSH descriptor: [Immunosuppressive Agents] explode all trees

MeSH

6161

#69

MeSH descriptor: [Interferons] explode all trees

MeSH

6498

#70

MeSH descriptor: [Mast Cell Stabilizers] explode all trees

MeSH

1

#71

MeSH descriptor: [Agglutinins] explode all trees

MeSH

142

#72

MeSH descriptor: [Tumor Necrosis Factor Inhibitors] explode all trees

MeSH

128

#73

MeSH descriptor: [Infliximab] explode all trees

MeSH

952

#74

MeSH descriptor: [Adalimumab] explode all trees

MeSH

1000

#75

MeSH descriptor: [Certolizumab Pegol] explode all trees

MeSH

199

#76

MeSH descriptor: [Etanercept] explode all trees

MeSH

924

#77

MeSH descriptor: [Janus Kinase Inhibitors] explode all trees

MeSH

156

#78

MeSH descriptor: [Immune Checkpoint Inhibitors] explode all trees

MeSH

197

#79

MeSH descriptor: [Abatacept] explode all trees

MeSH

358

#80

MeSH descriptor: [Rituximab] explode all trees

MeSH

1734

#81

#54 OR #55 OR #56 OR #57 OR #58 OR #59 OR #60 OR #61 OR #62 OR #63 OR #64 OR #65 OR #66 OR #67 OR #68 OR #69 OR #70 OR #71 OR #72 OR #73 OR #74 OR #75 OR #76 OR #77 OR #79 OR #70

Limits

155604

#82

#48 AND #52 AND #53 AND #81

Limits

3

#83

("Covid 19 Vaccines"):ti,ab,kw OR ("messenger RNA"):ti,ab,kw OR (Vaccines):ti,ab,kw OR ("vaccination"):ti,ab,kw OR ("immunization"):ti,ab,kw

(Word variations have been searched)S Limits

68908

#84

(vaccine):ti,ab,kw OR (Vaccinations):ti,ab,kw OR ("Pfizer"):ti,ab,kw OR (bnt162b2):ti,ab,kw OR (moderna):ti,ab,kw

(Word variations have been searched)S Limits

33870

#85

(immunizations):ti,ab,kw OR (variolation):ti,ab,kw OR (variolations):ti,ab,kw OR ("immunologic stimulation"):ti,ab,kw OR (immunostimulation):ti,ab,kw

(Word variations have been searched)S Limits

52492

#86

("mrna 1273"):ti,ab,kw OR ("chadox1 s"):ti,ab,kw OR (azd1222):ti,ab,kw OR ("astrazeneca oxford"):ti,ab,kw OR (janssen):ti,ab,kw

(Word variations have been searched)S Limits

1902

#87

("johnson johnson"):ti,ab,kw OR (ad26covs1):ti,ab,kw OR ("jnj 78436735"):ti,ab,kw OR (gamaleya):ti,ab,kw OR ("sputnik v"):ti,ab,kw

(Word variations have been searched)S Limits

559

#88

(sinovac):ti,ab,kw OR ("corona vac"):ti,ab,kw OR (novavax):ti,ab,kw OR ("COVID-19 vaccine"):ti,ab,kw

(Word variations have been searched)S Limits

1362

#89

#83 OR #84 OR #85 OR #86 OR #87 OR #88

Limits

72733

#90

("COVID 19"):ti,ab,kw OR ("SARS CoV 2"):ti,ab,kw OR ("Coronavirus infections"):ti,ab,kw OR ("coronavirus disease 2019"):ti,ab,kw OR (covid):ti,ab,kw

(Word variations have been searched)S Limits

17847

#91

("corona virus"):ti,ab,kw OR (coronavirus):ti,ab,kw OR ("2019 ncov"):ti,ab,kw

(Word variations have been searched)S Limits

10569

#92

#90 OR #91

Limits

18110

#93

("vaccine efficacy"):ti,ab,kw OR (effectiveness):ti,ab,kw OR (efficacy):ti,ab,kw OR (effective):ti,ab,kw OR ("serologic response"):ti,ab,kw

(Word variations have been searched)S Limits

1332124

#94

("serologic responses"):ti,ab,kw OR ("immune response"):ti,ab,kw OR ("immune responses"):ti,ab,kw OR ("antibody response"):ti,ab,kw OR ("antibody responses"):ti,ab,kw

(Word variations have been searched)S Limits

17052

#95

("vaccine responses"):ti,ab,kw OR (efficacious):ti,ab,kw OR ("vaccine response"):ti,ab,kw OR ("Response to SARS"):ti,ab,kw

(Word variations have been searched)S Limits

435515

#96

#93 OR #94 OR #95

Limits

1337284

#97

("17 ketosteroid"):ti,ab,kw OR ("17 ketosteroids"):ti,ab,kw OR (abatacept):ti,ab,kw OR ("ABT 494"):ti,ab,kw AND (ABT494):ti,ab,kw

(Word variations have been searched)S Limits

1019

#98

("Acquired immune deficiency syndrome"):ti,ab,kw OR ("acquired immune-deficiency syndromes"):ti,ab,kw OR ("acquired immuno deficiency syndrome"):ti,ab,kw OR ("Acquired immuno deficiency syndromes"):ti,ab,kw OR ("acquired immunodeficiency syndrome"):ti,ab,kw

(Word variations have been searched)S Limits

3508

#99

("Acquired Immunodeficiency Syndromes"):ti,ab,kw OR (Actemra):ti,ab,kw OR (adalimumab):ti,ab,kw OR (adalimumabs):ti,ab,kw OR ("Adalimumab adbm"):ti,ab,kw

(Word variations have been searched)S Limits

6630

#100

("Adalimumab atto"):ti,ab,kw OR ("Adjuvant chemotherapy"):ti,ab,kw OR ("Adjuvant chemotherapies"):ti,ab,kw OR ("adrenal cortex hormone"):ti,ab,kw OR ("adrenal cortex hormones"):ti,ab,kw

(Word variations have been searched)S Limits

13756

#101

(agglutinin):ti,ab,kw OR (agglutinins):ti,ab,kw OR (AIDS):ti,ab,kw OR (Amjevita):ti,ab,kw OR ("Anticancer Drug Combination"):ti,ab,kw

(Word variations have been searched)S Limits

26642

#102

("Anticancer Drug Combinations"):ti,ab,kw OR ("antilymphocyte serum"):ti,ab,kw OR ("antineoplastic combined chemotherapy protocol"):ti,ab,kw OR ("antineoplastic combined chemotherapy protocols"):ti,ab,kw OR ("Antineoplastic Combined Chemotherapy Regimens"):ti,ab,kw

(Word variations have been searched)S Limits

19327

#103

("Antineoplastic Drug Combination"):ti,ab,kw OR ("Antineoplastic Drug Combinations"):ti,ab,kw OR (ASP015K):ti,ab,kw OR (atlizumab):ti,ab,kw OR ("azaserine"):ti,ab,kw

(Word variations have been searched)S Limits

51

#104

("azathioprine"):ti,ab,kw OR (Azathioprines):ti,ab,kw OR (Baricitinib):ti,ab,kw OR ("basiliximab"):ti,ab,kw OR (Belatacept):ti,ab,kw

(Word variations have been searched)S Limits

5295

#105

("beta Type Microcytemia"):ti,ab,kw OR ("beta Type Microcytemias"):ti,ab,kw OR ("Biologic agent"):ti,ab,kw OR ("Biologic agents"):ti,ab,kw OR ("BMS 188667"):ti,ab,kw

(Word variations have been searched)S Limits

904

#106

(BMS188667):ti,ab,kw OR ("BMS 224818"):ti,ab,kw OR (BMS224818):ti,ab,kw OR ("BMS188667"):ti,ab,kw OR ("BMS 188667"):ti,ab,kw

(Word variations have been searched)S Limits

49

#107

(BMS224818):ti,ab,kw OR (Busulfan):ti,ab,kw OR ("cancer"):ti,ab,kw OR (Cancers):ti,ab,kw OR ("car t cell therapy"):ti,ab,kw

(Word variations have been searched)S Limits

200663

#108

("car t cell therapies"):ti,ab,kw OR ("CD-20 inhibitor"):ti,ab,kw OR ("CD-20 inhibitors"):ti,ab,kw OR ("CD59 Antigen"):ti,ab,kw OR ("CD59 Antigens"):ti,ab,kw

(Word variations have been searched)S Limits

143

#109

("CDP 870"):ti,ab,kw OR (CDP870):ti,ab,kw OR (Certolizumab):ti,ab,kw OR ("certolizumab pegol"):ti,ab,kw OR ("chemotherapy"):ti,ab,kw

(Word variations have been searched)S Limits

91795

#110

("chemotherapies"):ti,ab,kw OR (Cimzia):ti,ab,kw OR (Cladribine):ti,ab,kw OR ("CNTO 148"):ti,ab,kw OR (CNTO148):ti,ab,kw

(Word variations have been searched)S Limits

91441

#111

("coformycin"):ti,ab,kw OR (Coformycins):ti,ab,kw OR ("Combined Antineoplastic Agent"):ti,ab,kw OR ("Combined Antineoplastic Agents"):ti,ab,kw OR ("Complement C1 Inactivator Proteins"):ti,ab,kw

(Word variations have been searched)S Limits

263

#112

("Complement C1 Inhibitor Protein"):ti,ab,kw OR ("Complement C3 Nephritic Factor"):ti,ab,kw OR ("Complement C3b Inactivator Proteins"):ti,ab,kw OR ("Complement C4b-Binding Protein"):ti,ab,kw OR ("Complement Factor H"):ti,ab,kw

(Word variations have been searched)S Limits

181

#113

("Complement Factor I"):ti,ab,kw OR ("Complement Inactivator Proteins"):ti,ab,kw OR ("Cooley's Anemia"):ti,ab,kw OR ("corticoid"):ti,ab,kw OR (Corticoids):ti,ab,kw

(Word variations have been searched)S Limits

651

#114

("corticosteroid"):ti,ab,kw OR (Corticosteroids):ti,ab,kw OR ("CP 690550"):ti,ab,kw OR (CP690550):ti,ab,kw OR ("CTLA 4 Inhibitor"):ti,ab,kw

(Word variations have been searched)S Limits

25854

#115

("CTLA 4 Inhibitors"):ti,ab,kw OR ("CTLA4 Ig Immunoconjugate"):ti,ab,kw OR (CTLA4-Fc):ti,ab,kw OR (CTLA4-Ig):ti,ab,kw OR (CTLA-4-Ig):ti,ab,kw

(Word variations have been searched)

Error: this line contains missing or unrequired syntax. See Help for more information.

S Limits

Error

#116

("CTLA 4 Inhibitors"):ti,ab,kw OR ("CTLA4 Ig Immunoconjugate"):ti,ab,kw OR ("CTLA4 Fc"):ti,ab,kw OR ("CTLA4 Ig"):ti,ab,kw OR ("CTLA 4 Ig"):ti,ab,kw

(Word variations have been searched)S Limits

120

#117

("cyclophosphamide"):ti,ab,kw OR (Cyclophosphamides):ti,ab,kw OR (Cyclosporine):ti,ab,kw OR (Cyclosporines):ti,ab,kw OR ("cyclosporin"):ti,ab,kw

(Word variations have been searched)S Limits

20528

#118

(Cyclosporins):ti,ab,kw OR (Cyltezo):ti,ab,kw OR ("cytarabine"):ti,ab,kw OR ("Cytotoxic T Lymphocyte Associated Antigen 4 Immunoglobulin"):ti,ab,kw OR ("Cytotoxic T Lymphocyte Associated Protein 4 Inhibitor"):ti,ab,kw

(Word variations have been searched)S Limits

10810

#119

("Cytotoxic T Lymphocyte Associated Protein 4 Inhibitors"):ti,ab,kw OR ("D2E7 Antibody"):ti,ab,kw OR ("daclizumab"):ti,ab,kw OR ("Dimethyl Fumarate"):ti,ab,kw OR ("ellipticine"):ti,ab,kw

(Word variations have been searched)S Limits

996

#120

(Ellipticines):ti,ab,kw OR (Enbrel):ti,ab,kw OR (Erelzi):ti,ab,kw OR ("erythroblastic anemia"):ti,ab,kw OR ("Erythroblastic Anemias"):ti,ab,kw

(Word variations have been searched)S Limits

254

#121

(etanercept):ti,ab,kw OR ("Etanercept szzs"):ti,ab,kw OR ("everolimus"):ti,ab,kw OR ("Fingolimod Hydrochloride"):ti,ab,kw OR ("fluorouracil"):ti,ab,kw

(Word variations have been searched)S Limits

18617

#122

(Fluorouracils):ti,ab,kw OR ("Glatiramer Acetate"):ti,ab,kw OR ("gliotoxin"):ti,ab,kw OR (Gliotoxins):ti,ab,kw OR ("glucocorticoid"):ti,ab,kw

(Word variations have been searched)S Limits

22544

#123

(glucocorticoids):ti,ab,kw OR (Golimumab):ti,ab,kw OR (GP2013):ti,ab,kw OR ("GP 2013"):ti,ab,kw OR ("Hematopoietic stem cell transplantation"):ti,ab,kw

(Word variations have been searched)S Limits

15730

#124

("Hematopoietic stem cell transplantations"):ti,ab,kw OR ("Hemoglobin F disease"):ti,ab,kw OR ("Hemoglobin H disease"):ti,ab,kw OR ("Hemoglobin H diseases"):ti,ab,kw OR (HIV):ti,ab,kw

(Word variations have been searched)S Limits

35569

#125

("Human immunodeficiency virus"):ti,ab,kw OR ("Human immunodeficiency viruses"):ti,ab,kw OR ("Human T Cell Leukemia Virus Type III"):ti,ab,kw OR ("human T cell lymphotropic virus type III"):ti,ab,kw OR ("Human T Lymphotropic Virus Type III"):ti,ab,kw

(Word variations have been searched)S Limits

13850

#126

("human T cell leukemia virus type III"):ti,ab,kw OR ("human T cell lymphotropic virus type III"):ti,ab,kw OR ("Human T Lymphotropic Virus Type III"):ti,ab,kw OR (Humira):ti,ab,kw OR (hydroxycorticosteroid):ti,ab,kw

(Word variations have been searched)S Limits

547

#127

(hydroxycorticosteroids):ti,ab,kw OR ("IDEC C2B8"):ti,ab,kw OR (IDECC2B8):ti,ab,kw OR ("IDEC C2B8 Antibody"):ti,ab,kw OR ("Immune Checkpoint Blockade"):ti,ab,kw

(Word variations have been searched)S Limits

353

#128

("Immune Checkpoint Blockers"):ti,ab,kw OR ("Immune Checkpoint Inhibition"):ti,ab,kw OR ("Immune Checkpoint Inhibitions"):ti,ab,kw OR ("Immune Checkpoint Inhibitor"):ti,ab,kw OR ("immune checkpoint inhibitors"):ti,ab,kw

(Word variations have been searched)S Limits

1559

#129

(immunocompromise):ti,ab,kw OR ("immunocompromised"):ti,ab,kw OR ("immunocompromised host"):ti,ab,kw OR ("immunodeficiency"):ti,ab,kw OR ("immunodeficiencies"):ti,ab,kw

(Word variations have been searched)S Limits

17735

#130

("immunodeficient"):ti,ab,kw OR (immunodepression):ti,ab,kw OR (immunodepressions):ti,ab,kw OR ("immunologic factor"):ti,ab,kw OR ("immunologic factors"):ti,ab,kw

(Word variations have been searched)S Limits

17941

#131

("Immunomodulating agent"):ti,ab,kw OR ("Immunomodulating agents"):ti,ab,kw OR ("immunomodulator"):ti,ab,kw OR (Immunomodulators):ti,ab,kw OR (immunosuppressant):ti,ab,kw

(Word variations have been searched)S Limits

19929

#132

(immunosuppressants):ti,ab,kw OR (immunosuppressed):ti,ab,kw OR (immunosuppression):ti,ab,kw OR ("immunosuppressive"):ti,ab,kw OR ("immunosuppressive agents"):ti,ab,kw

(Word variations have been searched)S Limits

16772

#133

(Inflectra):ti,ab,kw OR (infliximab):ti,ab,kw OR (infliximabs):ti,ab,kw OR (interferon):ti,ab,kw OR (interferons):ti,ab,kw

(Word variations have been searched)S Limits

19778

#134

("JAK inhibitor"):ti,ab,kw OR ("JAK inhibitors"):ti,ab,kw OR ("Janus Kinase Inhibitor"):ti,ab,kw OR ("janus kinase inhibitors"):ti,ab,kw OR (Kevzara):ti,ab,kw

(Word variations have been searched)S Limits

1421

#135

("LAV HTLV III"):ti,ab,kw OR (LAVHTLVIII):ti,ab,kw OR (LEA29Y):ti,ab,kw OR ("leflunomide"):ti,ab,kw OR ("leukemia"):ti,ab,kw

(Word variations have been searched)S Limits

17048

#136

(LY3009104):ti,ab,kw OR ("lymphadenopathy associated virus"):ti,ab,kw OR ("lymphadenopathy associated viruses"):ti,ab,kw OR ("lymphoma"):ti,ab,kw OR (Lymphomas):ti,ab,kw

(Word variations have been searched)S Limits

13799

#137

(Mabthera):ti,ab,kw OR ("malignancies"):ti,ab,kw OR ("malignancy"):ti,ab,kw OR ("mast cell stabilizer"):ti,ab,kw OR ("mast cell stabilizers"):ti,ab,kw

(Word variations have been searched)S Limits

33031

#138

("Mediterranean anemia"):ti,ab,kw OR ("Mediterranean Anemias"):ti,ab,kw OR ("melphalan"):ti,ab,kw OR ("mercaptopurine"):ti,ab,kw OR (Mercaptopurines):ti,ab,kw

(Word variations have been searched)S Limits

3232

#139

("methotrexate"):ti,ab,kw OR (Methotrexates):ti,ab,kw OR ("Monoclonal antibody cA2"):ti,ab,kw OR ("multiple myeloma"):ti,ab,kw OR ("Muromonab CD3"):ti,ab,kw

(Word variations have been searched)S Limits

19144

#140

(MuromonabCD3):ti,ab,kw OR ("neoplasm"):ti,ab,kw OR (Neoplasms):ti,ab,kw OR (Nulojix):ti,ab,kw OR (Olumiant):ti,ab,kw

(Word variations have been searched)S Limits

107885

#141

(Orencia):ti,ab,kw OR ("Organ Grafting"):ti,ab,kw OR ("Organ Graftings"):ti,ab,kw OR ("Organ transplantation"):ti,ab,kw OR ("organ transplantations"):ti,ab,kw

(Word variations have been searched)S Limits

1689

#142

("PD 1 Inhibitor"):ti,ab,kw OR ("PD 1 Inhibitors"):ti,ab,kw OR ("PD 1 PD L1 Blockade"):ti,ab,kw OR ("PD L1 Inhibitor"):ti,ab,kw OR ("PD L1 Inhibitors"):ti,ab,kw

(Word variations have been searched)S Limits

756

#143

(Peficitinib):ti,ab,kw OR ("Programmed Cell Death Protein 1 Inhibitor"):ti,ab,kw OR ("Programmed Cell Death Protein 1 Inhibitors"):ti,ab,kw OR ("Programmed Death Ligand 1 Inhibitor"):ti,ab,kw OR ("Programmed Death Ligand 1 Inhibitors"):ti,ab,kw

(Word variations have been searched)S Limits

45

#144

("R 1569"):ti,ab,kw OR ("radiation chimera"):ti,ab,kw OR ("Radiation chimeras"):ti,ab,kw OR ("Recombinant Human Dimeric TNF Receptor Type II IgG Fusion Protein"):ti,ab,kw OR (REGN88):ti,ab,kw

(Word variations have been searched)S Limits

32

#145

("REGN 88"):ti,ab,kw OR (Remicade):ti,ab,kw OR (Renflexis):ti,ab,kw OR ("RHPM 1"):ti,ab,kw OR (RHPM1):ti,ab,kw

(Word variations have been searched)S Limits

253

#146

(Rinvoq):ti,ab,kw OR (Rituxan):ti,ab,kw OR (rituximab):ti,ab,kw OR (rituximabs):ti,ab,kw OR ("RO 4877533"):ti,ab,kw

(Word variations have been searched)S Limits

5878

#147

(Roactemra):ti,ab,kw OR (SAR153191):ti,ab,kw OR ("SAR 153191"):ti,ab,kw OR (Sarilumab):ti,ab,kw OR (Simponi):ti,ab,kw

(Word variations have been searched)S Limits

503

#148

("sirolimus"):ti,ab,kw OR ("steroid"):ti,ab,kw OR (Steroids):ti,ab,kw OR ("T Cell stimulation inhibitor"):ti,ab,kw OR ("T Cell stimulation inhibitors"):ti,ab,kw

(Word variations have been searched)S Limits

39829

#149

("tacrolimus"):ti,ab,kw OR ("thalassemia"):ti,ab,kw OR (Thalassemias):ti,ab,kw OR ("thalidomide"):ti,ab,kw OR (Thalidomides):ti,ab,kw

(Word variations have been searched)S Limits

9166

#150

(Thioinosine):ti,ab,kw OR ("thiotepa"):ti,ab,kw OR ("TNF antagonist"):ti,ab,kw OR ("TNF antagonists"):ti,ab,kw OR ("TNF blocker"):ti,ab,kw

(Word variations have been searched)S Limits

844

#151

("TNF blockers"):ti,ab,kw OR ("TNF inhibitor"):ti,ab,kw OR ("TNF inhibitors"):ti,ab,kw OR ("TNF Receptor Type II IgG Fusion Protein"):ti,ab,kw OR ("TNFR Fc Fusion Protein"):ti,ab,kw

(Word variations have been searched)S Limits

971

#152

("TNR 001"):ti,ab,kw OR ("TNT Receptor Fusion Protein"):ti,ab,kw OR (Tocilizumab):ti,ab,kw OR (Tofacitinib):ti,ab,kw OR ("Triamcinolone Acetonide"):ti,ab,kw

(Word variations have been searched)S Limits

5269

#153

("Tumor necrosis factor blocker"):ti,ab,kw OR ("Tumor necrosis factor blockers"):ti,ab,kw OR ("tumor necrosis factor inhibitor"):ti,ab,kw OR ("tumor necrosis factor inhibitors"):ti,ab,kw OR (Upadacitinib):ti,ab,kw

(Word variations have been searched)S Limits

1609

#154

("wortmannin"):ti,ab,kw OR (Wortmannins):ti,ab,kw OR (Xeljanz):ti,ab,kw OR ("hemodialysis"):ti,ab,kw OR ("hematological malignancies"):ti,ab,kw

(Word variations have been searched)S Limits

16847

#155

("hematologic malignancies"):ti,ab,kw OR ("hematologic malignancy"):ti,ab,kw OR ("haematological malignancy"):ti,ab,kw OR ("neutralizing antibody"):ti,ab,kw OR (immunomodulatory):ti,ab,kw

(Word variations have been searched)S Limits

9297

#156

(transplant):ti,ab,kw OR (transplantation):ti,ab,kw OR ("autoimmune inflammatory"):ti,ab,kw

(Word variations have been searched)S Limits

44937

#157

#97 OR #98 OR #99 OR #100 OR #101 OR #102 OR #103 OR #104 OR #105 OR #106 OR #107 OR #108 OR #109 OR #110 OR #111 OR #112 OR #113 OR #114 OR #116 OR #117 OR #118 OR #119 OR #120 OR #121 OR #122 OR #123 OR #124 OR #125 OR #126 OR #127 OR #128 OR #129 OR #130 OR #131 OR #132 OR #133 OR #134 OR #135 OR #136 OR #137 OR #138 OR #139 OR #140 OR #141 OR #142 OR #143 OR #144 OR #145 OR #146 OR #147 OR #148 OR #149 OR #150 OR #151 OR #152 OR #153 OR #154 OR #155 OR #156

Limits

451618

#158

#89 AND #92 AND #96 AND #157

Limits

1181

#159

MeSH descriptor: [Pregnancy, Twin] explode all trees

MeSH

92

#160

MeSH descriptor: [Pregnancy, Multiple] explode all trees

MeSH

323

#161

MeSH descriptor: [Pregnancy, Triplet] explode all trees

MeSH

6

#162

MeSH descriptor: [Pregnancy, Quadruplet] explode all trees

MeSH

0

#163

#159 OR #160 OR #161 OR #162

Limits

323

#164

("twin pregnancy"):ti,ab,kw OR ("twin pregnancies"):ti,ab,kw OR ("Twin gestation"):ti,ab,kw OR ("Twin gestations"):ti,ab,kw OR ("multiple pregnancy"):ti,ab,kw

(Word variations have been searched)S Limits

1917

#165

("multiple pregnancies"):ti,ab,kw OR ("Multiple gestation"):ti,ab,kw OR ("Multiple gestations"):ti,ab,kw OR ("Triplet Pregnancy"):ti,ab,kw OR ("Triplet pregnancies"):ti,ab,kw

(Word variations have been searched)S Limits

1494

#166

("Triplet gestation"):ti,ab,kw OR ("Triplet gestations"):ti,ab,kw OR ("Quadruplet pregnancy"):ti,ab,kw OR ("Quadruplet pregnancies"):ti,ab,kw OR ("Quadruplet gestation"):ti,ab,kw

(Word variations have been searched)S Limits

14

#167

("Triplet gestation"):ti,ab,kw OR ("Triplet gestations"):ti,ab,kw OR ("Quadruplet pregnancy"):ti,ab,kw OR ("Quadruplet pregnancies"):ti,ab,kw OR ("Quadruplet gestation"):ti,ab,kw

(Word variations have been searched)S Limits

14

#168

("Quadruplet gestations"):ti,ab,kw

(Word variations have been searched)S Limits

1

#169

("cesarean section"):ti,ab,kw OR ("Cesarean sections"):ti,ab,kw OR ("cesarean delivery"):ti,ab,kw OR ("Cesarean deliveries"):ti,ab,kw OR ("C-section"):ti,ab,kw

(Word variations have been searched)S Limits

16291

#170

("C-sections"):ti,ab,kw OR ("Abdominal delivery"):ti,ab,kw OR ("Abdominal deliveries"):ti,ab,kw

(Word variations have been searched)S Limits

492

#171

("Induction of Labor"):ti,ab,kw OR ("Induction of Labour"):ti,ab,kw OR ("Induced Labor"):ti,ab,kw OR ("induced labour"):ti,ab,kw AND ("Labor induction"):ti,ab,kw

(Word variations have been searched)S Limits

3205

#172

("Labour induction"):ti,ab,kw

(Word variations have been searched)S Limits

2223

#173

MeSH descriptor: [Cesarean Section] explode all trees

MeSH

4662

#174

MeSH descriptor: [Labor, Induced] explode all trees

MeSH

1406

#175

#163 AND #173 AND #174

Limits

5

#176

#164 OR #165 OR #166 OR #168

Limits

2151

#177

#169 OR #170

Limits

16320

#178

#171 OR #172

Limits

4105

#179

#176 AND #177 AND #178

Limits

55

#180

#175 OR #179

Limits

56

#181

MeSH descriptor: [Vaccine Efficacy] explode all trees

MeSH

47

#182

MeSH descriptor: [Treatment Outcome] explode all trees

MeSH

181861

#183

MeSH descriptor: [Antibodies, Neutralizing] explode all trees

MeSH

780

#184

#181 OR #182 OR #183

Limits

182570

#185

("wortmannin"):ti,ab,kw OR (Wortmannins):ti,ab,kw OR (Xeljanz):ti,ab,kw

(Word variations have been searched)S Limits

32

#186

#97 OR #98 OR #99 OR #100 OR #101 OR #102 OR #103 OR #104 OR #105 OR #106 OR #107 OR #108 OR #109 OR #110 OR #111 OR #112 OR #113 OR #114 OR #116 OR #117 OR #118 OR #119 OR #120 OR #121 OR #122 OR #123 OR #124 OR #125 OR #126 OR #127 OR #128 OR #129 OR #130 OR #131 OR #132 OR #133 OR #134 OR #135 OR #136 OR #137 OR #138 OR #139 OR #140 OR #141 OR #142 OR #143 OR #144 OR #145 OR #146 OR #147 OR #148 OR #149 OR #150 OR #151 OR #152 OR #153 OR #154 OR #184

Limits

562798

#187

(sinovac):ti,ab,kw OR ("corona vac"):ti,ab,kw OR (novavax):ti,ab,kw OR (covaxin):ti,ab,kw OR (sinopharm):ti,ab,kw

(Word variations have been searched)S Limits

276

#188

(vaccinated):ti,ab,kw

(Word variations have been searched)S Limits

31144

#189

#83 OR #84 OR #85 OR #86 OR #87 OR #187 OR #188

Limits

72734

#190

("COVID 19"):ti,ab,kw OR ("SARS CoV 2"):ti,ab,kw OR ("Coronavirus infections"):ti,ab,kw OR (covid):ti,ab,kw OR ("corona virus"):ti,ab,kw

(Word variations have been searched)S Limits

17512

#191

(coronavirus):ti,ab,kw AND ("2019 ncov"):ti,ab,kw

(Word variations have been searched)S Limits

187

#192

#190 or #191

Limits

17522

#193

("vaccine efficacy"):ti,ab,kw OR ("treatment outcome"):ti,ab,kw OR ("Neutralizing Antibodies"):ti,ab,kw OR (effectiveness):ti,ab,kw AND (efficacy):ti,ab,kw (Word variations have been searched)

(Word variations have been searched)S Limits

434285

#194

(effective):ti,ab,kw

(Word variations have been searched)S Limits

1182114

#195

#193 OR #194

Limits

1234026

#196

#186 AND #189 AND #192 AND #195

Limits

920

#197

#48 AND #52 AND #81 AND #184

Limits

32

#198

#196 OR #197

Limits

922

**Cinahl**

Buscar número de ID Termos da busca Opções de busca Ações

S98

S18 AND S19 AND S23 AND S97

Expansores - Aplicar assuntos equivalentes

Modos de busca - Booleano/Frase

Exibir resultados (479)Exibir detalhesEditar

S97

S24 OR S25 OR S26 OR S27 OR S28 OR S29 OR S30 OR S31 OR S32 OR S33 OR S34 OR S35 OR S36 OR S37 OR S38 OR S39 OR S40 OR S41 OR S42 OR S43 OR S44 OR S45 OR S46 OR S47 OR S48 OR S49 OR S50 OR S51 OR S52 OR S53 OR S54 OR S55 OR S56 OR S57 OR S58 OR S59 OR S60 OR S61 OR S62 OR S63 OR S64 OR S65 OR S66 OR S67 OR S68 OR S69 OR S70 OR S71 OR S72 OR S73 OR S74 OR S75 OR S76 OR S77 OR S78 OR S79 OR S80 OR S81 OR S82 OR S83 OR S84 OR S85 OR S86 OR S87 OR S88 OR S89 OR S90 OR S91 OR S92 OR S93 OR S94 OR S95 ...

Expansores - Aplicar assuntos equivalentes

Modos de busca - Booleano/Frase

Exibir resultados (1,204,063)Exibir detalhesEditar

S96

AB "Tumor necrosis factor blockers" OR AB "tumor necrosis factor inhibitor" OR AB "tumor necrosis factor inhibitors" OR AB Upadacitinib OR AB Wortmannin OR AB Wortmannins OR AB Xeljanz

Expansores - Aplicar assuntos equivalentes

Modos de busca - Booleano/Frase

Exibir resultados (871)Exibir detalhesEditar

S95

SU "Tumor necrosis factor blockers" OR SU "tumor necrosis factor inhibitor" OR SU "tumor necrosis factor inhibitors" OR SU Upadacitinib OR SU Wortmannin OR SU Wortmannins OR SU Xeljanz

Expansores - Aplicar assuntos equivalentes

Modos de busca - Booleano/Frase

Exibir resultados (405)Exibir detalhesEditar

S94

TI "Tumor necrosis factor blockers" OR TI "tumor necrosis factor inhibitor" OR TI "tumor necrosis factor inhibitors" OR TI Upadacitinib OR TI Wortmannin OR TI Wortmannins OR TI Xeljanz

Expansores - Aplicar assuntos equivalentes

Modos de busca - Booleano/Frase

Exibir resultados (474)Exibir detalhesEditar

S93

AB "TNF blocker" OR AB "TNF blockers" OR AB "TNF inhibitor" OR AB "TNF inhibitors" OR AB "TNF Receptor Type II IgG Fusion Protein" OR AB "TNFR Fc Fusion Protein" OR AB "TNR 001" OR AB "TNT Receptor Fusion Protein" OR AB Tocilizumab OR AB Tofacitinib OR AB "Triamcinolone Acetonide" OR AB "Tumor necrosis factor blocker"

Expansores - Aplicar assuntos equivalentes

Modos de busca - Booleano/Frase

Exibir resultados (2,924)Exibir detalhesEditar

S92

SU "TNF blocker" OR SU "TNF blockers" OR SU "TNF inhibitor" OR SU "TNF inhibitors" OR SU "TNF Receptor Type II IgG Fusion Protein" OR SU "TNFR Fc Fusion Protein" OR SU "TNR 001" OR SU "TNT Receptor Fusion Protein" OR SU Tocilizumab OR SU Tofacitinib OR SU "Triamcinolone Acetonide" OR SU "Tumor necrosis factor blocker"

Expansores - Aplicar assuntos equivalentes

Modos de busca - Booleano/Frase

Exibir resultados (765)Exibir detalhesEditar

S91

TI "TNF blocker" OR TI "TNF blockers" OR TI "TNF inhibitor" OR TI "TNF inhibitors" OR TI "TNF Receptor Type II IgG Fusion Protein" OR TI "TNFR Fc Fusion Protein" OR TI "TNR 001" OR TI "TNT Receptor Fusion Protein" OR TI Tocilizumab OR TI Tofacitinib OR TI "Triamcinolone Acetonide" OR TI "Tumor necrosis factor blocker"

Expansores - Aplicar assuntos equivalentes

Modos de busca - Booleano/Frase

Exibir resultados (2,271)Exibir detalhesEditar

S90

AB Steroids OR AB "T Cell stimulation inhibitor" OR AB "T Cell stimulation inhibitors" OR AB Tacrolimus OR AB Thalassemia OR AB Thalassemias OR AB Thalidomide OR AB Thalidomides OR AB Thioinosine OR AB Thiotepa OR AB "TNF antagonist" OR AB "TNF antagonists"

Expansores - Aplicar assuntos equivalentes

Modos de busca - Booleano/Frase

Exibir resultados (27,907)Exibir detalhesEditar

S89

SU Steroids OR SU "T Cell stimulation inhibitor" OR SU "T Cell stimulation inhibitors" OR SU Tacrolimus OR SU Thalassemia OR SU Thalassemias OR SU Thalidomide OR SU Thalidomides OR SU Thioinosine OR SU Thiotepa OR SU "TNF antagonist" OR SU "TNF antagonists"

Expansores - Aplicar assuntos equivalentes

Modos de busca - Booleano/Frase

Exibir resultados (20,961)Exibir detalhesEditar

S88

TI Steroids OR TI "T Cell stimulation inhibitor" OR TI "T Cell stimulation inhibitors" OR TI Tacrolimus OR TI Thalassemia OR TI Thalassemias OR TI Thalidomide OR TI Thalidomides OR TI Thioinosine OR TI Thiotepa OR TI "TNF antagonist" OR TI "TNF antagonists"

Expansores - Aplicar assuntos equivalentes

Modos de busca - Booleano/Frase

Exibir resultados (12,298)Exibir detalhesEditar

S87

AB Rituxan OR AB rituximab OR AB rituximabs OR AB RO-4877533 OR AB Roactemra OR AB SAR153191 OR AB SAR-153191 OR AB Sarilumab OR AB Simponi OR AB Sirolimus OR AB Steroid OR AB Steroids

Expansores - Aplicar assuntos equivalentes

Modos de busca - Booleano/Frase

Exibir resultados (28,517)Exibir detalhesEditar

S86

SU Rituxan OR SU rituximab OR SU rituximabs OR SU RO-4877533 OR SU Roactemra OR SU SAR153191 OR SU SAR-153191 OR SU Sarilumab OR SU Simponi OR SU Sirolimus OR SU Steroid OR SU Steroids

Expansores - Aplicar assuntos equivalentes

Modos de busca - Booleano/Frase

Exibir resultados (20,959)Exibir detalhesEditar

S85

TI Rituxan OR TI rituximab OR TI rituximabs OR TI RO-4877533 OR TI Roactemra OR TI SAR153191 OR TI SAR-153191 OR TI Sarilumab OR TI Simponi OR TI Sirolimus OR TI Steroid OR TI Steroids

Expansores - Aplicar assuntos equivalentes

Modos de busca - Booleano/Frase

Exibir resultados (13,492)Exibir detalhesEditar

S84

AB "Programmed Death Ligand 1 Inhibitors" OR AB R-1569 OR AB "Radiation chimera" OR AB "Radiation chimeras" OR AB "Recombinant Human Dimeric TNF Receptor Type II IgG Fusion Protein" OR AB REGN88 OR AB REGN-88 OR AB Remicade OR AB Renflexis OR AB RHPM-1 OR AB RHPM1 OR AB Rinvoq

Expansores - Aplicar assuntos equivalentes

Modos de busca - Booleano/Frase

Exibir resultados (96)Exibir detalhesEditar

S83

SU "Programmed Death Ligand 1 Inhibitors" OR SU R-1569 OR SU "Radiation chimera" OR SU "Radiation chimeras" OR SU "Recombinant Human Dimeric TNF Receptor Type II IgG Fusion Protein" OR SU REGN88 OR SU REGN-88 OR SU Remicade OR SU Renflexis OR SU RHPM-1 OR SU RHPM1 OR SU Rinvoq

Expansores - Aplicar assuntos equivalentes

Modos de busca - Booleano/Frase

Exibir resultados (0)Exibir detalhesEditar

S82

TI "Programmed Death Ligand 1 Inhibitors" OR TI R-1569 OR TI "Radiation chimera" OR TI "Radiation chimeras" OR TI "Recombinant Human Dimeric TNF Receptor Type II IgG Fusion Protein" OR TI REGN88 OR TI REGN-88 OR TI Remicade OR TI Renflexis OR TI RHPM-1 OR TI RHPM1 OR TI Rinvoq

Expansores - Aplicar assuntos equivalentes

Modos de busca - Booleano/Frase

Exibir resultados (38)Exibir detalhesEditar

S81

AB "Organ Graftings" OR AB "Organ transplantation" OR AB "organ transplantations" OR AB "PD 1 Inhibitor" OR AB "PD 1 Inhibitors" OR AB "PD 1 PD L1 Blockade" OR AB "PD L1 Inhibitor" OR AB "PD L1 Inhibitors" OR AB Peficitinib OR AB "Programmed Cell Death Protein 1 Inhibitor" OR AB "Programmed Cell Death Protein 1 Inhibitors" OR AB "Programmed Death Ligand 1 Inhibitor"

Expansores - Aplicar assuntos equivalentes

Modos de busca - Booleano/Frase

Exibir resultados (2,106)Exibir detalhesEditar

S80

SU "Organ Graftings" OR SU "Organ transplantation" OR SU "organ transplantations" OR SU "PD 1 Inhibitor" OR SU "PD 1 Inhibitors" OR SU "PD 1 PD L1 Blockade" OR SU "PD L1 Inhibitor" OR SU "PD L1 Inhibitors" OR SU Peficitinib OR SU "Programmed Cell Death Protein 1 Inhibitor" OR SU "Programmed Cell Death Protein 1 Inhibitors" OR SU "Programmed Death Ligand 1 Inhibitor"

Expansores - Aplicar assuntos equivalentes

Modos de busca - Booleano/Frase

Exibir resultados (4,183)Exibir detalhesEditar

S79

TI "Organ Graftings" OR TI "Organ transplantation" OR TI "organ transplantations" OR TI "PD 1 Inhibitor" OR TI "PD 1 Inhibitors" OR TI "PD 1 PD L1 Blockade" OR TI "PD L1 Inhibitor" OR TI "PD L1 Inhibitors" OR TI Peficitinib OR TI "Programmed Cell Death Protein 1 Inhibitor" OR TI "Programmed Cell Death Protein 1 Inhibitors" OR TI "Programmed Death Ligand 1 Inhibitor"

Expansores - Aplicar assuntos equivalentes

Modos de busca - Booleano/Frase

Exibir resultados (982)Exibir detalhesEditar

S78

AB Methotrexates OR AB "Monoclonal antibody cA2" OR AB "Multiple myeloma" OR AB Muromonab-CD3 OR AB MuromonabCD3 OR AB Neoplasm OR AB Neoplasms OR AB Nulojix OR AB Olumiant OR AB Orencia OR AB "Organ Grafting" OR AB "Organ Graftings

Expansores - Aplicar assuntos equivalentes

Modos de busca - Booleano/Frase

Exibir resultados (213,286)Exibir detalhesEditar

S77

SU Methotrexates OR SU "Monoclonal antibody cA2" OR SU "Multiple myeloma" OR SU Muromonab-CD3 OR SU MuromonabCD3 OR SU Neoplasm OR SU Neoplasms OR SU Nulojix OR SU Olumiant OR SU Orencia OR SU "Organ Grafting" OR SU "Organ Graftings

Expansores - Aplicar assuntos equivalentes

Modos de busca - Booleano/Frase

Exibir resultados (594,063)Exibir detalhesEditar

S76

TI Methotrexates OR TI "Monoclonal antibody cA2" OR TI "Multiple myeloma" OR TI Muromonab-CD3 OR TI MuromonabCD3 OR TI Neoplasm OR TI Neoplasms OR TI Nulojix OR TI Olumiant OR TI Orencia OR TI "Organ Grafting" OR TI "Organ Graftings

Expansores - Aplicar assuntos equivalentes

Modos de busca - Booleano/Frase

Exibir resultados (84,136)Exibir detalhesEditar

S75

AB Lymphomas OR AB Mabthera OR AB Malignancies OR AB Malignancy OR AB "mast cell stabilizer" OR AB "mast cell stabilizers" OR AB "Mediterranean Anemia" OR AB "Mediterranean Anemias" OR AB Melphalan OR AB Mercaptopurine OR AB Mercaptopurines OR AB Methotrexate

Expansores - Aplicar assuntos equivalentes

Modos de busca - Booleano/Frase

Exibir resultados (65,862)Exibir detalhesEditar

S74

SU Lymphomas OR SU Mabthera OR SU Malignancies OR SU Malignancy OR SU "mast cell stabilizer" OR SU "mast cell stabilizers" OR SU "Mediterranean Anemia" OR SU "Mediterranean Anemias" OR SU Melphalan OR SU Mercaptopurine OR SU Mercaptopurines OR SU Methotrexate

Expansores - Aplicar assuntos equivalentes

Modos de busca - Booleano/Frase

Exibir resultados (27,153)Exibir detalhesEditar

S73

TI Lymphomas OR TI Mabthera OR TI Malignancies OR TI Malignancy OR TI "mast cell stabilizer" OR TI "mast cell stabilizers" OR TI "Mediterranean Anemia" OR TI "Mediterranean Anemias" OR TI Melphalan OR TI Mercaptopurine OR TI Mercaptopurines OR TI Methotrexate

Expansores - Aplicar assuntos equivalentes

Modos de busca - Booleano/Frase

Exibir resultados (35,698)Exibir detalhesEditar

S72

AB "janus kinase inhibitors" OR AB Kevzara OR AB LAV-HTLV-III OR AB LAVHTLVIII OR AB LEA29Y OR AB Leflunomide OR AB Leukemia OR AB LY3009104 OR AB "Lymphadenopathy Associated Virus" OR AB "Lymphadenopathy-Associated Viruses" OR AB Lymphoma OR AB Lymphomas

Expansores - Aplicar assuntos equivalentes

Modos de busca - Booleano/Frase

Exibir resultados (38,605)Exibir detalhesEditar

S71

SU "janus kinase inhibitors" OR SU Kevzara OR SU LAV-HTLV-III OR SU LAVHTLVIII OR SU LEA29Y OR SU Leflunomide OR SU Leukemia OR SU LY3009104 OR SU "Lymphadenopathy Associated Virus" OR SU "Lymphadenopathy-Associated Viruses" OR SU Lymphoma OR SU Lymphomas

Expansores - Aplicar assuntos equivalentes

Modos de busca - Booleano/Frase

Exibir resultados (44,205)Exibir detalhesEditar

S70

SU "janus kinase inhibitors" OR SU Kevzara OR SU LAV-HTLV-III OR SU LAVHTLVIII OR SU LEA29Y OR SU Leflunomide OR SU Leukemia OR SU LY3009104 OR SU "Lymphadenopathy Associated Virus" OR SU "Lymphadenopathy-Associated Viruses" OR SU Lymphoma OR SU Lymphomas

Expansores - Aplicar assuntos equivalentes

Modos de busca - Booleano/Frase

Exibir resultados (0)Exibir detalhesEditar

S69

TI "janus kinase inhibitors" OR TI Kevzara OR TI LAV-HTLV-III OR TI LAVHTLVIII OR TI LEA29Y OR TI Leflunomide OR TI Leukemia OR TI LY3009104 OR TI "Lymphadenopathy Associated Virus" OR TI "Lymphadenopathy-Associated Viruses" OR TI Lymphoma OR TI Lymphomas

Expansores - Aplicar assuntos equivalentes

Modos de busca - Booleano/Frase

Exibir resultados (41,277)Exibir detalhesEditar

S68

AB immunosuppressed OR AB immunosuppression OR AB Immunosuppressive OR AB "immunosuppressive agents" OR AB Inflectra OR AB infliximab OR AB infliximabs OR AB interferon OR AB interferons OR AB "JAK inhibitor" OR AB "JAK inhibitors" OR AB "Janus Kinase Inhibitor"

Expansores - Aplicar assuntos equivalentes

Modos de busca - Booleano/Frase

Exibir resultados (31,212)Exibir detalhesEditar

S67

SU immunosuppressed OR SU immunosuppression OR SU Immunosuppressive OR SU "immunosuppressive agents" OR SU Inflectra OR SU infliximab OR SU infliximabs OR SU interferon OR SU interferons OR SU "JAK inhibitor" OR SU "JAK inhibitors" OR SU "Janus Kinase Inhibitor"

Expansores - Aplicar assuntos equivalentes

Modos de busca - Booleano/Frase

Exibir resultados (29,975)Exibir detalhesEditar

S66

TI immunosuppressed OR TI immunosuppression OR TI Immunosuppressive OR TI "immunosuppressive agents" OR TI Inflectra OR TI infliximab OR TI infliximabs OR TI interferon OR TI interferons OR TI "JAK inhibitor" OR TI "JAK inhibitors" OR TI "Janus Kinase Inhibitor"

Expansores - Aplicar assuntos equivalentes

Modos de busca - Booleano/Frase

Exibir resultados (9,494)Exibir detalhesEditar

S65

AB Immunodeficiency OR AB Immunodeficiencies OR AB Immunodeficient OR AB immunodepression OR AB immunodepressions OR AB "immunologic factor" OR AB "immunologic factors" OR AB "Immunomodulating agent" OR AB "Immunomodulating agents" OR AB Immunomodulator OR AB Immunomodulators OR AB immunosuppressant

Expansores - Aplicar assuntos equivalentes

Modos de busca - Booleano/Frase

Exibir resultados (24,850)Exibir detalhesEditar

S64

SU Immunodeficiency OR SU Immunodeficiencies OR SU Immunodeficient OR SU immunodepression OR SU immunodepressions OR SU "immunologic factor" OR SU "immunologic factors" OR SU "Immunomodulating agent" OR SU "Immunomodulating agents" OR SU Immunomodulator OR SU Immunomodulators OR SU immunosuppressant

Expansores - Aplicar assuntos equivalentes

Modos de busca - Booleano/Frase

Exibir resultados (30,821)Exibir detalhesEditar

S63

TI Immunodeficiency OR TI Immunodeficiencies OR TI Immunodeficient OR TI immunodepression OR TI immunodepressions OR TI "immunologic factor" OR TI "immunologic factors" OR TI "Immunomodulating agent" OR TI "Immunomodulating agents" OR TI Immunomodulator OR TI Immunomodulators OR TI immunosuppressant

Expansores - Aplicar assuntos equivalentes

Modos de busca - Booleano/Frase

Exibir resultados (8,345)Exibir detalhesEditar

S62

AB hydroxycorticosteroids OR AB "IDEC C2B8" OR AB "IDEC C2B8 Antibody" OR AB "Immune Checkpoint Blockade" OR AB "Immune Checkpoint Blockers" OR AB "Immune Checkpoint Inhibition" OR AB "Immune Checkpoint Inhibitions" OR AB "Immune Checkpoint Inhibitor" OR AB "immune checkpoint inhibitors" OR AB immunocompromise OR AB Immunocompromised OR AB "Immunocompromised host"

Expansores - Aplicar assuntos equivalentes

Modos de busca - Booleano/Frase

Exibir resultados (10,613)Exibir detalhesEditar

S61

SU hydroxycorticosteroids OR SU "IDEC C2B8" OR SU "IDEC C2B8 Antibody" OR SU "Immune Checkpoint Blockade" OR SU "Immune Checkpoint Blockers" OR SU "Immune Checkpoint Inhibition" OR SU "Immune Checkpoint Inhibitions" OR SU "Immune Checkpoint Inhibitor" OR SU "immune checkpoint inhibitors" OR SU immunocompromise OR SU Immunocompromised OR SU "Immunocompromised host"

Expansores - Aplicar assuntos equivalentes

Modos de busca - Booleano/Frase

Exibir resultados (8,455)Exibir detalhesEditar

S60

TI hydroxycorticosteroids OR TI "IDEC C2B8" OR TI "IDEC C2B8 Antibody" OR TI "Immune Checkpoint Blockade" OR TI "Immune Checkpoint Blockers" OR TI "Immune Checkpoint Inhibition" OR TI "Immune Checkpoint Inhibitions" OR TI "Immune Checkpoint Inhibitor" OR TI "immune checkpoint inhibitors" OR TI immunocompromise OR TI Immunocompromised OR TI "Immunocompromised host"

Expansores - Aplicar assuntos equivalentes

Modos de busca - Booleano/Frase

Exibir resultados (4,334)Exibir detalhesEditar

S59

AB "Hemoglobin H diseases" OR AB HIV OR AB "Human immunodeficiency virus" OR AB "Human immunodeficiency viruses" OR AB "Human T Cell Leukemia Virus Type III" OR AB "Human T Cell Lymphotropic Virus Type III" OR AB "Human T Lymphotropic Virus Type III" OR AB "Human T-Cell Leukemia Virus Type III" OR AB "Human T-Cell Lymphotropic Virus Type III" OR AB "Human T-Lymphotropic Virus Type III" OR AB Humira OR AB hydroxycorticosteroid

Expansores - Aplicar assuntos equivalentes

Modos de busca - Booleano/Frase

Exibir resultados (82,078)Exibir detalhesEditar

S58

SU "Hemoglobin H diseases" OR SU HIV OR SU "Human immunodeficiency virus" OR SU "Human immunodeficiency viruses" OR SU "Human T Cell Leukemia Virus Type III" OR SU "Human T Cell Lymphotropic Virus Type III" OR SU "Human T Lymphotropic Virus Type III" OR SU "Human T-Cell Leukemia Virus Type III" OR SU "Human T-Cell Lymphotropic Virus Type III" OR SU "Human T-Lymphotropic Virus Type III" OR SU Humira OR SU hydroxycorticosteroid

Expansores - Aplicar assuntos equivalentes

Modos de busca - Booleano/Frase

Exibir resultados (102,155)Exibir detalhesEditar

S57

TI "Hemoglobin H diseases" OR TI HIV OR TI "Human immunodeficiency virus" OR TI "Human immunodeficiency viruses" OR TI "Human T Cell Leukemia Virus Type III" OR TI "Human T Cell Lymphotropic Virus Type III" OR TI "Human T Lymphotropic Virus Type III" OR TI "Human T-Cell Leukemia Virus Type III" OR TI "Human T-Cell Lymphotropic Virus Type III" OR TI "Human T-Lymphotropic Virus Type III" OR TI Humira OR TI hydroxycorticosteroid

Expansores - Aplicar assuntos equivalentes

Modos de busca - Booleano/Frase

Exibir resultados (80,449)Exibir detalhesEditar

S56

AB Fluorouracils OR AB "Glatiramer Acetate" OR AB Gliotoxin OR AB Gliotoxins OR AB Glucocorticoid OR AB glucocorticoids OR AB Golimumab OR AB GP2013 OR AB "GP 2013" OR AB "Hematopoietic stem cell transplantation" OR AB "Hematopoietic stem cell transplantations" OR AB "Hemoglobin F disease"

Expansores - Aplicar assuntos equivalentes

Modos de busca - Booleano/Frase

Exibir resultados (12,635)Exibir detalhesEditar

S55

SU Fluorouracils OR SU "Glatiramer Acetate" OR SU Gliotoxin OR SU Gliotoxins OR SU Glucocorticoid OR SU glucocorticoids OR SU Golimumab OR SU GP2013 OR SU "GP 2013" OR SU "Hematopoietic stem cell transplantation" OR SU "Hematopoietic stem cell transplantations" OR SU "Hemoglobin F disease"

Expansores - Aplicar assuntos equivalentes

Modos de busca - Booleano/Frase

Exibir resultados (27,641)Exibir detalhesEditar

S54

TI Fluorouracils OR TI "Glatiramer Acetate" OR TI Gliotoxin OR TI Gliotoxins OR TI Glucocorticoid OR TI glucocorticoids OR TI Golimumab OR TI GP2013 OR TI "GP 2013" OR TI "Hematopoietic stem cell transplantation" OR TI "Hematopoietic stem cell transplantations" OR TI "Hemoglobin F disease"

Expansores - Aplicar assuntos equivalentes

Modos de busca - Booleano/Frase

Exibir resultados (6,176)Exibir detalhesEditar

S53

AB "Dimethyl Fumarate" OR AB Ellipticine OR AB Ellipticines OR AB Enbrel OR AB Erelzi OR AB "Erythroblastic Anemia" OR AB "Erythroblastic Anemias" OR AB etanercept OR AB Etanercept-szzs OR AB Everolimus OR AB "Fingolimod Hydrochloride" OR AB Fluorouracil

Expansores - Aplicar assuntos equivalentes

Modos de busca - Booleano/Frase

Exibir resultados (5,171)Exibir detalhesEditar

S52

SU "Dimethyl Fumarate" OR SU Ellipticine OR SU Ellipticines OR SU Enbrel OR SU Erelzi OR SU "Erythroblastic Anemia" OR SU "Erythroblastic Anemias" OR SU etanercept OR SU Etanercept-szzs OR SU Everolimus OR SU "Fingolimod Hydrochloride" OR SU Fluorouracil

Expansores - Aplicar assuntos equivalentes

Modos de busca - Booleano/Frase

Exibir resultados (7,771)Exibir detalhesEditar

S51

TI "Dimethyl Fumarate" OR TI Ellipticine OR TI Ellipticines OR TI Enbrel OR TI Erelzi OR TI "Erythroblastic Anemia" OR TI "Erythroblastic Anemias" OR TI etanercept OR TI Etanercept-szzs OR TI Everolimus OR TI "Fingolimod Hydrochloride" OR TI Fluorouracil

Expansores - Aplicar assuntos equivalentes

Modos de busca - Booleano/Frase

Exibir resultados (3,300)Exibir detalhesEditar

S50

AB Cyclophosphamides OR AB Cyclosporine OR AB Cyclosporines OR AB Cyclosporin OR AB Cyclosporins OR AB Cyltezo OR AB Cytarabine OR AB "Cytotoxic T Lymphocyte Associated Antigen 4 Immunoglobulin" OR AB "Cytotoxic T Lymphocyte Associated Protein 4 Inhibitor" OR AB "Cytotoxic T Lymphocyte Associated Protein 4 Inhibitors" OR AB "D2E7 Antibody" OR AB Daclizumab

Expansores - Aplicar assuntos equivalentes

Modos de busca - Booleano/Frase

Exibir resultados (9,664)Exibir detalhesEditar

S49

SU Cyclophosphamides OR SU Cyclosporine OR SU Cyclosporines OR SU Cyclosporin OR SU Cyclosporins OR SU Cyltezo OR SU Cytarabine OR SU "Cytotoxic T Lymphocyte Associated Antigen 4 Immunoglobulin" OR SU "Cytotoxic T Lymphocyte Associated Protein 4 Inhibitor" OR SU "Cytotoxic T Lymphocyte Associated Protein 4 Inhibitors" OR SU "D2E7 Antibody" OR SU Daclizumab

Expansores - Aplicar assuntos equivalentes

Modos de busca - Booleano/Frase

Exibir resultados (9,893)Exibir detalhesEditar

S48

TI Cyclophosphamides OR TI Cyclosporine OR TI Cyclosporines OR TI Cyclosporin OR TI Cyclosporins OR TI Cyltezo OR TI Cytarabine OR TI "Cytotoxic T Lymphocyte Associated Antigen 4 Immunoglobulin" OR TI "Cytotoxic T Lymphocyte Associated Protein 4 Inhibitor" OR TI "Cytotoxic T Lymphocyte Associated Protein 4 Inhibitors" OR TI "D2E7 Antibody" OR TI Daclizumab

Expansores - Aplicar assuntos equivalentes

Modos de busca - Booleano/Frase

Exibir resultados (3,641)Exibir detalhesEditar

S47

AB Corticoids OR AB Corticosteroid OR AB Corticosteroids OR AB "CP 690550" OR AB CP690550 OR AB "CTLA 4 Inhibitor" OR AB "CTLA 4 Inhibitors" OR AB "CTLA4 Ig Immunoconjugate" OR AB CTLA4-Fc OR AB CTLA4-Ig OR AB CTLA-4-Ig OR AB Cyclophosphamide

Expansores - Aplicar assuntos equivalentes

Modos de busca - Booleano/Frase

Exibir resultados (26,103)Exibir detalhesEditar

S46

SU Corticoids OR SU Corticosteroid OR SU Corticosteroids OR SU "CP 690550" OR SU CP690550 OR SU "CTLA 4 Inhibitor" OR SU "CTLA 4 Inhibitors" OR SU "CTLA4 Ig Immunoconjugate" OR SU CTLA4-Fc OR SU CTLA4-Ig OR SU CTLA-4-Ig OR SU Cyclophosphamide

Expansores - Aplicar assuntos equivalentes

Modos de busca - Booleano/Frase

Exibir resultados (5,807)Exibir detalhesEditar

S45

TI Corticoids OR TI Corticosteroid OR TI Corticosteroids OR TI "CP 690550" OR TI CP690550 OR TI "CTLA 4 Inhibitor" OR TI "CTLA 4 Inhibitors" OR TI "CTLA4 Ig Immunoconjugate" OR TI CTLA4-Fc OR TI CTLA4-Ig OR TI CTLA-4-Ig OR TI Cyclophosphamide

Expansores - Aplicar assuntos equivalentes

Modos de busca - Booleano/Frase

Exibir resultados (8,688)Exibir detalhesEditar

S44

AB "Combined Antineoplastic Agent" OR AB "Combined Antineoplastic Agents" OR AB "Complement C1 Inactivator Proteins" OR AB "Complement C1 Inhibitor Protein" OR AB "Complement C3 Nephritic Factor" OR AB "Complement C3b Inactivator Proteins" OR AB "Complement C4b-Binding Protein" OR AB "Complement Factor H" OR AB "Complement Factor I" OR AB "Complement Inactivator Proteins" OR AB "Cooley's Anemia" OR AB Corticoid

Expansores - Aplicar assuntos equivalentes

Modos de busca - Booleano/Frase

Exibir resultados (400)Exibir detalhesEditar

S43

SU "Combined Antineoplastic Agent" OR SU "Combined Antineoplastic Agents" OR SU "Complement C1 Inactivator Proteins" OR SU "Complement C1 Inhibitor Protein" OR SU "Complement C3 Nephritic Factor" OR SU "Complement C3b Inactivator Proteins" OR SU "Complement C4b-Binding Protein" OR SU "Complement Factor H" OR SU "Complement Factor I" OR SU "Complement Inactivator Proteins" OR SU "Cooley's Anemia" OR SU Corticoid

Expansores - Aplicar assuntos equivalentes

Modos de busca - Booleano/Frase

Exibir resultados (0)Exibir detalhesEditar

S42

TI "Combined Antineoplastic Agent" OR TI "Combined Antineoplastic Agents" OR TI "Complement C1 Inactivator Proteins" OR TI "Complement C1 Inhibitor Protein" OR TI "Complement C3 Nephritic Factor" OR TI "Complement C3b Inactivator Proteins" OR TI "Complement C4b-Binding Protein" OR TI "Complement Factor H" OR TI "Complement Factor I" OR TI "Complement Inactivator Proteins" OR TI "Cooley's Anemia" OR TI Corticoid

Expansores - Aplicar assuntos equivalentes

Modos de busca - Booleano/Frase

Exibir resultados (142)Exibir detalhesEditar

S41

AB "CD59 Antigens" OR AB "CDP 870" OR AB CDP870 OR AB Certolizumab OR AB "certolizumab pegol" OR AB Chemotherapy OR AB Chemotherapies OR AB Cimzia OR AB Cladribine OR AB "CNTO 148" OR AB CNTO148 OR AB Coformycin

Expansores - Aplicar assuntos equivalentes

Modos de busca - Booleano/Frase

Exibir resultados (71,761)Exibir detalhesEditar

S40

SU "CD59 Antigens" OR SU "CDP 870" OR SU CDP870 OR SU Certolizumab OR SU "certolizumab pegol" OR SU Chemotherapy OR SU Chemotherapies OR SU Cimzia OR SU Cladribine OR SU "CNTO 148" OR SU CNTO148 OR SU Coformycin

Expansores - Aplicar assuntos equivalentes

Modos de busca - Booleano/Frase

Exibir resultados (37,466)Exibir detalhesEditar

S39

TI "CD59 Antigens" OR TI "CDP 870" OR TI CDP870 OR TI Certolizumab OR TI "certolizumab pegol" OR TI Chemotherapy OR TI Chemotherapies OR TI Cimzia OR TI Cladribine OR TI "CNTO 148" OR TI CNTO148 OR TI Coformycin

Expansores - Aplicar assuntos equivalentes

Modos de busca - Booleano/Frase

Exibir resultados (30,527)Exibir detalhesEditar

S38

AB BMS188667 OR AB "BMS 188667" OR AB BMS224818 OR AB "BMS 224818" OR AB Busulfan OR AB Cancer OR AB Cancers OR AB "car t cell therapy" OR AB "car t cell therapies" OR AB "CD-20 inhibitor" OR AB "CD-20 inhibitors" OR AB "CD59 Antigen"

Expansores - Aplicar assuntos equivalentes

Modos de busca - Booleano/Frase

Exibir resultados (351,285)Exibir detalhesEditar

S37

SU BMS188667 OR SU "BMS 188667" OR SU BMS224818 OR SU "BMS 224818" OR SU Busulfan OR SU Cancer OR SU Cancers OR SU "car t cell therapy" OR SU "car t cell therapies" OR SU "CD-20 inhibitor" OR SU "CD-20 inhibitors" OR SU "CD59 Antigen"

Expansores - Aplicar assuntos equivalentes

Modos de busca - Booleano/Frase

Exibir resultados (124,949)Exibir detalhesEditar

S36

TI BMS188667 OR TI "BMS 188667" OR TI BMS224818 OR TI "BMS 224818" OR TI Busulfan OR TI Cancer OR TI Cancers OR TI "car t cell therapy" OR TI "car t cell therapies" OR TI "CD-20 inhibitor" OR TI "CD-20 inhibitors" OR TI "CD59 Antigen"

Expansores - Aplicar assuntos equivalentes

Modos de busca - Booleano/Frase

Exibir resultados (354,432)Exibir detalhesEditar

S35

AB Azathioprines OR AB Baricitinib OR AB Basiliximab OR AB Belatacept OR AB "beta Type Microcytemia" OR AB "beta Type Microcytemias" OR AB "Biologic agent" OR AB "Biologic agents" OR AB "BMS 188667" OR AB BMS188667 OR AB "BMS 224818" OR AB BMS224818

Expansores - Aplicar assuntos equivalentes

Modos de busca - Booleano/Frase

Exibir resultados (3,359)Exibir detalhesEditar

S34

SU Azathioprines OR SU Baricitinib OR SU Basiliximab OR SU Belatacept OR SU "beta Type Microcytemia" OR SU "beta Type Microcytemias" OR SU "Biologic agent" OR SU "Biologic agents" OR SU "BMS 188667" OR SU BMS188667 OR SU "BMS 224818" OR SU BMS224818

Expansores - Aplicar assuntos equivalentes

Modos de busca - Booleano/Frase

Exibir resultados (1,658)Exibir detalhesEditar

S33

TI Azathioprines OR TI Baricitinib OR TI Basiliximab OR TI Belatacept OR TI "beta Type Microcytemia" OR TI "beta Type Microcytemias" OR TI "Biologic agent" OR TI "Biologic agents" OR TI "BMS 188667" OR TI BMS188667 OR TI "BMS 224818" OR TI BMS224818

Expansores - Aplicar assuntos equivalentes

Modos de busca - Booleano/Frase

Exibir resultados (1,075)Exibir detalhesEditar

S32

AB "Anticancer Drug Combination" OR AB "Anticancer Drug Combinations" OR AB "Antilymphocyte Serum" OR AB "antineoplastic combined chemotherapy protocol" OR AB "antineoplastic combined chemotherapy protocols" OR AB "Antineoplastic Combined Chemotherapy Regimens" OR AB "Antineoplastic Drug Combination" OR AB "Antineoplastic Drug Combinations" OR AB ASP015K OR AB atlizumab OR AB Azaserine OR AB Azathioprine

Expansores - Aplicar assuntos equivalentes

Modos de busca - Booleano/Frase

Exibir resultados (1,680)Exibir detalhesEditar

S31

SU "Anticancer Drug Combination" OR SU "Anticancer Drug Combinations" OR SU "Antilymphocyte Serum" OR SU "antineoplastic combined chemotherapy protocol" OR SU "antineoplastic combined chemotherapy protocols" OR SU "Antineoplastic Combined Chemotherapy Regimens" OR SU "Antineoplastic Drug Combination" OR SU "Antineoplastic Drug Combinations" OR SU ASP015K OR SU atlizumab OR SU Azaserine OR SU Azathioprine

Expansores - Aplicar assuntos equivalentes

Modos de busca - Booleano/Frase

Exibir resultados (1,970)Exibir detalhesEditar

S30

TI "Anticancer Drug Combination" OR TI "Anticancer Drug Combinations" OR TI "Antilymphocyte Serum" OR TI "antineoplastic combined chemotherapy protocol" OR TI "antineoplastic combined chemotherapy protocols" OR TI "Antineoplastic Combined Chemotherapy Regimens" OR TI "Antineoplastic Drug Combination" OR TI "Antineoplastic Drug Combinations" OR TI ASP015K OR TI atlizumab OR TI Azaserine OR TI Azathioprine

Expansores - Aplicar assuntos equivalentes

Modos de busca - Booleano/Frase

Exibir resultados (438)Exibir detalhesEditar

S29

AB adalimumab OR AB adalimumabs OR AB "Adalimumab adbm" OR AB "Adalimumab atto" OR AB "Adjuvant chemotherapy" OR AB "Adjuvant chemotherapies" OR AB "adrenal cortex hormone" OR AB "adrenal cortex hormones" OR AB agglutinin OR AB agglutinins OR AB AIDS OR AB Amjevita

Expansores - Aplicar assuntos equivalentes

Modos de busca - Booleano/Frase

Exibir resultados (55,230)Exibir detalhesEditar

S28

SU adalimumab OR SU adalimumabs OR SU "Adalimumab adbm" OR SU "Adalimumab atto" OR SU "Adjuvant chemotherapy" OR SU "Adjuvant chemotherapies" OR SU "adrenal cortex hormone" OR SU "adrenal cortex hormones" OR SU agglutinin OR SU agglutinins OR SU AIDS OR SU Amjevita

Expansores - Aplicar assuntos equivalentes

Modos de busca - Booleano/Frase

Exibir resultados (70,953)Exibir detalhesEditar

S27

TI adalimumab OR TI adalimumabs OR TI "Adalimumab adbm" OR TI "Adalimumab atto" OR TI "Adjuvant chemotherapy" OR TI "Adjuvant chemotherapies" OR TI "adrenal cortex hormone" OR TI "adrenal cortex hormones" OR TI agglutinin OR TI agglutinins OR TI AIDS OR TI Amjevita

Expansores - Aplicar assuntos equivalentes

Modos de busca - Booleano/Frase

Exibir resultados (31,585)Exibir detalhesEditar

S26

AB "17 ketosteroid" OR AB "17 ketosteroids" OR AB abatacept OR AB "ABT 494" OR AB ABT494 OR AB "Acquired immune deficiency syndrome" OR AB "Acquired immune deficiency syndromes" OR AB "Acquired immuno deficiency syndrome" OR AB "Acquired immuno deficiency syndromes" OR AB "Acquired Immunodeficiency Syndrome" OR AB "Acquired Immunodeficiency Syndromes" OR AB Actemra

Expansores - Aplicar assuntos equivalentes

Modos de busca - Booleano/Frase

Exibir resultados (4,997)Exibir detalhesEditar

S25

SU "17 ketosteroid" OR SU "17 ketosteroids" OR SU abatacept OR SU "ABT 494" OR SU ABT494 OR SU "Acquired immune deficiency syndrome" OR SU "Acquired immune deficiency syndromes" OR SU "Acquired immuno deficiency syndrome" OR SU "Acquired immuno deficiency syndromes" OR SU "Acquired Immunodeficiency Syndrome" OR SU "Acquired Immunodeficiency Syndromes" OR SU Actemra

Expansores - Aplicar assuntos equivalentes

Modos de busca - Booleano/Frase

Exibir resultados (22,113)Exibir detalhesEditar

S24

TI "17 ketosteroid" OR TI "17 ketosteroids" OR TI abatacept OR TI "ABT 494" OR TI ABT494 OR TI "Acquired immune deficiency syndrome" OR TI "Acquired immune deficiency syndromes" OR TI "Acquired immuno deficiency syndrome" OR TI "Acquired immuno deficiency syndromes" OR TI "Acquired Immunodeficiency Syndrome" OR TI "Acquired Immunodeficiency Syndromes" OR TI Actemra

Expansores - Aplicar assuntos equivalentes

Modos de busca - Booleano/Frase

Exibir resultados (1,310)Exibir detalhesEditar

S23

S20 OR S21 OR S22

Expansores - Aplicar assuntos equivalentes

Modos de busca - Booleano/Frase

Exibir resultados (1,036,003)Exibir detalhesEditar

S22

AB "vaccine efficacy" OR AB "treatment outcome" OR AB "Neutralizing Antibodies" OR AB effectiveness OR AB efficacy OR AB effective

Expansores - Aplicar assuntos equivalentes

Modos de busca - Booleano/Frase

Exibir resultados (838,862)Exibir detalhesEditar

S21

SU "vaccine efficacy" OR SU "treatment outcome" OR SU "Neutralizing Antibodies" OR SU effectiveness OR SU efficacy OR SU effective

Expansores - Aplicar assuntos equivalentes

Modos de busca - Booleano/Frase

Exibir resultados (218,504)Exibir detalhesEditar

S20

TI "vaccine efficacy" OR TI "treatment outcome" OR TI "Neutralizing Antibodies" OR TI effectiveness OR TI efficacy OR TI effective

Expansores - Aplicar assuntos equivalentes

Modos de busca - Booleano/Frase

Exibir resultados (193,639)Exibir detalhesEditar

S19

S15 OR S16 OR S17

Expansores - Aplicar assuntos equivalentes

Modos de busca - Booleano/Frase

Exibir resultados (159,017)Exibir detalhesEditar

S18

S6 OR S7 OR S8 OR S9 OR S10 OR S11 OR S12 OR S13 OR S14

Expansores - Aplicar assuntos equivalentes

Modos de busca - Booleano/Frase

Exibir resultados (107,650)Exibir detalhesEditar

S17

AB "COVID 19" OR AB "SARS CoV 2" OR AB "Coronavirus infections" OR AB covid OR AB "corona virus" OR AB coronavirus OR AB "2019 ncov"

Expansores - Aplicar assuntos equivalentes

Modos de busca - Booleano/Frase

Exibir resultados (93,677)Exibir detalhesEditar

S16

SU "COVID 19" OR SU "SARS CoV 2" OR SU "Coronavirus infections" OR SU covid OR SU "corona virus" OR SU coronavirus OR SU "2019 ncov"

Expansores - Aplicar assuntos equivalentes

Modos de busca - Booleano/Frase

Exibir resultados (101,383)Exibir detalhesEditar

S15

TI "COVID 19" OR TI "SARS CoV 2" OR TI "Coronavirus infections" OR TI covid OR TI "corona virus" OR TI coronavirus OR TI "2019 ncov"

Expansores - Aplicar assuntos equivalentes

Modos de busca - Booleano/Frase

Exibir resultados (105,885)Exibir detalhesEditar

S14

AB "sputnik v" OR AB sinovac OR AB "corona vac" OR AB novavax OR AB covaxin OR AB sinopharm OR AB vaccinated

Expansores - Aplicar assuntos equivalentes

Modos de busca - Booleano/Frase

Exibir resultados (7,302)Exibir detalhesEditar

S13

SU "sputnik v" OR SU sinovac OR SU "corona vac" OR SU novavax OR SU covaxin OR SU sinopharm OR SU vaccinated

Expansores - Aplicar assuntos equivalentes

Modos de busca - Booleano/Frase

Exibir resultados (0)Exibir detalhesEditar

S12

TI "sputnik v" OR TI sinovac OR TI "corona vac" OR TI novavax OR TI covaxin OR TI sinopharm OR TI vaccinated

Expansores - Aplicar assuntos equivalentes

Modos de busca - Booleano/Frase

Exibir resultados (1,029)Exibir detalhesEditar

S11

AB variolations OR AB "immunologic stimulation" OR AB immunostimulation OR AB "mrna 1273" OR AB "chadox1 s" OR AB azd1222 OR AB "astrazeneca oxford" OR AB janssen OR AB "johnson johnson" OR AB ad26covs1 OR AB "jnj 78436735" OR AB gamaleya

Expansores - Aplicar assuntos equivalentes

Modos de busca - Booleano/Frase

Exibir resultados (1,113)Exibir detalhesEditar

S10

SU variolations OR SU "immunologic stimulation" OR SU immunostimulation OR SU "mrna 1273" OR SU "chadox1 s" OR SU azd1222 OR SU "astrazeneca oxford" OR SU janssen OR SU "johnson johnson" OR SU ad26covs1 OR SU "jnj 78436735" OR SU gamaleya

Expansores - Aplicar assuntos equivalentes

Modos de busca - Booleano/Frase

Exibir resultados (0)Exibir detalhesEditar

S9

TI variolations OR TI "immunologic stimulation" OR TI immunostimulation OR TI "mrna 1273" OR TI "chadox1 s" OR TI azd1222 OR TI "astrazeneca oxford" OR TI janssen OR TI "johnson johnson" OR TI ad26covs1 OR TI "jnj 78436735" OR TI gamaleya

Expansores - Aplicar assuntos equivalentes

Modos de busca - Booleano/Frase

Exibir resultados (354)Exibir detalhesEditar

S8

AB "Covid-19 Vaccines" OR AB "Messenger RNA" OR AB Vaccines OR AB Vaccination OR AB Immunization OR AB vaccine OR AB Vaccinations OR AB pfizer OR AB bnt162b2 OR AB moderna OR AB immunizations OR AB variolation

Expansores - Aplicar assuntos equivalentes

Modos de busca - Booleano/Frase

Exibir resultados (58,937)Exibir detalhesEditar

S7

SU "Covid-19 Vaccines" OR SU "Messenger RNA" OR SU Vaccines OR SU Vaccination OR SU Immunization OR SU vaccine OR SU Vaccinations OR SU pfizer OR SU bnt162b2 OR SU moderna OR SU immunizations OR SU variolation

Expansores - Aplicar assuntos equivalentes

Modos de busca - Booleano/Frase

Exibir resultados (73,887)Exibir detalhesEditar

S6

TI "Covid-19 Vaccines" OR TI "Messenger RNA" OR TI Vaccines OR TI Vaccination OR TI Immunization OR TI vaccine OR TI Vaccinations OR TI pfizer OR TI bnt162b2 OR TI moderna OR TI immunizations OR TI variolation

Expansores - Aplicar assuntos equivalentes

Modos de busca - Booleano/Frase

Exibir resultados (50,527)Exibir detalhesEditar

S5

S1 AND S2 AND S3 AND S4

Expansores - Aplicar assuntos equivalentes

Modos de busca - Booleano/Frase

Executar novamenteExibir detalhesEditar

S4

(MH "Immunocompromised Host") OR (MH "Organ Transplantation") OR (MH "Hematopoietic Stem Cell Transplantation") OR (MH "Human Immunodeficiency Virus") OR (MH "Acquired Immunodeficiency Syndrome") OR (MH "Thalassemia") OR (MH "Neoplasms") OR (MH "Chemotherapy, Adjuvant") OR (MH "Adrenal Cortex Hormones") OR (MH "Glucocorticoids") OR (MH "Immunologic Factors") OR (MH "Immunosuppressive Agents") OR (MH "Interferons") OR (MH "Tumor Necrosis Factor Inhibitors") OR (MH "Infliximab") OR (MH "Adalimumab ...

Expansores - Aplicar assuntos equivalentes

Modos de busca - Booleano/Frase

Executar novamenteExibir detalhesEditar

S3

(MH "Vaccine Efficacy") OR (MH "Treatment Outcomes") OR (MH "Neutralizing Antibodies")

Expansores - Aplicar assuntos equivalentes

Modos de busca - Booleano/Frase

Executar novamenteExibir detalhesEditar

S2

(MH "COVID-19") OR (MH "SARS-CoV-2") OR (MH "Coronavirus Infections")

Expansores - Aplicar assuntos equivalentes

Modos de busca - Booleano/Frase

Executar novamenteExibir detalhesEditar

S1

(MH "COVID-19 Vaccines") OR (MH "RNA, Messenger") OR (MH "Vaccines") OR (MH "Immunization")

Expansores - Aplicar assuntos equivalentes

Modos de busca - Booleano/Frase

Executar novamenteExibir detalhesEdita
